# Early evolution of enamel matrix proteins is reflected by pleiotropy of physiological functions

**DOI:** 10.1038/s41598-023-28388-4

**Published:** 2023-01-26

**Authors:** Frantisek Spoutil, Goretti Aranaz-Novaliches, Michaela Prochazkova, Tomas Wald, Vendula Novosadova, Petr Kasparek, Radim Osicka, Janne E. Reseland, Staale P. Lyngstadaas, Hanna Tiainen, Kristyna Bousova, Jiri Vondrasek, Radislav Sedlacek, Jan Prochazka

**Affiliations:** 1 Czech Centre for Phenogenomics and Laboratory of Transgenic Models of Diseases, Institute of Molecular Genetics of the CAS, Vestec, Czech Republic; 2 Program in Craniofacial Biology and Department of Orofacial Sciences, University of California, San Francisco, CA USA; 3 Laboratory of Molecular Biology of Bacterial Pathogens, Institute of Microbiology of the CAS, Prague, Czech Republic; 4 Department of Biomaterials, Institute of Clinical Dentistry, University of Oslo, Oslo, Norway; 5 Institute of Organic Chemistry and Biochemistry of the CAS, Prague, Czech Republic

**Keywords:** Evolution, Physiology

## Abstract

Highly specialized enamel matrix proteins (EMPs) are predominantly expressed in odontogenic tissues and diverged from common ancestral gene. They are crucial for the maturation of enamel and its extreme complexity in multiple independent lineages. However, divergence of EMPs occured already before the true enamel evolved and their conservancy in toothless species suggests that non-canonical functions are still under natural selection. To elucidate this hypothesis, we carried out an unbiased, comprehensive phenotyping and employed data from the International Mouse Phenotyping Consortium to show functional pleiotropy of amelogenin, ameloblastin, amelotin, and enamelin, genes, i.e. in sensory function, skeletal morphology, cardiovascular function, metabolism, immune system screen, behavior, reproduction, and respiratory function. Mice in all KO mutant lines, i.e. amelogenin KO, ameloblastin KO, amelotin KO, and enamelin KO, as well as mice from the lineage with monomeric form of ameloblastin were affected in multiple physiological systems. Evolutionary conserved motifs and functional pleiotropy support the hypothesis of role of EMPs as general physiological regulators. These findings illustrate how their non-canonical function can still effect the fitness of modern species by an example of influence of amelogenin and ameloblastin on the bone physiology.

## Introduction

The evolutionary history of life provides exciting examples of how natural selection can specialize organs to diverse functions and morphologies^1,2^. However, the connection between evolutionary stabilized phenotype, the responsible genes, and their molecular function remains mostly enigmatic^3^. The evolution of specialized organs is driven by precise amino acid modifications in proteins, which sustain organ unique functions. Such highly specialized proteins can be found for example in the eye lens, where they provide optical properties to eye^4^, or in cilia^5^ or myoblasts^6,7^, where they provide unique structural and mechanical characteristics of the cells. One of the most striking examples is a set of enamel matrix proteins (EMPs), a group of highly specialized secreted proteins essential for orchestrating the biomineralization process during enamel formation in vertebrates. EMPs have a unique, essential function in amelogenesis in mammals regardless of their level of representation in amelogenesis^8–14^. They are part of an evolutionarily conserved family of Secretory Calcium-Binding Phosphoproteins (SCPPs), which arose from a common ancestral gene^15–17^. However, phylogenetic analysis of EMPs’ sequences indicates a discrepancy between the divergence of EMPs^15,18^ and the evolutionary origin of enamel. Based on molecular clocks, EMPs had already started to diverge around the Cambrian explosion^17,18^, however true enamel is documented in fossil records much later in the Silurian period^19^.

This suggests that molecular evolution of EMPs was originally driven by other physiological functions rather than formation of enamel. Already Girondot and Sire^20^ tested this possibility, EMPs may have another role than production of enamel matrix, e.g. modulating calcium metabolism demonstrated by its deposition in bone tissues^21,22^. This can be highlighted by the theory of the Cambrian explosion when calcium storage became an important evolutionary advantage^23^. Deposition of calcium as hydroxyapatite (HAp) in tooth enamel by EMPs might be explained as a derived and highly specialized adaptation of the general calcium regulatory machinery that is still apparent in physiological regulatory networks.

The involvement of EMPs in conserved physiological regulations can be inferred from conservancy of the gene sequences within species, where the evolutionary loss of enamel or even entire dentition occurred. The mutual combination of evolutionary conserved protein sequences, and *in-silico* prediction of functional motifs can identify putative interactions in non-canonical function^24^.

Another piece of evidence emerges from reverse genetic approaches, which allow standardized multi-parameter annotation of gene functions. Data from mouse models in this regard are generated and openly available under the umbrella of the International Mouse Phenotyping Consortium (IMPC). The level of gene function pleiotropy can indicate more complexity in regulatory networks, which will keep a gene under natural selection even after the reduction of enamel and thus it might provide insight into the ancestral functions of ancient EMP genes.

This work proposes a novel hypothesis which elucidates how proteins with unique physiological properties could evolve from genes with broader regulatory functions. We demonstrate by a combination of phylogenetic analysis, in-silico function motif prediction, and reverse genetic approaches, that molecular function of specialized proteins can be tracked back in evolutionary history. A detailed bone physiology analysis of *Ambn* and *Amelx* mutant mice, which revealed significant changes in bone ultrastructure, illustrates how the function of enamel specialized genes can still influence general fitness of modern species, independent of recent dominant function.

## Results

### EMPs show resistance to evolutionary degradation

**Figure 1:**
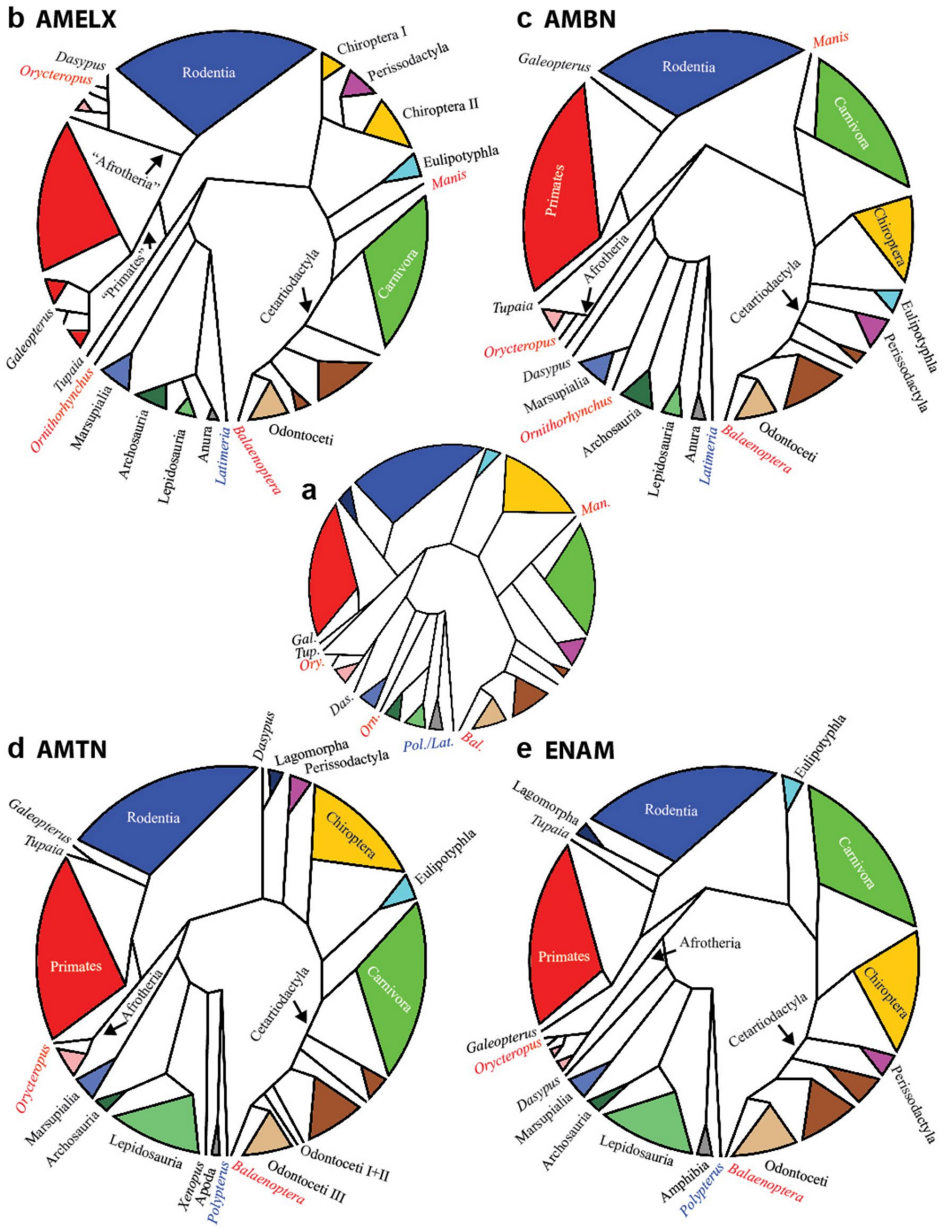
Schemes of unrooted, majority consensus trees of four major enamel matrix proteins. General phylogenetic consensus (www.timetree.org 2021/07/27) as a scheme in the center (**a**) and schematic phylograms for Amelogenin (AMELX—**b**), Ameloblastin (AMBN—**c**), Amelotin (AMTN—**d**), and Enamelin (ENAM—**e**) are displayed highlighting clustering of protein sequences to higher taxonomical groups. Enamel- and toothless species in red, the most basal species (i.e. *Latimeria* or *Polypterus*) in blue. Clusters in bracelets or with a Roman number (I, II, III) are not homogenous in the analysis. There is great stability not only of major taxonomic groups and general tree topology, but also the absence of enamel does not influence the position of the species. Colors correspond to taxonomic clades: Amphibia (dark grey), Lepidosauria (green), Archosauria (dark green); Marsupialia (sea blue), Afrotheria (pink), Primates (red), Lagomorpha (dark blue), Rodentia (blue), Eulipotyphla (light blue), Chiroptera (yellow), Carnivora (light green), Perissodactyla (violet), “Artiodactyla” (brown), Odontoceti (light brown). Full phylograms are in Supplementary Fig. 1–4.

To reveal evolutionary conservancy of EMPs parsimony-based, unrooted phylogenetic analysis was performed to uncover evolutionary relations and the stability of protein variants across lineages. We focused on species where enamel or complete dentition is lost. The highly specialized EMPs with a unique physiological function were expected to cluster at the base of the phylogram by long branch attraction in species with compromised dentition, as their structure should degenerate by random mutations. Despite our expectation, EMPs retain a strong phylogenetic signal, as most of the clusters are in agreement with general taxonomic consensus (Fig. 1, and Supplementary Fig. 1–4). Interestingly, all major phylogenetic lineages are already well defined in the phylograms of all four analyzed EMPs in non-mammalian groups, i.e. amphibians (although AMTN sequences from Apoda are closer to *Polypterus*’ sequence than the one of *Xenopus*), and reptilians (with exception of AMBN, where are sequences of Archosauria and Lepidosauria separated).

In mammalian protein sequence alignments, we observed some alterations from the mammalian phylogenetic consensus, but a correlation with obvious dental morphotypes was not evident (Fig. 1). Marsupialia sequences are always separated from those of Placentalia, despite convergences in animals’ diets and dental apparatus (e.g. *Monodelphis* and *Tupaia*). Sequences of Placentalia always split into two large clusters, Euarchontoglires sequences and Laurasiatheria sequences.

There are several specific alterations in phylogenic signals for the AMELX and AMTN sequences, which are rather independent of dentition properties. The AMELX protein sequence (Fig. 1b) from Perissodactyla, which typically exhibit massive enamel with Hunter–Schreger bands, clustered together with the chiropteran cluster, of which possess a typical simple, thin radial enamel^25^. Another example is from the AMELX sequences of Afrotheria and *Dasypus*, which cluster together with the Euarchontoglires sequences. The AMTN protein sequence from *Dasypus* (degenerative, thin enamel)^26^ clustered together with Lagomorpha (complex enamel microstructure)^25^, which are both close to the cluster of the AMTN sequences (Fig. 1d) of Laurasiatheria, while Afrotheria sequences are closer to the cluster of the remaining Euarchontoglires sequences.

Phylogenetic signal is strongly conserved also in mammals, where sequences of EMPs are grouped within the major taxonomic clusters of which they belong to, regardless of enamel or tooth lost (i.e. *Balaenoptera*, *Manis*, *Ornithorhynchus,* and *Orycteropus*), i.e. no long branch attraction is detected in such cases. The lack of long branch attraction in EMPs sequences can be demonstrated on two sister groups of Mysticeti (*Baleanoptera*) and Odontoceti *(Orcinus)* despite complete loss of functional teeth in Mysticeti. The EMPs sequences still keep strong phylogenic bound to Odontoceti, where teeth are very well preserved and important for predatory life strategy. Moreover, both Mysticeti without functional teeth and Odontoceti with simplified unicuspid teeth with only radial enamel, cluster according to EMPs sequences together within Cetartiortiodactyla (or Artiodactyla sensu Prothero et al.^27^), with multicuspid teeth with highly organized enamel with Hunter-Schreger bands^25^. Such observation fully corresponds to monophylogenetic status of Cetartiodactyla rather than to effect of dentition specialization on EMPs sequences.

We detected highly conserved amino acid sequences within all four EMPs in toothless species or species with compromised enamel, which appeared to be under strong, stabilizing selection during mammalian evolution, independent of dental specialization. In addition, some conserved sequences can be traced back even further in the evolution of whole Sarcopterygii and Osteichthyes lineages. Such observations support the theory of the non-canonical roles of EMPs in more general physiological processes over entire vertebrate evolution^22^.Therefore, EMPs are highly likely to sustain yet unknown physiological functions.

### EMPs possess evolutionary stable motifs with a putative function in protein interaction and signaling

**Figure 2:**
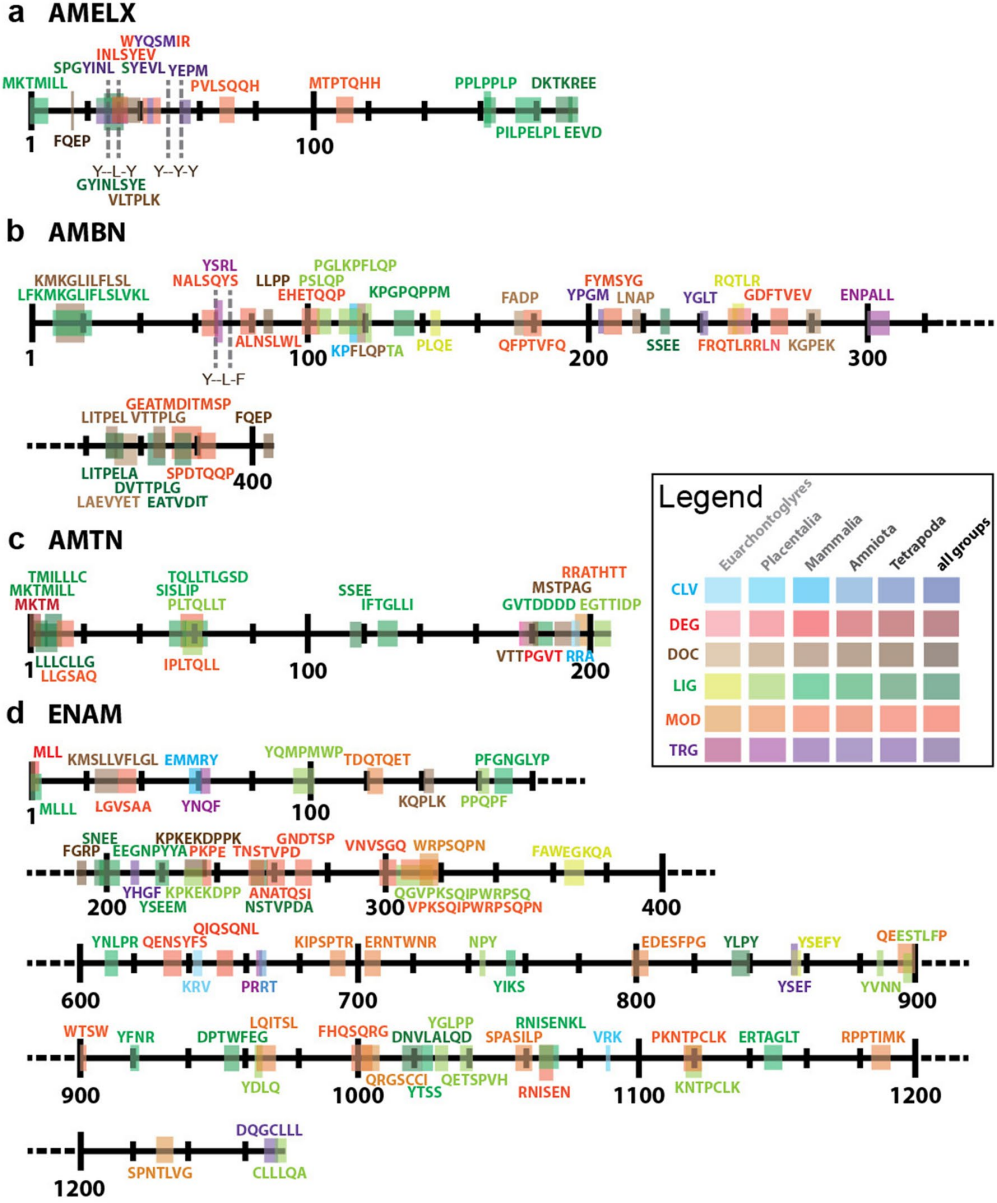
Identification of functional motives of EMPs. Detected functional motifs of AMELX (**a**), AMBN (**b**), AMTN (**c**), and ENAM (**d**). N-terminus to left, C-terminus to right. The position and motif structure corresponds to *Mus musculus* sequences for these proteins (AAB93765.1, BAA06546.1, NP_082069.1, and AAB94312.1). Color of the motif corresponds to the motif type (CLV = cleavage, DEG = degradation, DOC = docking, LIG = ligand, MOD = modifier, TRG = transport), while saturation to the minimal age of the motif, i.e. for how large taxonomical unit (Euarchontoglyres < Placentalia < Mammalia < Amniota < Tetrapoda < Sarcopterygii/Euteleostomi) is it common. The self-assembly motifs are highlighted in the AMELX (Y–L–Y; and Y–Y–Y) and AMBN (Y–L–F) structure. Detailed information about motifs in Supplementary table 1.

To investigate whether conservative sequences bear molecular functions that might be under evolutionary selection, we followed up with an in-silico search of functional motifs within the mouse sequences of all four EMPs, and compared the results with aligned sequences from other species. The mouse sequences of all four proteins are rich in motifs, with the p-value for potential motif presence reached level of 0.01. Their distribution and level of conservancy is shown in Fig. 2. AMELX and AMBN show the highest motif density (according to protein length), whereas ENAM has the lowest motif density despite displaying the highest number of motifs due to its length (over 1200 aa).

As we focused on more general function of the proteins, we searched for the motifs that are present in all taxonomical groups (i.e. are common for Teleostei/Sarcopterygyii), in Tetrapoda, in Amniota, in Mammalia, in Placentalia, or at least in Euarchontoglyres (i.e. clade containing mostly primates and rodents including human and mouse). We investigated in detail EMPs conserved sequences in mammalian species lacking enamel, as these are the organisms providing vital evidence of evolutionary conserved non-canonical functions in EMPs.

The motifs are mostly involved in protein–protein interactions. The proteins contain e.g. multiple sites for SH2 or SH3 domain interaction (28–31 in AMELX, 114–121, 131–137, and 131–138 in AMBN, 95–99, 95–101, 218–222, 228–235, 611–615, 753–756, 837–840, 885–888, 919–922, 963–966, and 1024–1027 in ENAM—see Supplementary table 1). Other motifs that are involved in protein–protein interactions are the PDZ-binding motif of ENAM (1269–1274), acting in protein interaction and sorting at the cytosolic side of the plasma membrane^28^, or the WW domains of AMELX (161–164 and 164–167). Besides the protein interaction surfaces, the conserved sequence also possesses multiple sites for phosphorylation from various kinase families, e.g. for a proline dependent kinase like MAPK in AMBN (10–18) and ENAM (23–32). Another signaling regulatory kinase, PIKK, is predicted to phosphorylate motifs in: 68–74 and 110–116 in AMELX; 62–68, 98–104, and 380–386 in AMBN; 55–61 in AMTN; and 120–126, 258–264, 306–312, 313–319, 650–656, and 998–1004 in ENAM. Motif for calcineurin binding site found at 84–87 and 216–219 of AMBN can be involved in dephosphorylation instead. For the complete list of conserved motifs, see Supplementary table 1 and their localization in Fig. 2.

We identified specific motifs that had degenerated in all species without enamel, however, they were also found to be lost in examples from taxa with fully preserved enamel, e.g. Plk4 kinase phosphorylation motif and TRP ligand motif of AMELX (41–48, and 193–196)—see Supplementary table 1. Interestingly, quite a high number of functional motifs had vanished in groups such as large, modern herbivores, i.e. Equidae and Ruminantia with complex enamel microstructure^25^, e.g. calcineurin binding site of AMBN (216–219) or PKA AGC kinase phosphorylation motif of AMTN (195–201)—see Supplementary table 1. Thus, we can conclude that there is no strict divergence of EMP functions from more general physiological regulation to highly specialized roles in enamel formation. It seems likely that functional motifs within protein sequences are still under broader evolutionary selection not limited to enamel development.

### EMPs have pleiotropic effects on physiology regulation

**Figure 3:**
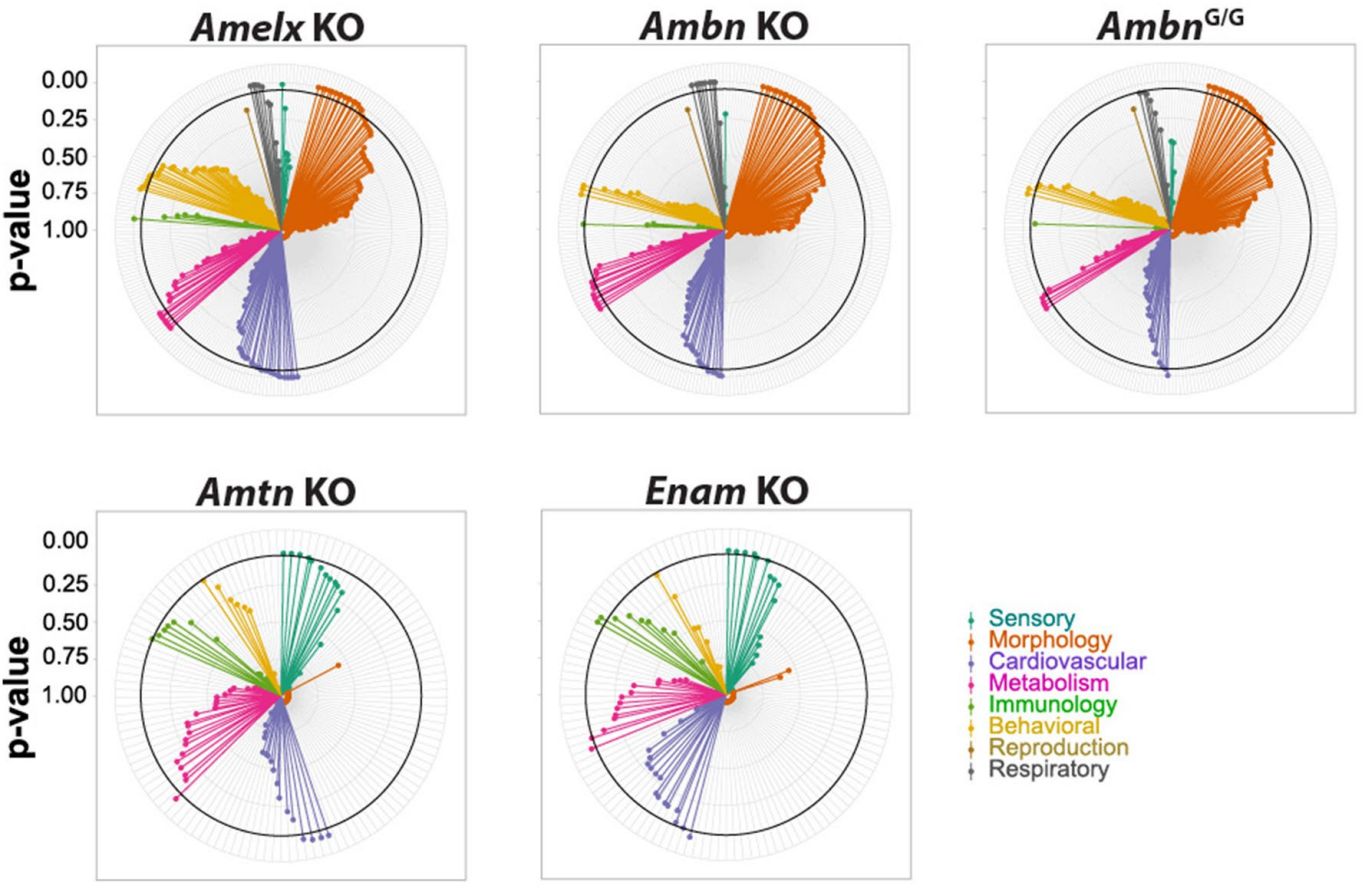
Functional pleiotropy of EMPs revealed by unbiased phenotyping. Circular graphs visualizing significant difference of mouse mutants from WT baseline divided to 8 groups phenotypes: sensory (e.g. hearing or eye retina morphology—in blue-green), morphology (skeletal morphology and bone mineralization—in orange), cardiovascular (e.g. blood pressure, cardiography—in violet), metabolism (e.g. body mass, lean/fat ratio, glucose tolerance—in magenta), immunology (T-cells and B-cells populations characterization—in green), behavioral (e.g. grip strength, dark/light box test, hot/cold plate test—in yellow), reproduction (i.e. number of litters—in brown), and respiratory (e.g. capacity and elasticity of lungs—in gray). Each radius is one variable. Thick black circle marks the level of significance = 0.05—points outside the circle are significant results. The massive impact of all mutations on various levels of phenotype was evident. *Amelx* KO, *Ambn* KO, and *Ambn*^*G/G*^, and were phenotyped in the Czech Centre for Phenogenomics (Vestec, Czech Republic), *Amtn* KO, and *Enam* KO in UC Davis (California, USA). Overview of statistical results is in Supplementary table 2.

Functional ablation of EMPs genes followed by unbiased comprehensive phenotyping is an important tool for the understanding of pleiotropic functions of proteins in physiological regulatory networks. Thus, we analyzed KO phenotypes of enamel matrix genes (*Ambn*, *Amelx*, *Amtn*, *Enam*) according to IMPC guidelines, with IMPC deposited data. We also included the *Ambn*^*G/G*^ variant, which lacks the self-assembly motif and has the monomer structure^13^. A comparison of *Ambn* KO with *Ambn*^*G/G*^ shall shed more light on how essential the self-assembly motif is in general *Ambn* physiological function. For better orientation, we distributed phenotyping parameters to central physiological areas, such as: Sensory, Morphology, Cardiovascular, Metabolism, Immunology, Behavioral, Reproduction, and Respiratory. The parameters from the individual areas are listed in Supplementary table 2 and the strength of pleiotropy is visualized in Fig. 3. We arranged all parameters according to significant alteration from the WT baseline control. The analysis should highlight areas of protein’s non-canonical function worthy of closer attention.

From the phenotype overview in individual mutants with altered EMPs genes, there is an obvious trend observed in phenotype hits corresponding to the assumed evolutionary history of EMPs.

The broadest pleiotropy is observed in *Ambn* KO and *Ambn*^*G/G*^ mutant that also exhibit certain overlap in their phenotype (Supplementary table 2) which clearly shows the importance of the self-assembly motif for proper *Ambn* function, not only in enamel formation as published before^13^, but also in other physiological functions. Both *Ambn* mutant lines show phenotypical similarities covering skeletal-muscular function, red blood cell differentiation and heart physiology. Both lines also exhibit reduced growth in the juvenile period, which might correspond to the alterations in metabolism regulation observed in both lines. Glucose clearing was afflicted in *Ambn*^*G/G*^ mice only, however immune system regulation was affected in both *Ambn* mutant lines. Nonetheless, due to the complexity of immune system differentiation and maturation, interpretation of this causality is beyond the focus of the current work (Fig. 3).

Although the previously mentioned parameters were affected in both variants or were even more strongly deviated in the *Ambn*^*G/G*^ variant, lung function was more influenced in *Ambn* KO than in *Ambn*^*G/G*^ (Fig. 3). This observation might be an indication that in lung physiology self-assembly is only partly needed and monomeric AMBN or its fragments play an important role in lung physiology.

There was a striking overlap of phenotype hits between *Ambn* KO and *Amelx* KO, such as neuromuscular functions, heart physiology, red blood cell homeostasis, and interestingly, the influence on metabolism regulation and lung function. Since the *Amelx* gene evolved by a duplication event from the *Ambn* gene^16,17^, the phenotyping data (Supplementary table 2) also suggests that the role of AMELX in physiological regulatory networks might be adopted from common AMBN and AMELX ancestor protein.

The phenotype pleiotropy for *Enam* and *Amtn* is lower than both *Ambn* and *Amelx* mutant lines, which might be connected to the fact that they diversified earlier from the lineage, leading to *Ambn* and *Amelx*’s ancestor gene^16,17^. There is still a possible alteration in heart function, however, the severity and distribution of the altered parameters are much lower, and significance was reached only in ECG parameters. An interesting split in the distribution of metabolism related parameters can be observed in *Enam* KO and *Amtn* KO lines. Lower lean mass and reduced glucose clearing efficiency was detected in *Enam* KO, contrary to the *Amtn* KO line, which only showed mild changes in liver-based metabolism. The phenotype pleiotropy for *Enam* and *Amtn* is lower than both *Ambn* and *Amelx* mutant lines, which might be connected to the fact that they diversified earlier from the lineage, leading to *Ambn* and *Amelx*’s ancestor genes for a more specific function in tooth enamel, and their non-canonical function might have been reduced or disappeared completely since still compensated by *Ambn* and *Amelx*. In addition, the phenotype severity and distribution can reflect the previously suggested evolutionary history of the EMP family^16,17^. The observed phenotypes in physiology can also explain their evolutionary stability, which might not be exclusive of enamel formation but rather of a more general mechanism. This could for example involve calcium homeostasis or calcium signaling regulation, as most of the effects can be linked directly or indirectly with calcium-related processes^29^.

As the main physiological calcium regulatory organ in the vertebrate body is the skeleton, we focused further on the impact of the ablation of *Ambn* and *Amelx* in bones to delineate possible effects on evolutionary fitness and thus at least partly explain their evolutionary stability in toothless species.

### *Ambn* and *Amelx* KO mutant mice show degradation of bone microstructure

Regarding the bones, we first studied the expression of the *Amelx* and *Ambn* genes in mice femurs, vertebra, and calvaria by RT-PCR and immunohistochemistry for detection of protein with spatial resolution (Supplementary Fig. 5). Our data were in agreement with previously published Ambn expression in bones based on RT-PCR analysis^30,31^ or promoter activity detection by LacZ reporter^32,33^.

**Figure 4:**
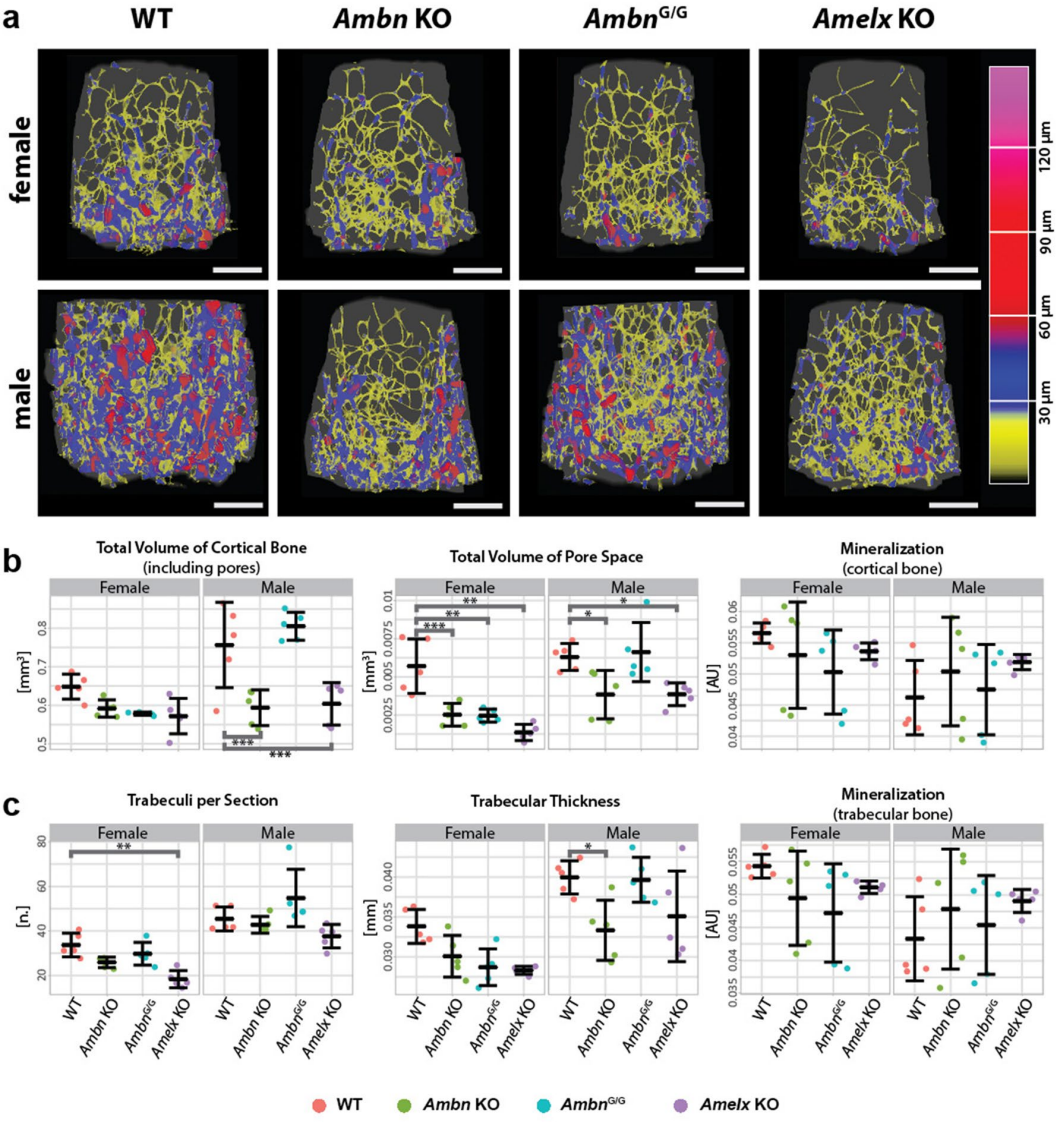
Bone ultrastructure is affected in Ambn and AmelX KO. (**a**) Trabecular structure. Pseudocolors correspond to trabecular thickness (spectrum on the right). Bar = 0.5 mm. Specimens closest to group’s average selected. (**b**) Whisker plots of femoral cortical bone microstructure: total volume of cortical bone (including pores), total volume of pore space, both in mm3, and mineralization of cortical bone in Attenuation units (AU). (**c**) Whisker plot of femoral trabecular bone: mean number of trabeculae per section, the mean thickness of trabeculae in mm, and mineralization of trabecular bone in AU. Females on the left and males on the right of each graph. Thick midline = mean, whiskers = standard deviation; * < 0.05; ** < 0.01, *** < 0.001. Values for each observation are shown as points—WTs in red, *Ambn* KO in green, *Ambn*^*G/G*^ in blue, and *Amelx* KO in violet.

**Figure 5:**
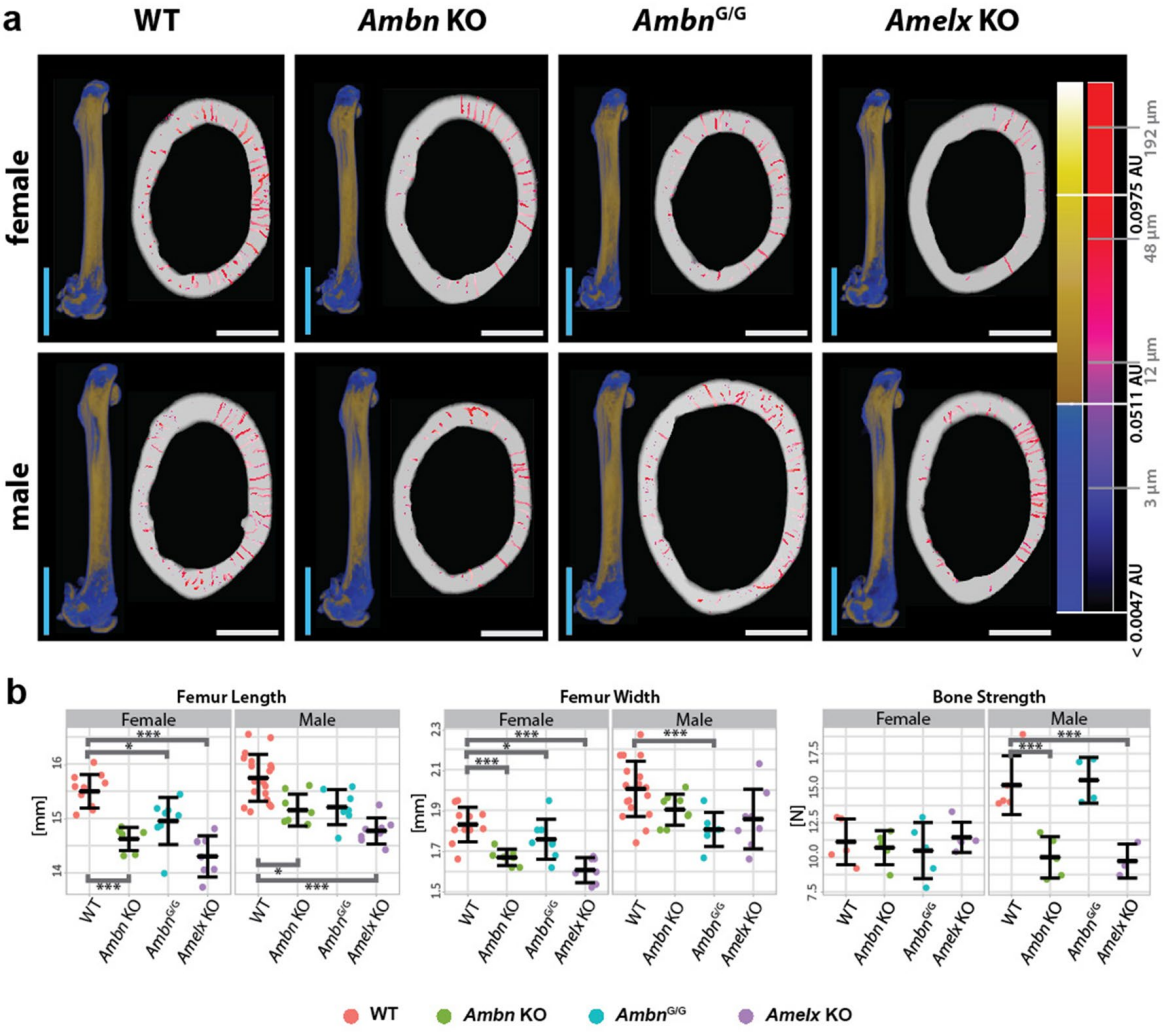
Long bone morphology and strength in Ambn and AmelX mutant mice. (**a**) Structure of femur—bone morphology (35 µm voxel resolution) on the left and porosity of the cortical bone (1.5 µm voxel resolution) on the right. Pseudo colors correspond to differences in mineralization in the whole femur (blue–yellow spectrum), or the pore size in the section (violet–red spectrum). Blue vertical bar (whole femur) = 2 mm, white horizontal bar (section with pores) = 0.5 mm. (**b**) Whisker plots of femur morphometry and bone fracture analysis characteristics: femur length, femur width (both in mm), and bone strength (in Newtons, N). Females on the left and males on the right of each graph. Thick midline = mean, whiskers = standard deviation; *< 0.05, ***< 0.001. Values for each observation are shown as points—WTs in red, *Ambn* KO in green, *Ambn*^*G/G*^ in blue, and *Amelx* KO in violet.

As both genes are expressed in bone tissue and in the bone marrow microenvironment, we assumed that lack of gene function could cause bone structure alteration. We proceeded with detailed high-resolution microCT scanning of long bones and vertebra to analyze their ultrastructure in trabecular and cortical areas (Supplementary Fig. 6). A high-resolution microCT scan is necessary, as lower X-ray absorption found in the whole body scans above may be caused either by lower mineral content per se or by changes in bone ultrastructure, which is beyond the detection limit of low-resolution microCT scans. Interestingly, both *Ambn* KO and *Amelx* KO mutants of both sexes showed changes in bone ultrastructure with the most prominent effect in males. The effect was stronger in long bones, i.e. femurs, than in vertebra, i.e. L4 (see Supplementary table 3 and 4, and Figs. 4, 5a and Supplementary Fig. 7, original data in Supplementary table 5). There is a significant drop in total volume of pore space in the femurs of all *Ambn* KO and *Amelx* KO mutants (Fig. 5a and Supplementary Fig. 8). The femoral cortical bone volume is reduced significantly in *Ambn* KO and *Amelx* KO males (Figs. 4b, 5a) meaning that the average bone thickness is smaller, as the length of the sample was always consistent. There are significant changes in femoral trabeculae of *Ambn* KO and *Amelx* KO mutants too (Fig. 4): trabeculae of *Ambn* KO males are significantly thinner, whereas those of *Amelx* KO females have a lower density (smaller number per section). In the L4 vertebra, there is a significant reduction only in the cortical bone volume of *Amelx* KO females (Supplementary Fig. 7) and in trabecular thickness of *Ambn* KO males (Supplementary Fig. 7).

Since the effect on bone morphology was strong in *Ambn* KO mice, we asked whether the *Ambn* function in long bones might be dependent on its self-assembly capacity and formation of multimeric protein structures. Thus, we also analyzed a mutant *Ambn*^*G/G*^ mouse. Although we have reported some significant differences common with *Ambn*^*G/G*^ and *Ambn* KO mutants in the general phenotyping procedure above, almost no parameter in microstructural bone analysis of *Ambn*^*G*/*G*^ strain was clearly distinct from WT control mice. There was only one exception: pore volume in femurs of females (Supplementary table 3 and 4, Fig. 4b, and Supplementary Fig. 8). This strongly suggests that AMBN molecular mechanism in bone homeostasis is likely to be different from the molecular function of AMBN in tooth enamel development^13^.

As a summary from the detailed bone analysis in *Ambn* KO and *Amelx* KO mice, we conclude: (1) *Ambn* and *Amelx* are important for proper bone homeostasis; (2) the phenotype is much stronger in males; (3) *Amelx* affects bone growth, which is visible by altered bone morphology, while *Ambn* ablation effect is sex-dependent; (4) the bone ultrastructure is affected similarly in *Ambn* KO and *Amelx* KO; (5) the self-assembly motif in AMBN is dispensable for proper bone homeostasis, which underlines a non-canonical function of *Ambn* in bone physiology.

Our results confirm, that the roles of the conserved parts of AMBN and AMELX are not limited to the structural and self-assembly properties important for proper enamel formation.

### Lack of AMBN and AMELX affects fitness of males by decreasing bone strength

There is an important question whether the changes in bone ultrastructure are large enough to reduce the fitness of an animal and thus, animals with such a phenotype might be under negative selective pressure. To answer this, we tested the general mechanical properties of femurs as a representative of long bones.

We proceeded 3D morphometric quantification of mouse right femurs in cohort data parallel to the bone fracture analysis by 3-point bend test fixture. Total femur length and femur width in the central diaphysis were measured in that respect. There were significant differences in body length between strains of mice with the exception of *Ambn*^G/G^ males (Supplementary table 3). All mutant females reach significantly lower values from WTs also in both femur dimensions, while for males the same trends as for body length are valid only for femur length. Femur width is significantly lower only in *Ambn*^G/G^ group of males (Fig. 5 and Supplementary table 3).

Interestingly, the effect of all the microstructural changes mentioned above (i.e. lower porosity and thinner cortical bones) had an effect only on the bone strength of *Ambn* KO and *Amelx* KO males (Fig. 5b and Supplementary table 3). The bone morphology had no effect, as strength of femurs of *Ambn*^G/G^ males was comparable to WT males, and length of bones corresponds to size of an animal. The force needed for breaking them was ^1^/_3_ lower compared to WT males. The bone strength in *Ambn* KO and *Amelx* KO dropped down to level in females (Fig. 5b), which suggest that both *Ambn* and *Amelx* ablation diminished intersexual differences in bone strength due to more frequent injuries in the young age and thus might affect the more competitive sex leading to the removal of these mutations from wild populations. This can serve as one specific case of the pleiotropic effect of EMPs mutant phenotypes, described in higher detail previously. Effects in other tissues can be expected to be additive causing much stronger negative selection against total degradation of EMPs in toothless species.

## Discussion

EMPs are generally known as highly specialized proteins, which are essential for the formation of tooth enamel, the hardest tissue in the vertebrate body. The molecular evolution of such specialized proteins might be obscured by random evolutionary processes, preadaptation events, and conserved pleiotropic effects within complex physiological functions. All such characteristics interfere with evolutionary selective pressure, which continuously fine tunes the protein’s function for highly specialized physiological roles. Therefore, understanding evolutionary constraints in protein sequences can help us to reconstruct the evolutionary history of protein specialization, and more importantly, understand preadaptation mechanisms in the evolution of individual genes and gene families.

In the example of EMPs, we can estimate the preadaptation on the molecular and physiological level, with the original purpose connected to general calcium homeostasis that later adopted a novel specialized function, which become a dominant driver of natural selection as suggested by Girondot and Sire^20^.

From the phylogenetic analysis of recent species in enamel- and toothless clades, with a focus on the phylogenic signal of EMPs, there is clear evidence that amino acid sequence is well preserved from long branch attraction (Fig. 1), and many functional motifs are stable over vertebrate evolution (Supplementary table 1). This implies that there is still a controlling selective pressure over the protein structure protecting some functional motifs from degradation, even though there are neither enamel nor teeth^22,34^. Our current analysis of the conserved sequences shows more regions, where many motifs are stable in Mammalia regardless of the presence of teeth and some are common even for Amniota, Tetrapoda or all evolutionary lineages in our analysis (Fig. 2, Supplementary table 1). One of them forms large areas of docking sites for SH3 domains, which are critical also for the secretory pathway of collagens^35^. The interaction via PDZ proteins is important for the regulation of bone homeostasis^36^ Calcineurin, whose docking sides were detected in several copies in the sequence of AMBN, is one of the major serine-threonine regulatory phosphatases activated by Ca^2+^ and calmodulin and serves as an important differentiation factor in osteoblasts^37,38^. Such domains of the protein will be shaped by different selective pressures than areas strictly connected with protein function in enamel.

The impact of missing EMPs was demonstrated in the wide phenotyping screening results and the detailed changes in bone tissue, where their expression has also been revealed by other authors^39,40^, causing changes in their physical properties. We suggest that it is connected with imbalanced bone homeostasis^22,39,41^, probably by affecting the function of osteoblasts or osteoclasts. It was proven, that e.g. AMBN influences expression of important osteogenesis factors like BMP7 and IGF1^39^ as well as Ca^2+^ oscillation^41^, and Leucine-rich Amelogenin Peptide, a splicing isoform of AMELX, influences Wnt signaling pathway in osteogenesis^42,43^. All these changes can lead primarily to lower porosity and to the other changes observed (Fig. 4, and Supplementary Fig. 7 and 6), although the proper mechanism between the expression and resulting bone phenotype needs to be revealed.

The stronger phenotype in males (Fig. 5b) may correspond to the effect of sex steroids on bone tissue metabolism^44–46^. Although it was reported^39^ that the risk of bone fracture drops with age in male mutant mice, they are more susceptible to serious injury before they can attempt reproduction. Such strict selection is prompt to remove mutations affecting bone structure from the population in time. As there is much wider impact on the animals’ phenotype in mutant mice (Fig. 3), the impact on the fitness of mutants would be even greater under natural conditions, when we take also the other roles of EMPs into account, besides the bone phenotype presented here^32,47^ including possible influence on fertility in *Amelx/Amely* pseudoautosomal complex^48^. Understanding the roles of EMPs in non-canonical physiology systems and which functional motifs are involved can be accessed be precise gene editing and robust phenotyping experiments (similar to our previous work^13^). We can assume that connection between evolutionary history and functional similarities of EMPs can provide certain physiological constrains in non-canonical regulatory functions. The more diversified EMPs might be, the more they are prone to adoption of new functions or rather into specialization to particular physiology regulatory networks. Only functional genomic and phenotyping procedures can give us a better view on functional relations to motif conservancy and evolutionary history of EMPs.

Regarding the model of EMPs as structural proteins, we put together 5 important characteristics for revealing specialization of the proteins from more general preadapted functions: (1) diversification of proteins precedes the appearance of canonical tissue (e.g. enamel); (2) they retain strong phylogenetic signal even in species without canonical tissue, which is further supported by (3) existence of stable, functional motifs, which need not be connected with canonical tissue, and (4) they are expressed outside canonical tissue, where they (5) influence other physiological functions with serious impact on animal’s fitness.

## Methods

### List of sequences

Protein sequences were downloaded from the NCBI GenBank database. As there is not the same abundance of sequences in all species, which could influence tree topology^49^, we decided to pair EMPs according to their role and localization in amelogenesis and phylogenic relationships^17,50^: proteins involved in building protein scaffolds (AMELX and AMBN), and proteins playing a role in enamel-dentin attachment (AMTN and ENAM). This division makes sense also from the evolutionary perspective, as we can assume that degradation of enamel microstructure can precede the creation of functional cap of HAp on the top of dentine, not vice versa.

For AMEL and AMBN 115 sequences were collected (with different species for genus *Balaenoptera*, as the other one had a much worse record for AMBN). The species contain latimeria, 2 frogs (genus *Xenopus*), 7 diapsid reptiles (containing 3 lepidosaurids including 1 snake and 4 archosaurids), platypus, 4 marsupials and 100 placentals from all main evolutionary lineages with the exception of Lagomorpha (i.e. armadillo, pangolin, treeshrew, colugo, 5 afrotherians including aardvark, 16 carnivores, 16 cetarthiodactyls including 6 cetaceans, 9 bats, 3 insectivores, 4 perissodactyls, 22 primates, and 21 rodents). Only an X-linked variant of AMEL (AMELX) was downloaded for mammalian species^51^. If more sequences were known for one species, the most complex one was prioritized.

The same rules were applied for AMTN and ENAM pair, where 170 sequences were collected. As there was no Latimeria sequence present for AMTN and ENAM, we decided to use bichir (*Polypterus*) as the most primitive species in our list as it contains many plesiomorphic character stages for both lineages of bony fish (Osteichthyes)^52^. Besides bichir, the species list includes 3 amphibians (*Xenopus* and 2 caecilians), 17 diapsid reptiles (containing 14 lepidosaurids including 7 snakes and 3 archosaurids), 4 marsupials, and 145 placentals from all main lineages with the exception of pandolis (i.e. armadillo, treeshrew, colugo, 6 aftrotherians including aardvark, 26 carnivors, 25 cetartiodactyls including 11 cetaceans, 18 bats, 4 insectivores, 3 lagomorphs, 4 perrissodactyls, 27 primates, and 29 rodents).

The complete species list with reference numbers is shown in Supplementary table 6.

### Phylogenetic tree structure and protein evolution modeling

Unrooted phylogenetic analyses for each protein were performed separately in Mesquite 3.61 software^53^. Multi-alignment of all sequences for each phylogenetic analysis was computed with Clustal 2.1 application^54^ for AMELX and AMBN, or Muscle 3.8.31^55^ for AMBN and ENAM followed by manual correction. Sub-tree pruning and regrafting method was used for computation of 100 most parsimonious trees, from which a majority-rule consensus tree was constructed.

### Mouse models

*Ambn* KO, and *Ambn*^*G/G*^ mutant mice were produced and phenotyped the Czech Center for Phenogenomics of the Institute of Molecular Genetics CAS, v.v.i. (Vestec, Czechia) as described earlier^13^.

Mice with a null-mutation in the *Amelx* gene (*Amelx* KO) were generated on a C57BL/6n background using a CRISPR genome-editing system at the same facility as mentioned above. Specific guide RNAs (gRNAs) recognizing exon 5 of the *Amelx* gene (gRNA1: 5′-AGGCTGAAGGGTGTGACTCG-3′ and gRNA2: 5′- TGCATGGGCTGATGAGACTG-3′) were designed and off-target analyses were performed using the online software CRISPOR Design Tool (http://crispor.tefor.net/). Cas9 protein and gRNAs were used for zygote electroporation using a protocol described elsewhere^56^. The correct genome editing was confirmed by PCR amplification in the founder mice and animals carrying a null allele were chosen for further breeding.

Animals were bred and maintained in respect to housing, nutrition, and care according to the animal welfare rules of the Czech Republic. All experiments were approved by the Institutional Animal Use and Care Committee (approval no. 62–2016, and 115–2016) and were carried out in accordance with the EU legislation.

### Systematic phenotyping of mouse mutants

Cohorts (7 males and 7 females) of all mouse mutants underwent phenotyping according to standardized procedures of the International Mouse Phenotyping Consortium (IMPC). The phenotyping procedure is described at https://www.mousephenotype.org/impress/. In short, a cohort undergoes (with WT mice) a series of tests in the fixed order and in the fixed age starting with a behavioral screen followed by indirect calorimetry, cardiovascular screen, lung screen, metabolism, body composition, skeletal morphology, bone mineral density, hearing, eye morphology and ended by hematology, immunology, biochemistry, and histopathology.

We used also phenotyping results from mutants in EMPs genes, which were produced and analyzed by other IMPC members according to IMPC policy and downloaded from the public IMPC database using API (https://www.mousephenotype.org/help/programmatic-data-access/): amelotin KO (*Amtn* KO), and enamelin KO (*Enam* KO) mice were analyzed by UC Davis, (California, USA).

### Gene expression

Calvaria and femur bones were isolated and kept in RNA later (Thermofisher Scientific, USA) for 24–48 h. Tissues were mechanically ground, RNA was isolated using RNeasy mini kit (Qiagen, Germany) and used as a template for reverse transcription into cDNA with M-MLV Reverse Transcriptase (Promega, USA) using random primers (Promega, USA). Quantitative PCR (qPCR) reactions were performed using the Light Cycler® 480 SYBR® Green I Master (Roche, Germany) in Light Cycler® 480 Instrument II (Roche, Germany). The expression levels of the genes of interest were normalized to the levels of Rpl19 (F: 5′-aagcctgtgactgtccattc-3′, R: 5′-gatcctcatccttctcatccag-3′). Primers were designed and ordered from Sigma (*Ambn* F: 5′-ccaggttgttgaggaaatgc-3′, R: 5′-cacagtgaatgtcagcatctaag-3′; *Amelx* F: 5′-gcatacactcaaagaaccatcaag-3′, R: 5′-cacctcatagcttaagttgatataacc-3′). All experiments were performed independently in triplicates on 5 WT specimens.

### Immunohistochemistry

Mice were euthanized by cervical dislocation and heart perfused with 4% paraformaldehyde in PBS (pH 7.4). Bones were immediately extracted and fixed in 4% paraformaldehyde at 4 °C for 48 h followed by decalcification in Osteosoft (Sigma-Aldrich, USA) for 14 days. The decalcified bone tissues were embedded in paraffin using a Leica EG 1150H paraffin embedding station (Leica Microsystems, Germany) and cut into 5 μm thick sections, deparaffinized, and rehydrated.

For immunostaining, the deparaffinized and rehydrated sections were briefly washed with PBS. This step was followed by incubation for 30 min in 3% H_2_O_2_ (ThermoFisher Scientific, USA). Then, antigen retrieval was performed for 5 min in the pressure cooker with HIER Citrate Buffer pH 6.0 (Zytomed, Germany), the sections were blocked by 10% normal goat serum (ThermoFisher Scientific, USA) for 1 h at room temperature, and incubated overnight with primary antibodies against AMBN (diluted 1:100, AF3026, RandD Systems, USA) or AMELX (diluted 1:200, produced in Wald et al., 2017). After washing with PBS, slides were incubated with the secondary antibody ZytoChem Plus HRP Polymer anti-Mouse (Zytomed, Germany) for 30 min followed by 3,39-diaminobenzidine (DAB) development using DAB Substrate Kit (Zytomed, Germany). Hematoxylin was used for counterstain. The sections were dehydrated, mounted with coverslips, and examined using Zeiss Axioscan Z1 (Carl Zeiss AG).

### Bone structural analysis

10 mice (5 males and 5 females) from all genetic lines, i.e. wild types (WT), *Ambn* KO, *Ambn*^*G/G*^, and *Amelx* KO, were sacrificed by cervical dislocation at the age of 11–15 weeks.

Their femurs and L4s were banded into 2.5% low melting agarose (Sigma-Aldrich Co., USA). After stabilization in the fridge (4 °C) for at least 24 h, they were scanned in Bruker SkyScan 1272 High-Resolution X-Ray Microtomograph (Bruker, Belgium) in the resolution of 1.5 µm per voxel (1 mm Al filter, voltage 80 kV, current 125 µA, exposure 2584 ms, rotation 0.21° in 360° scan, 2 × averaging).

NRecon 1.7.3.1 (Bruker, Belgium) with InstaRecon 2.0.4.0 (InstaRecon, USA) engine were used for reconstructions. Reconstruction parameters were set up to smoothing = 6, ring artifact reduction = 8, beam hardening correction = 28%, and defect pixel masking threshold = 10%. The range of intensities was set up from 0.00 AU to 0.110 AU in femurs and to 0.125 AU for the 4th lumbar vertebrae (L4).

Reconstructions were reoriented to the same position using DataViewer 1.5.4.0 (Bruker, Belgium) and subsequently regions of interest were chosen in CT Analyser 1.18.4.0 (Bruker, Belgium)—central and distal part of femur, and distal part of L4 body. We also used CT Analyser for the final analysis of cortical (central femur and L4) and trabecular bone (distal femur and L4)—see Supplementary Fig. 6 for localization of regions of interest. Bone was separated from the background by the Otsu method^57^. Region of interest for trabecular bone was selected automatically based on the modified Bruker Method note MCT-124 (Supplementary table 7) and checked manually. Bone volume, porosity, and TMD in cortical bone in addition to relative bone volume, volume to surface ratio, number of objects per slice, object distance, and thickness in trabecular bone were identified. TMD was established with calibrated HAp phantoms (25 and 75%) scanned and reconstructed under the same conditions as samples. We used CTvox 3.3.0 (Bruker, Belgium) for visualization.

### Bone fracture analysis

The same femurs as above were tested. The only exception are *Amelx* KO males, where only 3 specimens were available at the time. Femur bone and lumbar spine were dissected post mortem. The femurs underwent bone fracture analysis by 3-point bend test fixture 550lb G238 from TestResources (Shakopee, USA) with the upper roller and lower supports having a diameter of 2 mm. The tests were run on a Zwick tensile test machine (Zwicki, ZwickRoell, Germany) with a 200 N load cell and the following test parameters: distance of lower supports = 10 mm; pre-load = 0.1 N; speed until pre-load = 50 mm/min; test speed = 10 mm/min. The value measured was Force at break (F). The bones were stored at room temperature before testing. The samples were placed in a similar manner on the supports with the distal end to the right and the proximal side to the left. The posterior surface was facing down. The femurs were detached from the remains of the tibia prior to testing and any remaining soft tissue was removed immediately before testing. Values for Force at break were excluded, when the specimen slipped from the machine.

### Femur morphometry

Femurs were measured in 3D from whole body scans in DataViewer 1.5.4.0 (Bruker, Belgium).

For data acquisition, anesthetized (20% zoletil + xylazim—Virbac, France) mice were scanned in Bruker SkyScan 1176 High Resolution In-Vivo X-Ray Microtomograph (Bruker, Belgium) at the resolution of 35 µm (0.5 mm Al filter, voltage 50 kV, current 160 µA, exposure 200 ms, rotation 0.7° in 180° scan). NRecon 1.7.1.0 (Bruker, Belgium) was used for reconstructions with parameters set up to smoothing = 3, ring artifact reduction = 4, and beam hardening correction = 36%. The range of intensities was set up from 0.0047 AU to 0.1230 AU.

15 *Ambn* KO (7 females, 8 males), 15 *Ambn*^G/G^ (8 females, 7 males), and 15 *Amelx* KO (7 females and 8 males) mice were used for the analysis together with their WT cohort counterparts, 11 females and 19 males.

### Statistical analysis

All experiments and analysis were provided in a way to match criteria defined by ARRIVE guidelines 2.0^58^.

All data were tested using R 3.6.2 software^59^ with the support of RStudio 1.2.5033^60^ as linear mixed-effect models for most variables, for categorical variables of the systematic cohort phenotyping, Fisher test was used instead. For standardized mouse cohorts, the cohort was used as a covariable to prevent inter-cohort variability. We used libraries tidyverse^61^, lme4^62^, multcomp^63^, and gridExtra^64^ for computation and graphical outcomes of the analysis.

Results of the systematic phenotyping analysis were visualized in the form of a circular graph, where each radius represents one variable scored by the facility, and the length of the line corresponds to the reverse *p* value. *p* values higher than 0.95 were taken as 0.95 to visualize all of them. Variables were split to eight groups described as Sensory, Morphology, Cardiovascular, Metabolism, Immunology, Behavioral, Reproduction, and Respiratory. Variables are ordered by increasing *p* value (decreasing significance) within each group.

Graphs for expression data were generated using GraphPad Prism v8.1.2 (San Diego, California USA).

## Supplementary Information

Supplementary Information 1.

Supplementary Information 2.

Supplementary Information 3.

Supplementary Information 4.

Supplementary Information 5.

Supplementary Information 6.

Supplementary Information 7.

Supplementary Information 8.

Supplementary Information 9.

Supplementary Information 10.

Supplementary Information 11.

Supplementary Information 12.

Supplementary Information 13.

Supplementary Information 14.

Supplementary Information 15.

Supplementary Information 16.

Supplementary Information 17.

Supplementary Information 18.

Supplementary Information 19.

Supplementary Information 20.

## Data Availability

All relevant data, which served for creation of graphs and statistical analysis are included in the supplementary tables, namely: results of IMPC phenotyping in the Supplementary table 2, data for bone ultrastructure and bone strength analysis in the Supplementary table 5, and data for femur and body length in the Supplementary table 6. Data from IMPC phenotyping are or will be accessible at the IMPC website https://www.mousephenotype.org/data/genes/MGI:88005; https://www.mousephenotype.org/data/genes/MGI:104655; https://www.mousephenotype.org/data/genes/MGI:1918671; https://www.mousephenotype.org/data/genes/MGI:1333772; Ambn and AmelX expanded data are in supplementary material “Spoutil et al., CCPspecphenotyping reports.zip”. List of protein sequences with their reference numbers is available in the Supplementary table 7 and aligned protein sequences, which were directly used in the phylogenetic analysis, are present in FASTA format in the supplementary file FASTA-EMPs.zip. CT analyzer task list for trabecular ROI selection is described in the Supplementary table 8 and all task lists used for microCT analysis are available in the supplementary file CTanTaskLists-BoneUltrastructure.zip. MicroCT data (raw Xray images as well as reconstructions) will be provided upon a reasonable request. Please, contact Jan Prochazka in that matter.
