# Supplementary material for: Early evolution of enamel matrix proteins is reflected by pleiotropy of physiological functions
Source: Sci Rep. 2023 Jan 26;13:1471. doi: 10.1038/s41598-023-28388-4 (PMC9879986; doi:10.1038/s41598-023-28388-4)
Supplement: Supplementary file 4 — Supplementary Information 4. [file 41598_2023_28388_MOESM4_ESM.docx]

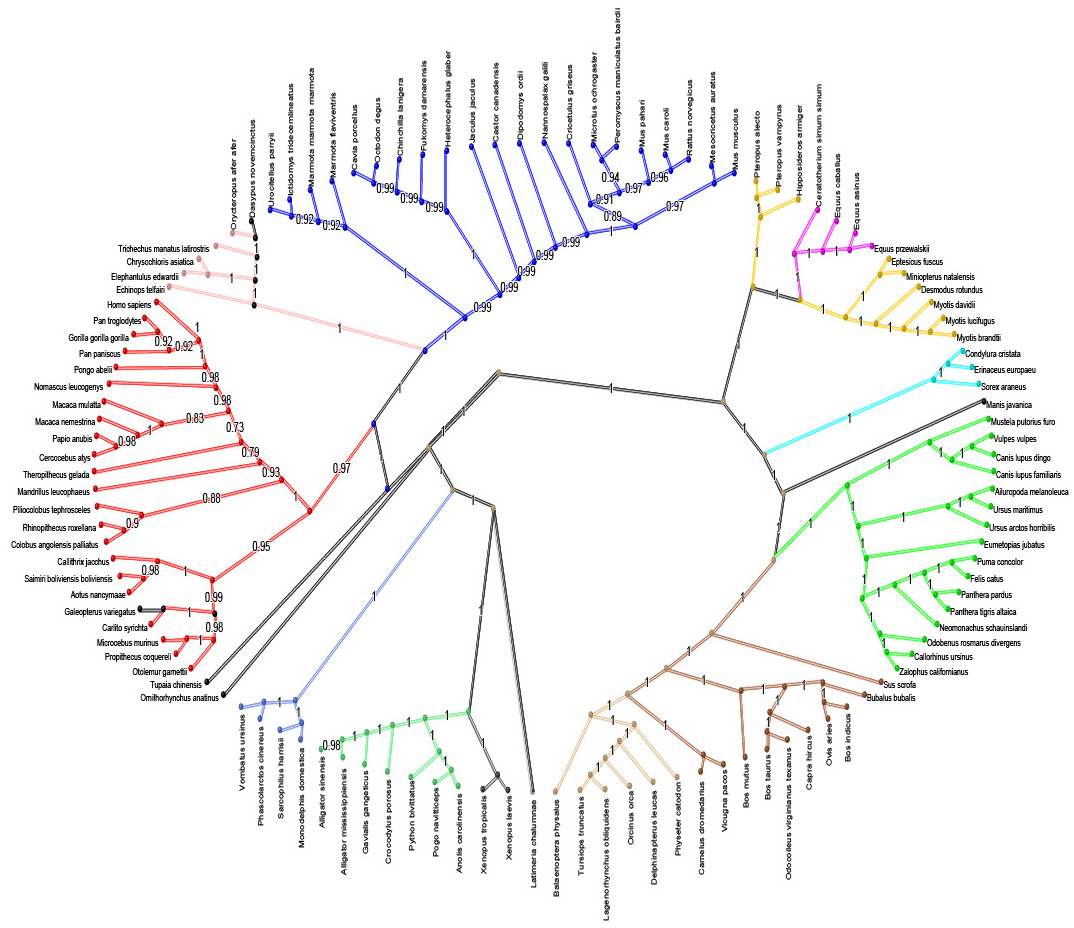


**Supplementary Figure 1.** Majority consensus phylogenetic trees of amelogenin protein sequences.

Colors correspond to taxonomic clades: Sarcopterygii (light grey), Anura (dark grey), Diapsida (green), Marsupialia (sea blue), Afrotheria (pink), Rodentia (blue), Primates (red), Carnivora (light green), Chiroptera (yellow), Eulipotyphla (light blue), Perissodactyla (violet), “Artiodactyla“ (brown), Cetacea (light brown). Number shows portion of 100 most parsimonous trees, in which was the node stable.


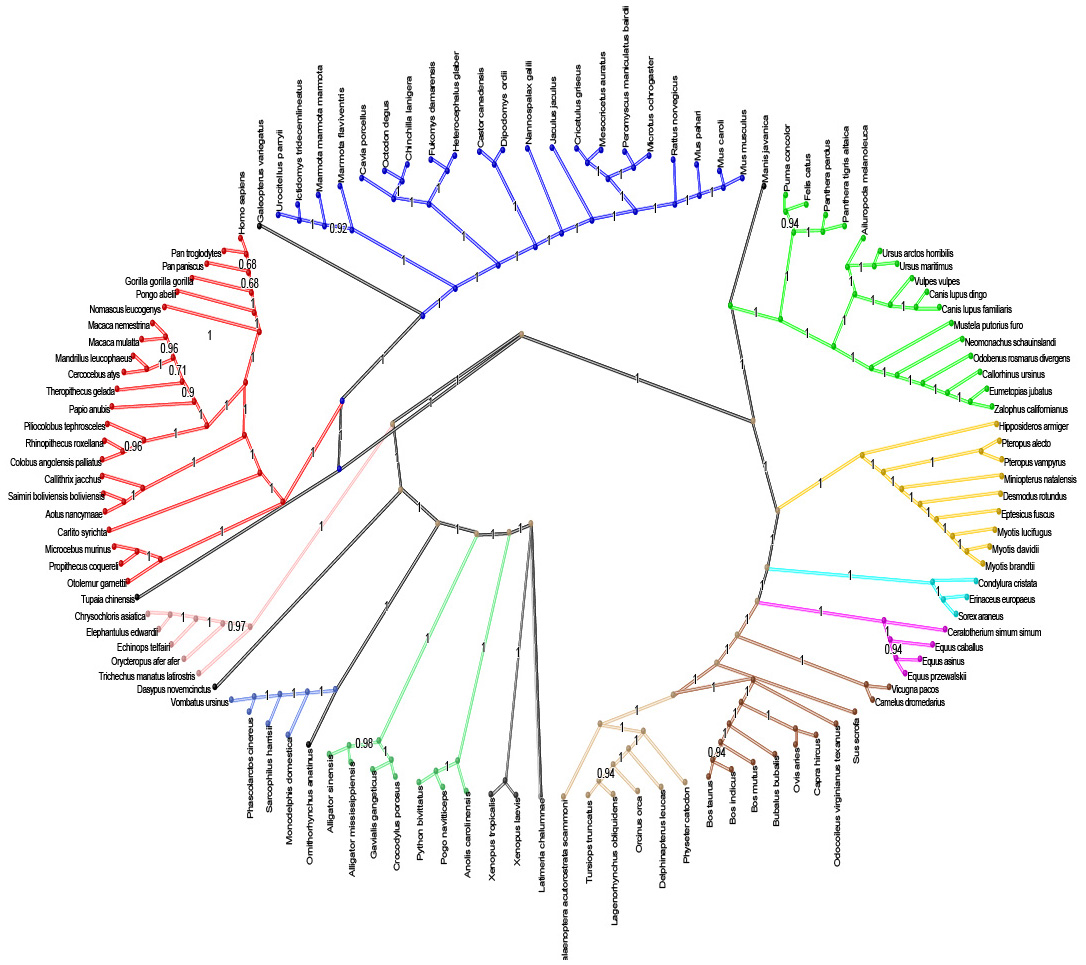


**Supplementary Figure 2.** Majority consensus phylogenetic trees of ameloblastin protein sequences.

Colors correspond to taxonomic clades: Sarcopterygii (light grey), Anura (dark grey), Diapsida (green), Marsupialia (sea blue), Afrotheria (pink), Rodentia (blue), Primates (red), Carnivora (light green), Chiroptera (yellow), Eulipotyphla (light blue), Perissodactyla (violet), “Artiodactyla“ (brown), Cetacea (light brown). Number shows portion of 100 most parsimonous trees, in which was the node stable.


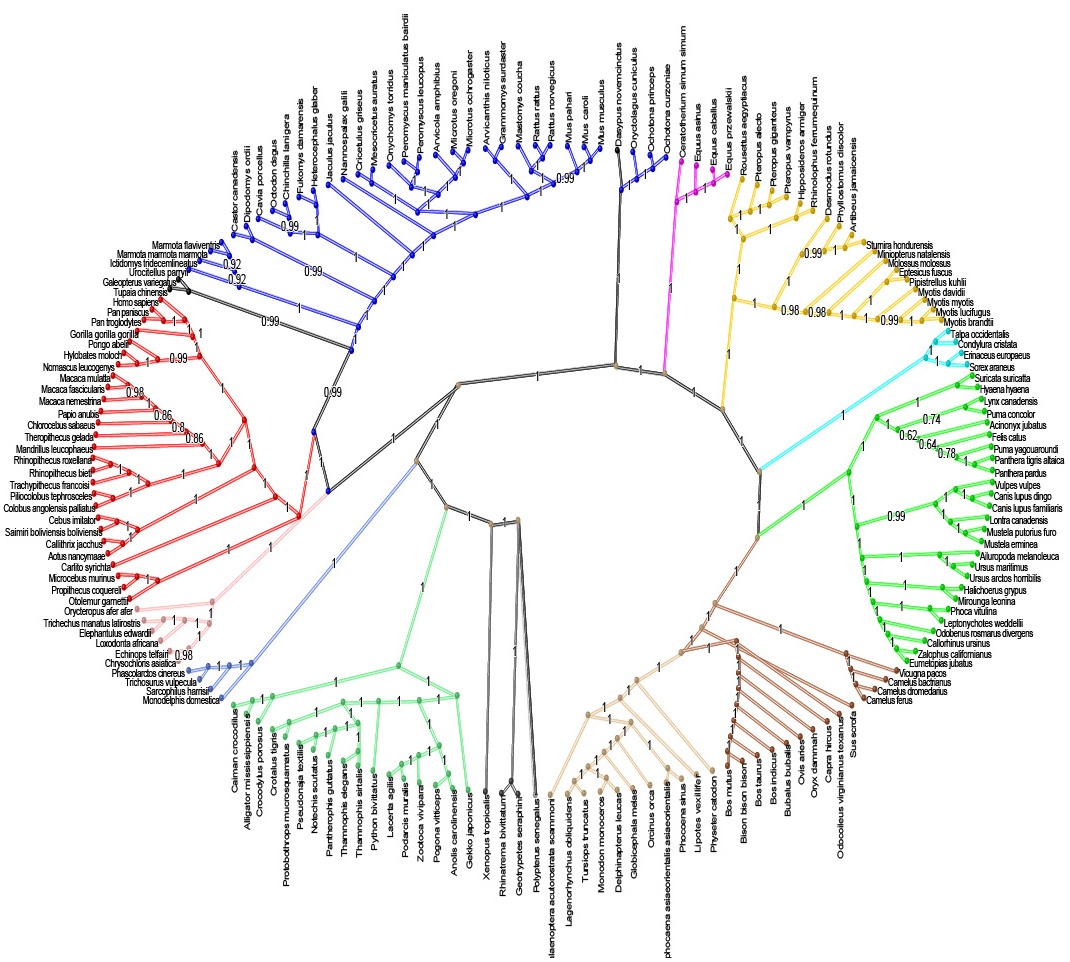


**Supplementary Figure 3.** Majority consensus phylogenetic trees of amelotin protein sequences.

Colors correspond to taxonomic clades: Polypteriformes (light grey), Amphibia (dark grey), Diapsida (green), Marsupialia (sea blue), Afrotheria (pink), Glyres (blue), Primates (red), Carnivora (light green), Chiroptera (yellow), Eulipotyphla (light blue), Perissodactyla (violet), “Artiodactyla“ (brown), Cetacea (light brown). Number shows portion of 100 most parsimonous trees, in which was the node stable.


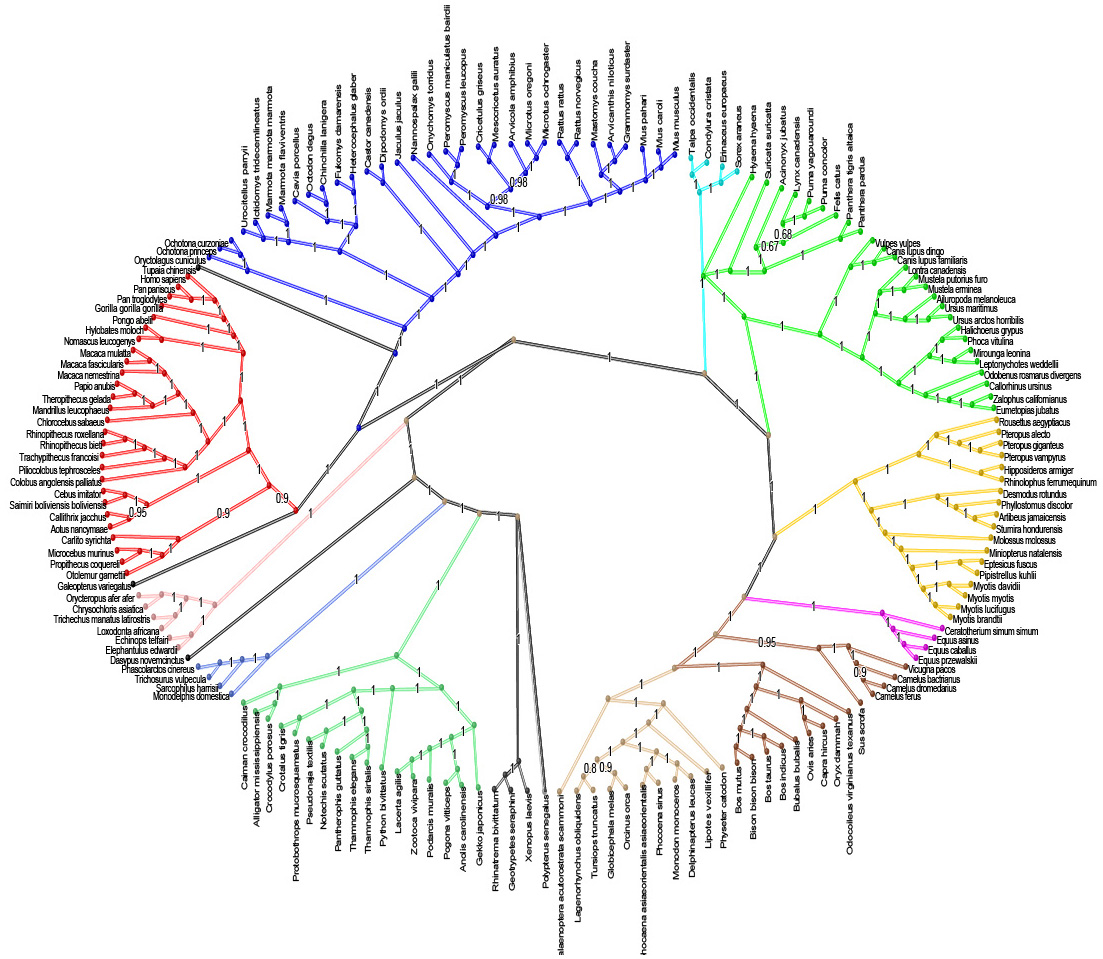


**Supplementary Figure 4.** Majority consensus phylogenetic trees of enamelin protein sequences.

Colors correspond to taxonomic clades: Polypteriformes (light grey), Amphibia (dark grey), Diapsida (green), Marsupialia (sea blue), Afrotheria (pink), Glyres (blue), Primates (red), Carnivora (light green), Chiroptera (yellow), Eulipotyphla (light blue), Perissodactyla (violet), “Artiodactyla“ (brown), Cetacea (light brown). Number shows portion of 100 most parsimonous trees, in which was the node stable.

**Supplementary Figure 5.** Expression of *Ambn* and *Amelx* in bone tissue of mouse.


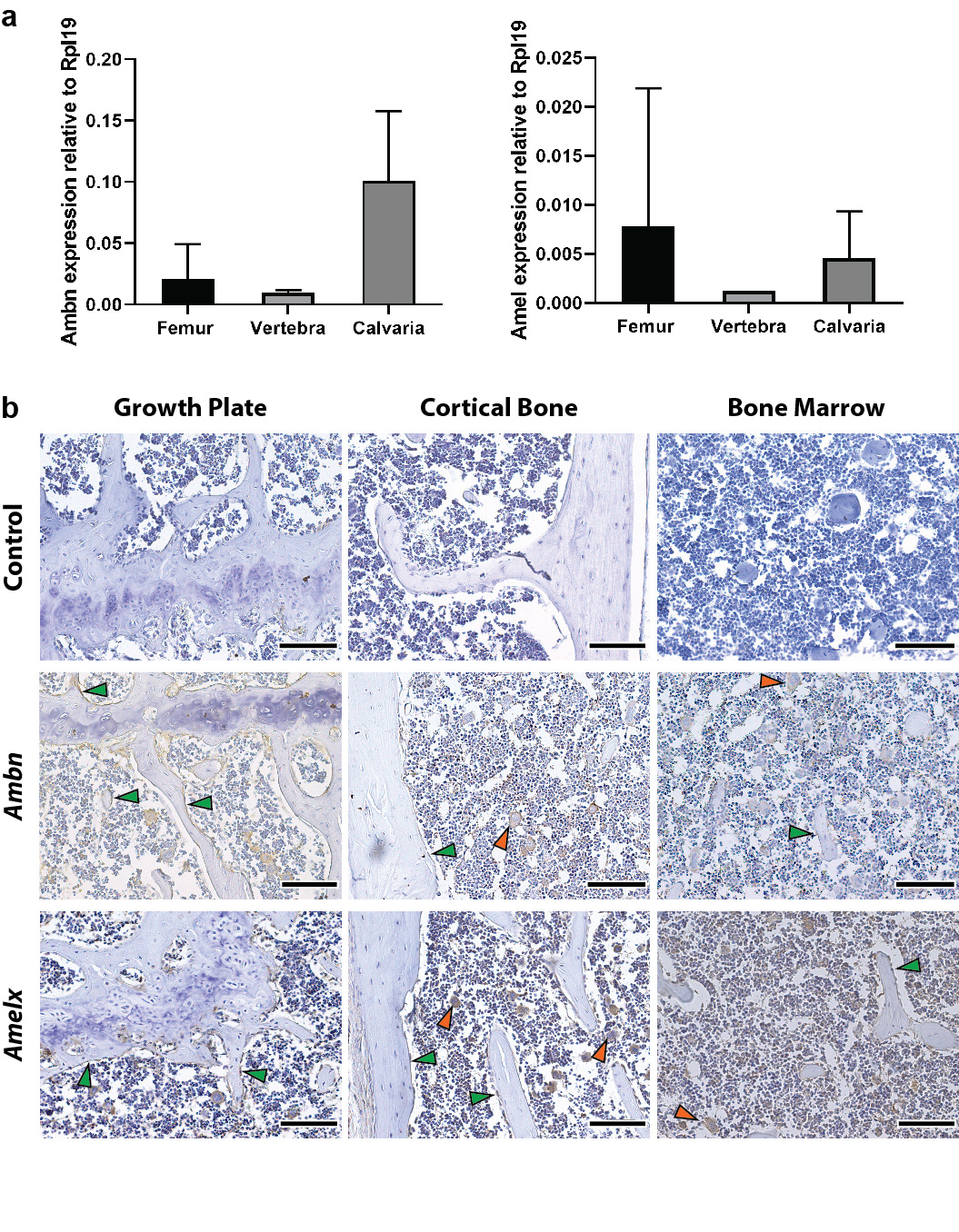


(**a**) Quantification of *Ambn* and *Amelx* expression in femur, vertebra, and calvaria. Expression relative to Rpl19. Y-axis have different scaling. (**b**) Lac-Z stained representative images of *Ambn* and *Amelx* expression in grow plate, cortical bone, and bone marrow of mouse. Expression signal in brown (DAB staining), green arrowheads mark examples of osteoblasts, orange arrowheads of megakaryocytes. Bar = 100 µm.


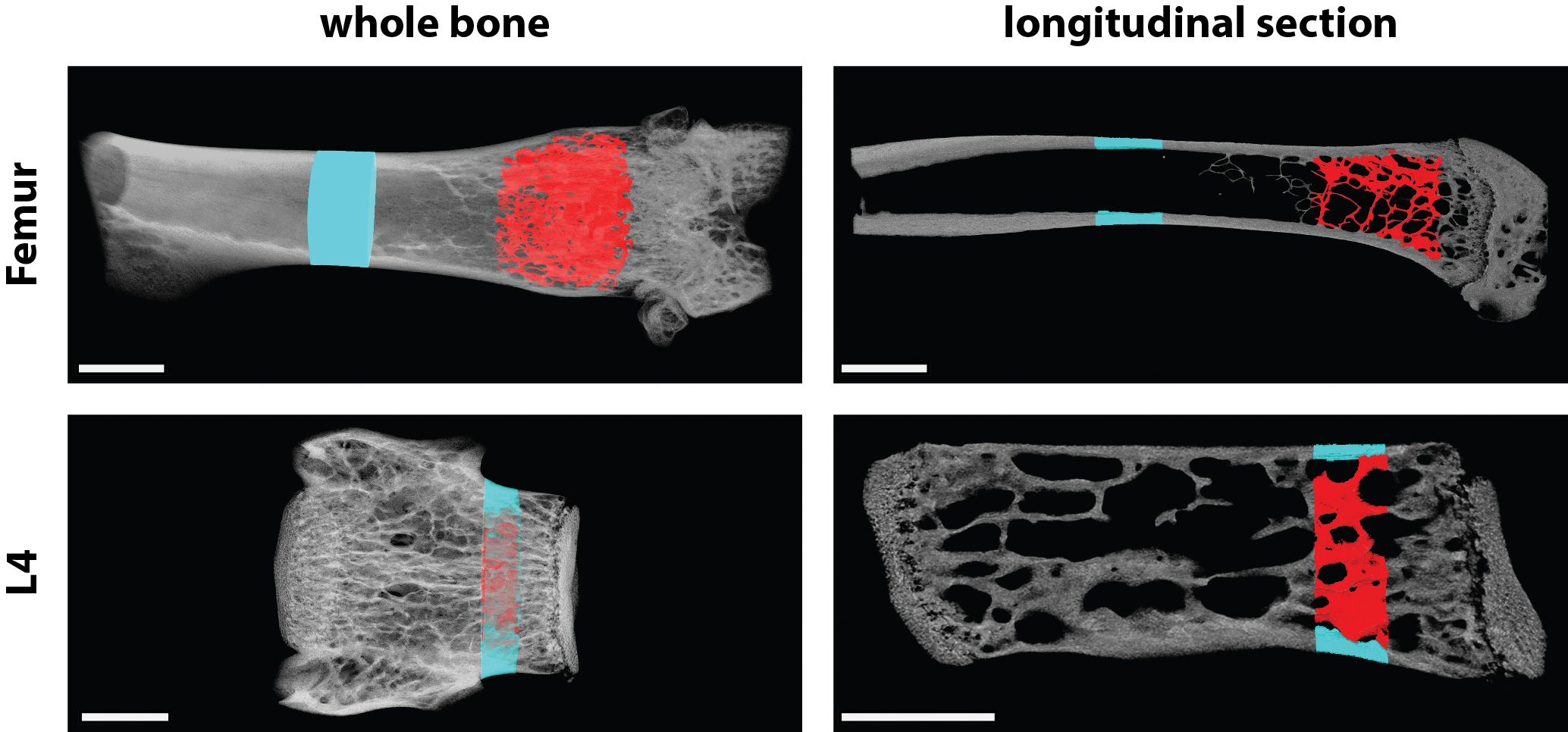


**Supplementary Figure 6.** Volumes of interests for bone analysis.

Femur *(****top****)* and L4 vertebra *(****bottom****)* - whole volume *(****left****)* and longitudinal section *(****right****)*. Trabeculi in red, cortical bone in light blue. Distal side on right. Bar = 1 mm.


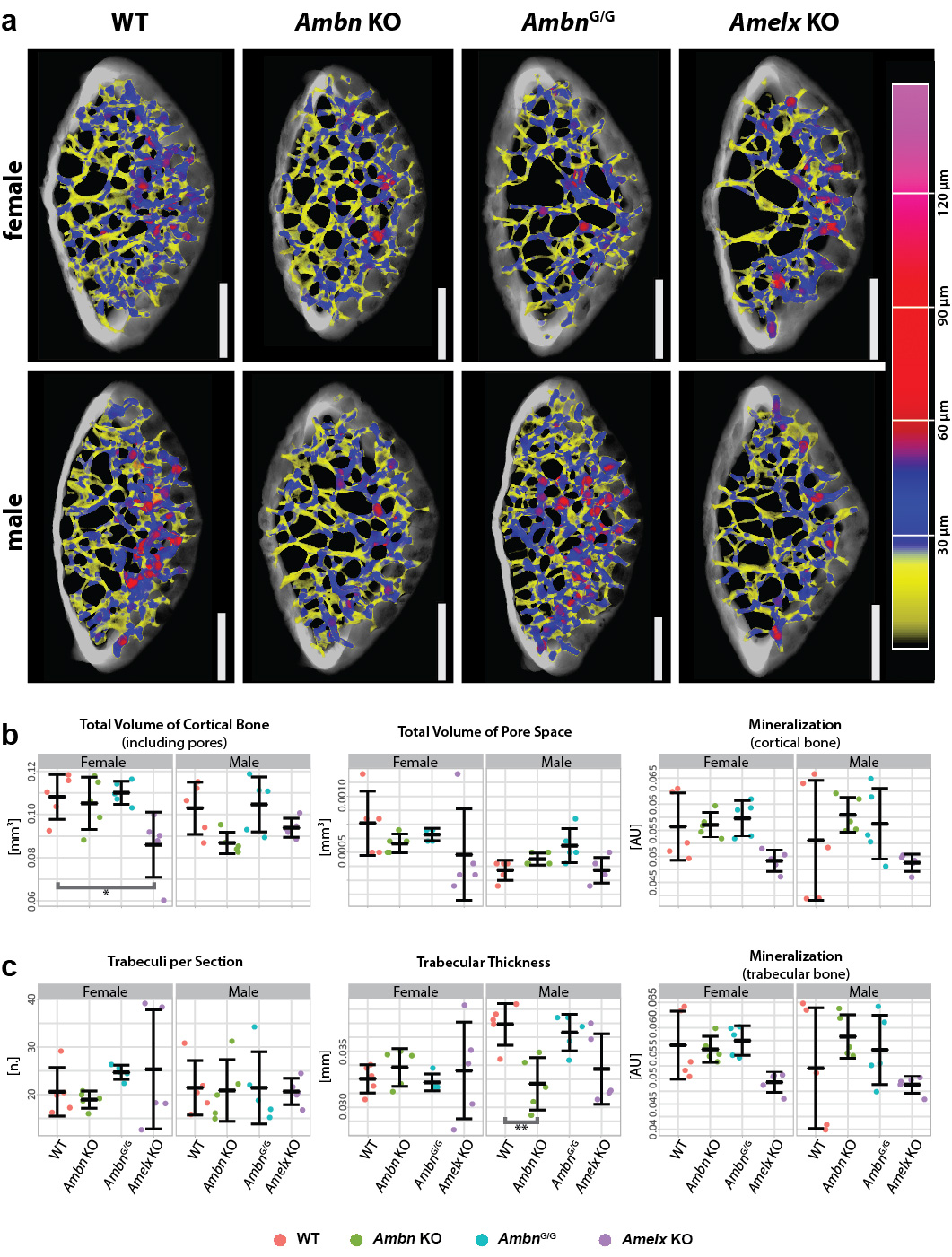


**Supplementary Figure 7.** L4 bone structure.

(**a**) Trabecular structure analyzed by microCT. Pseudocolors correspond to trabecular thickness (spectrum on the right). Specimens closest to group’s average selected. Bar = 500 µm. (**b**) Whisker plots of L4 cortical bone microstructure: total volume of cortical bone (including pores), total volume of pore space, both in mm3, and mineralization of cortical bone in Attenuation units (AU). (**c**) Whisker plot of L4 trabecular bone: mean number of trabeculi per section, the mean thickness of trabeculi in mm, and mineralization of trabecular bone in AU. Females on the left and males on the right of each graph. Thick midline = mean, whiskers = standard deviation; * < 0.05, ** < 0.01. Values for each observation are shown as points – WTs in red, *Ambn* KO in green, *Ambn^G/G^* in blue, and *Amelx* KO in violet.


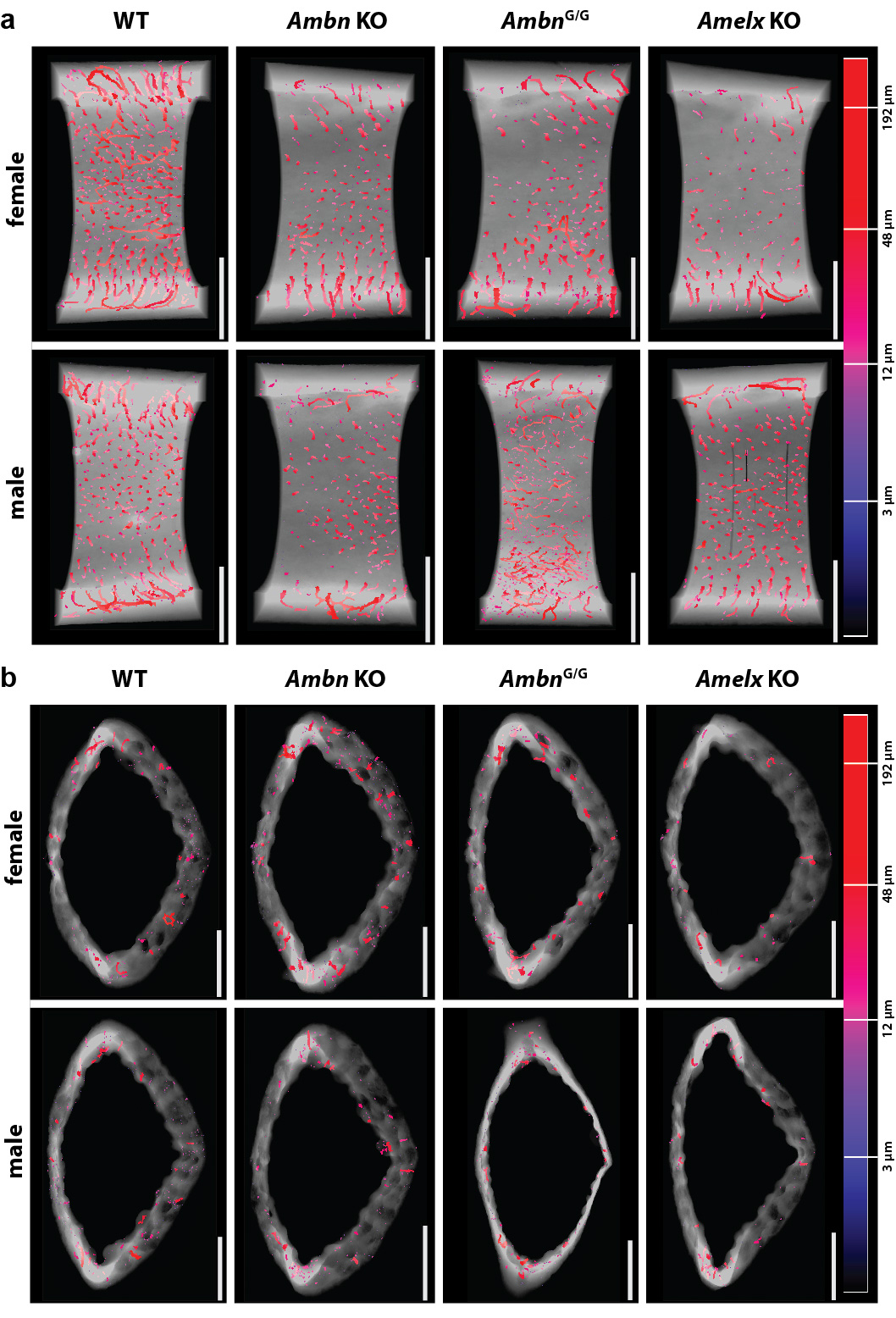


**Supplementary Figure 8.** Porosity of cortical bone.

Central femur (**a**) and caudal body of L4 (**b**). Pseudocolors correspond to pore size recalculated to volume equivalent sphere diameter (spectrum on the right). Specimens closest to group’s average selected. Bar = 500 µm.

**Supplementary table 1.** List of conservative motifs found in EMPs. Position and form of motifs correspond to those find in sequences of EMPs in *Mus musculus:* BAA06546.1 for AMELX, AAB93765.1 for AMBN, NP_082069.1for AMTN, and AAB94312.1 for ENAM. Motifs are ordered by their appearance. * Corresponds to position and structure in *Mus musculus*. ** mentioned series of the same motifs overlapping or in a distance not greater then a motif length was considered as a cluster, the number corresponds to maximal number of motifs in cluster in all lineages. *** List of monophyletic, taxonomical groups, where the motif is missing, exceptions are after “BUT”, if taxonomical distribution of a motif in such group is not clear, “most” or “some” is before the name of the group depending on how great is the absence of a motif. Evolutionary lineages represented by just one genus are mentioned by the genus name. Species without teeth or enamel are excluded from the list and have their special column: N.A. stands for “non-appropriate,” as we either do not possess the sequence, or the motif is not relevant for that taxonomical level (e.g. in motifs common for Euarchontoglyres).

**Supplementary table 2.** Overview of statistical results for individual genes from IMPC phenotyping procedure.

Color of the field highlights the level of significance from highly significant results (p < 0.05) in red to non-significant results in blue. N.A. = not tested in this cohort.

**Supplementary table 3.** Summary of statistical analysis of bone fracture and bone structure analysis of femur.

Significant results (p < 0.05) in red, results with p < 0.1 in blue.

**Supplementary table 4.** Summary of bone structure analysis of L4.

Significant results (p < 0.05) in red, results with p < 0.1 in blue.

**Supplementary table 5.** Dataset for analysis of bone ultrastructure and bone strength.

**Supplementary table 6.** Dataset for femur morphometry and body length.

**Supplementary table 7.** List of protein sequences used in phylogenetic analysis.

Ambn = ameloblastin, Amel = amelogenin, Amtn = amelotin, Enam = enamelin.

**Supplementary table 8.** Task list for analysis of bone ultrastructure.

Modified task lists for CT Analyser (Bruker, Belgium) for automatic selection of region of interest for trabecular bone analysis. Femur-only related specifications in red, L4-only related in blue.
