# Supplementary figures and images for: Early evolution of enamel matrix proteins is reflected by pleiotropy of physiological functions
Source: Sci Rep. 2023 Jan 26;13:1471. doi: 10.1038/s41598-023-28388-4 (PMC9879986; doi:10.1038/s41598-023-28388-4)

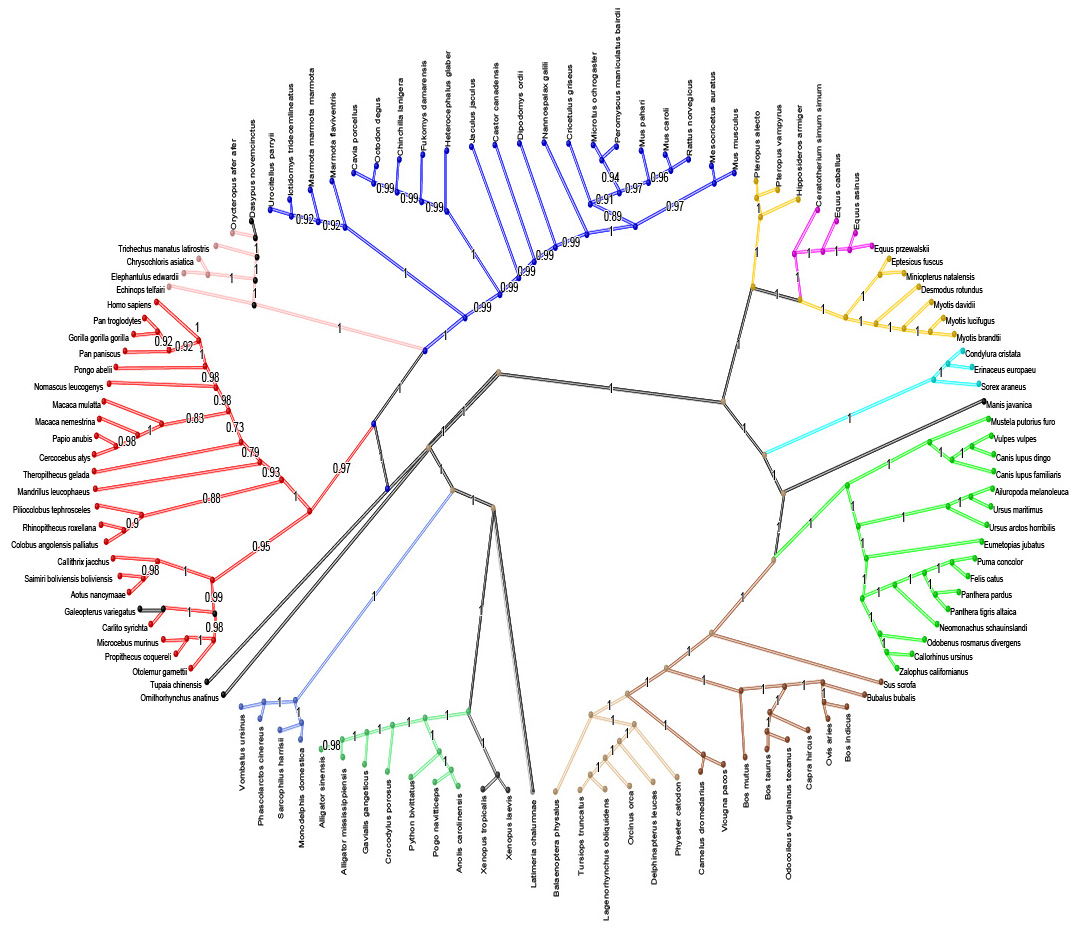

Supplement: Supplementary file 5 — Supplementary Information 5. [file 41598_2023_28388_MOESM5_ESM.jpg]

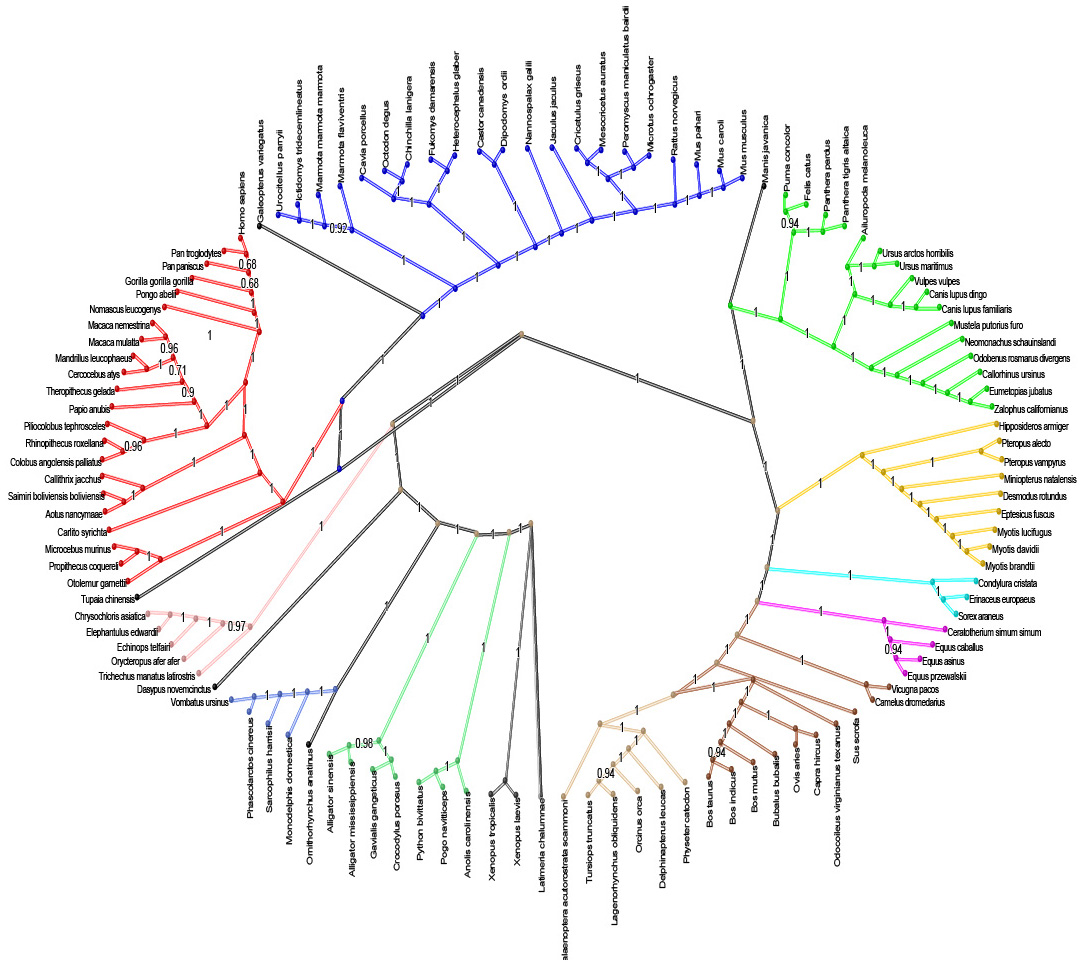

Supplement: Supplementary file 6 — Supplementary Information 6. [file 41598_2023_28388_MOESM6_ESM.jpg]

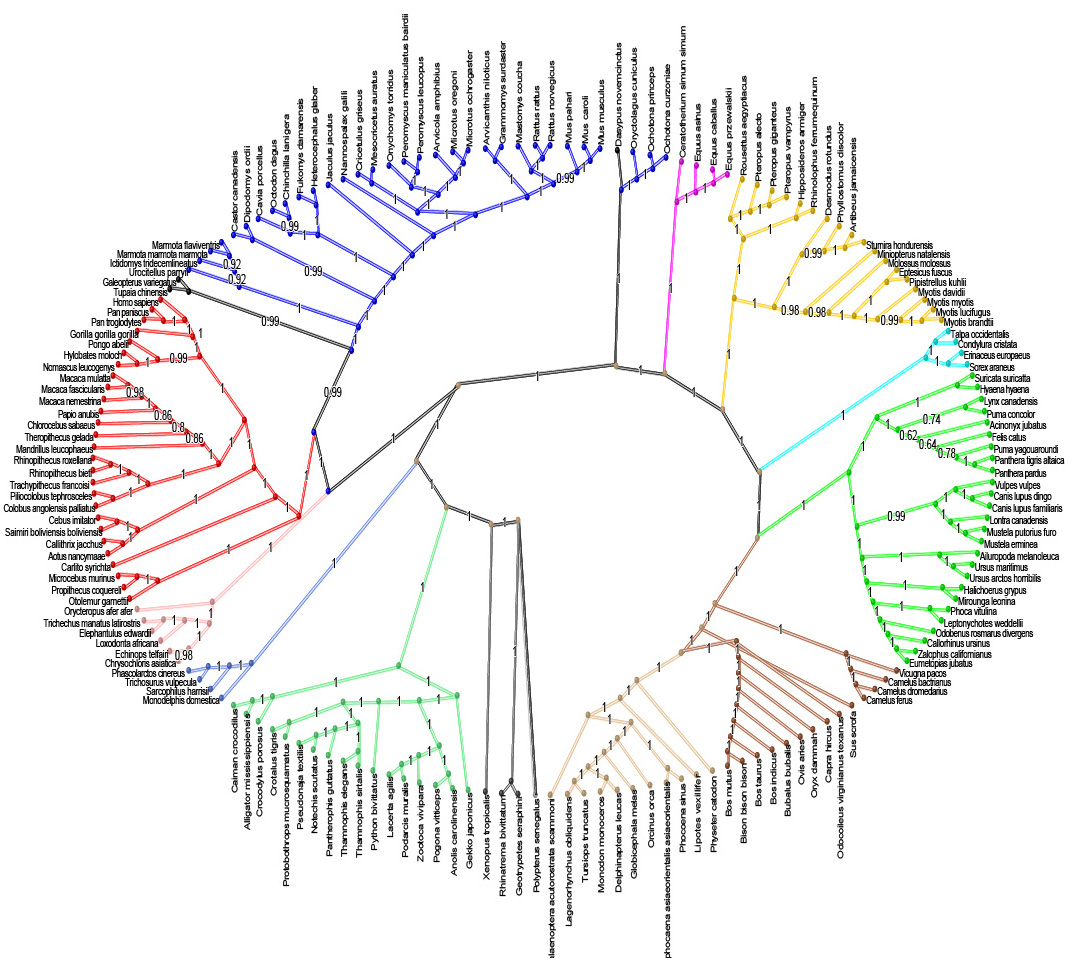

Supplement: Supplementary file 7 — Supplementary Information 7. [file 41598_2023_28388_MOESM7_ESM.jpg]

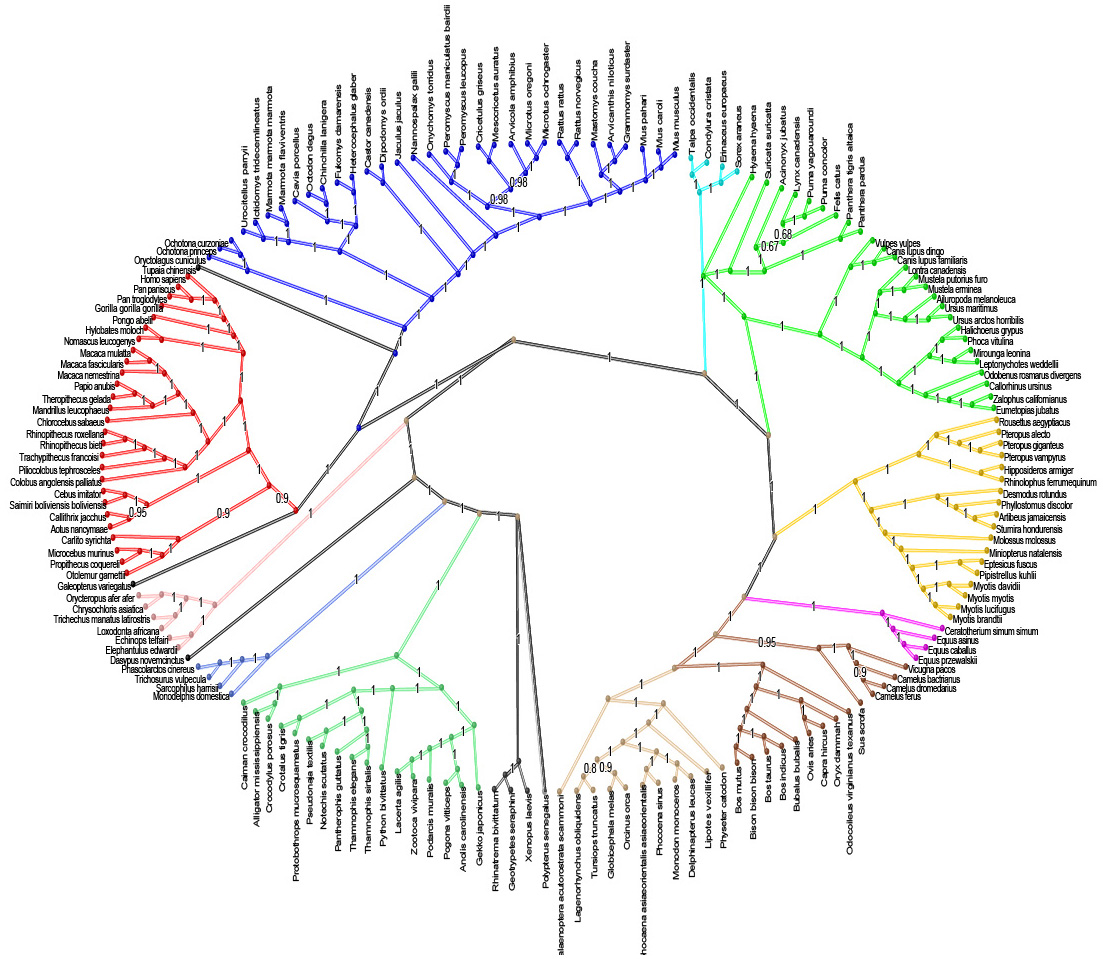

Supplement: Supplementary file 8 — Supplementary Information 8. [file 41598_2023_28388_MOESM8_ESM.jpg]

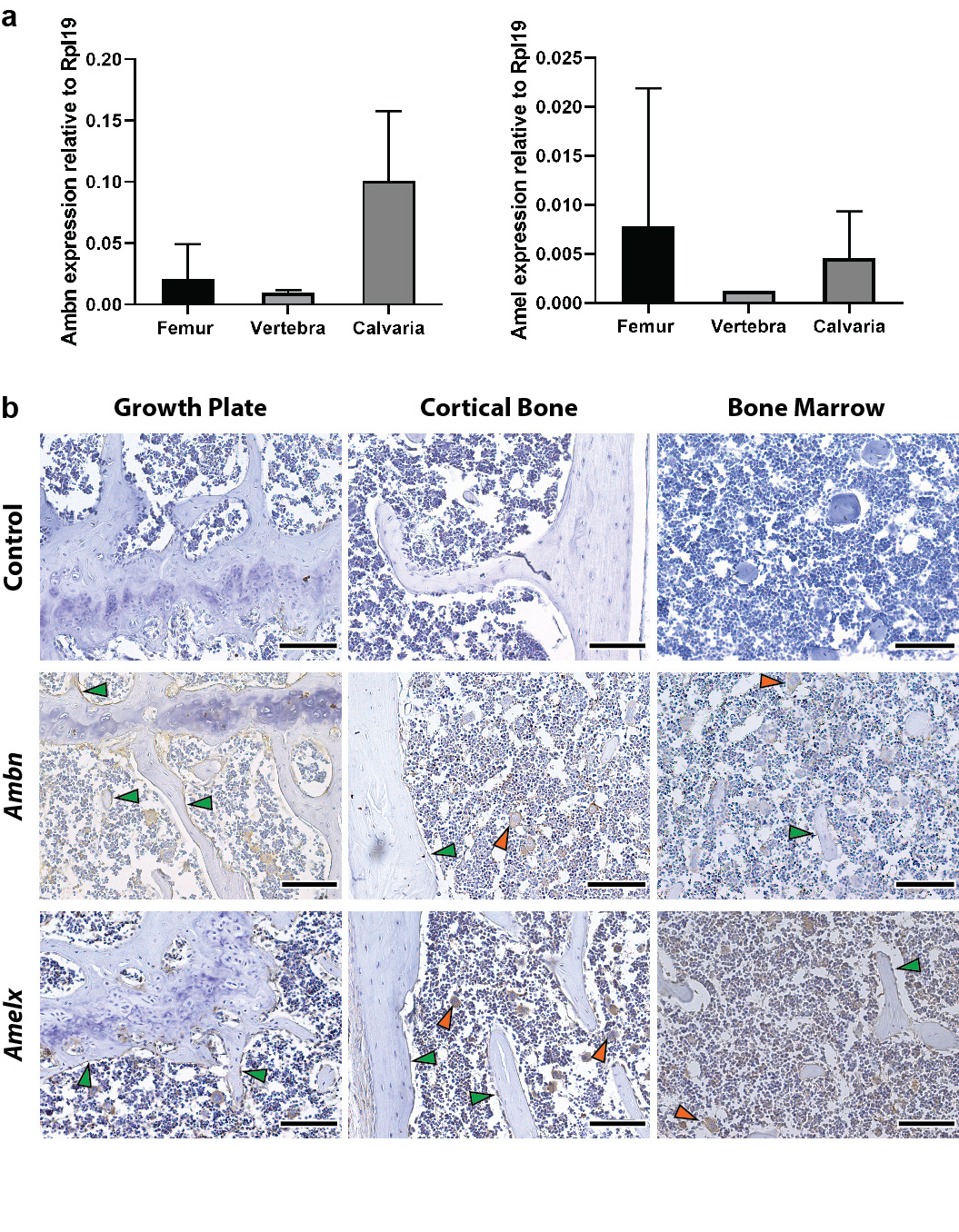

Supplement: Supplementary file 9 — Supplementary Information 9. [file 41598_2023_28388_MOESM9_ESM.jpg]

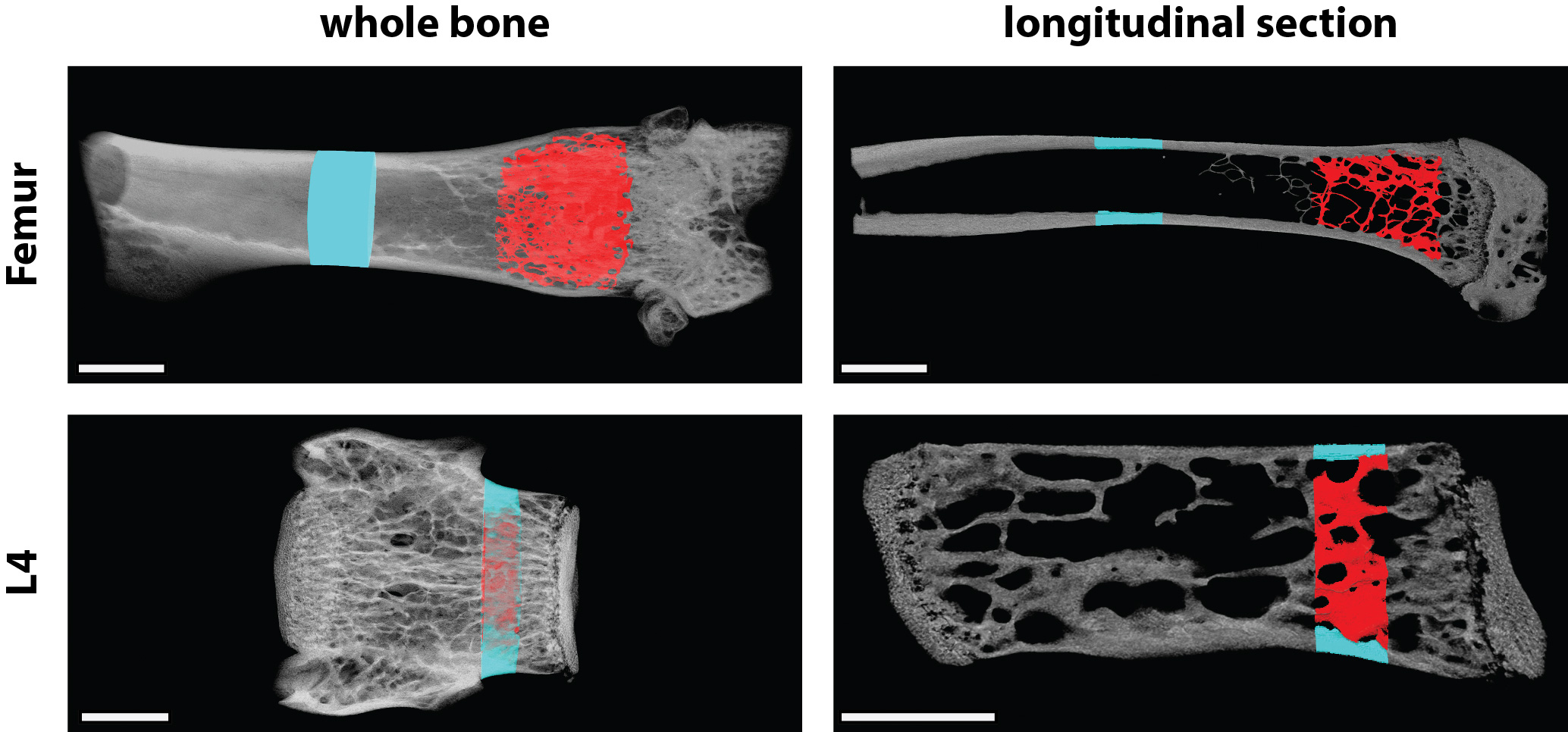

Supplement: Supplementary file 10 — Supplementary Information 10. [file 41598_2023_28388_MOESM10_ESM.jpg]

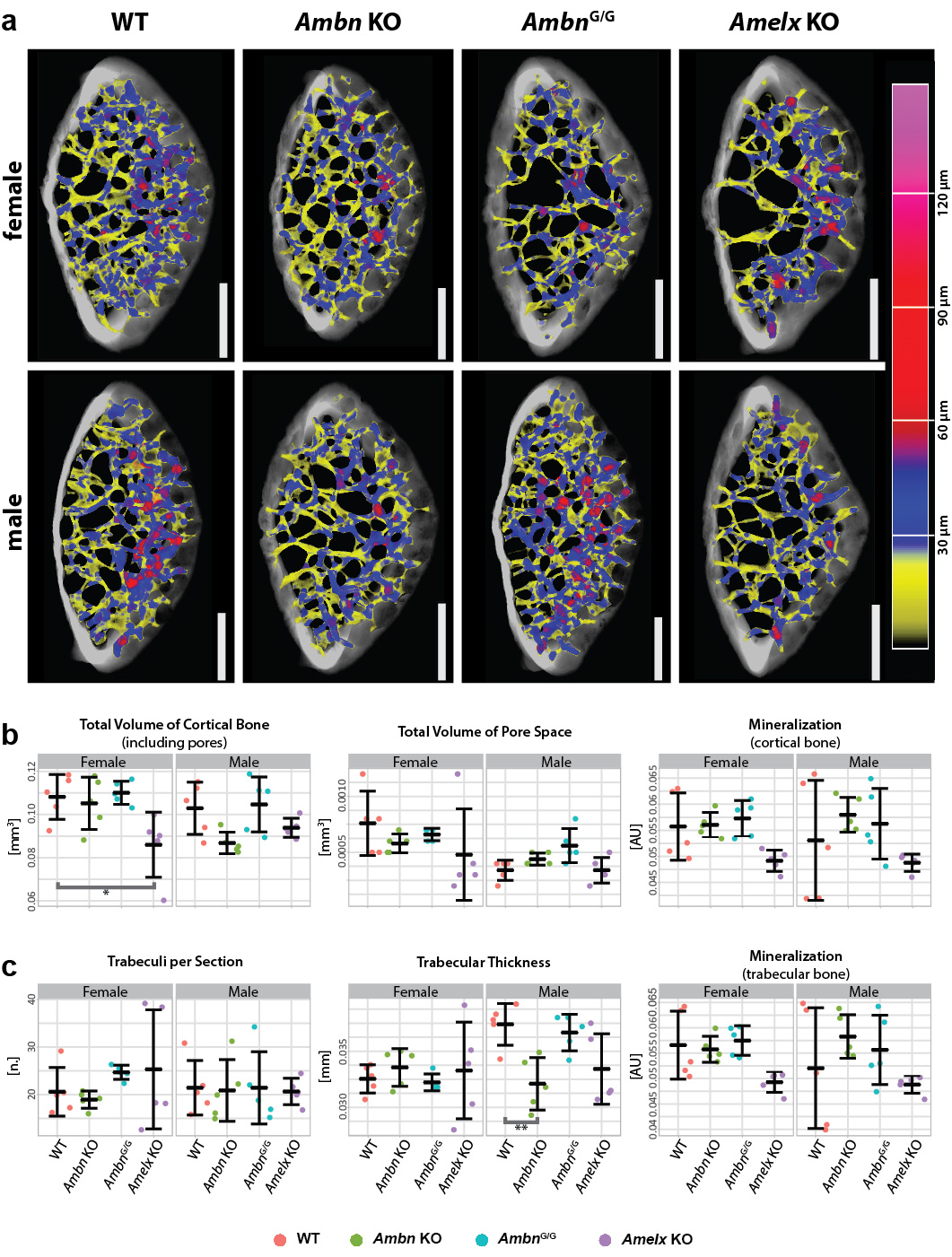

Supplement: Supplementary file 11 — Supplementary Information 11. [file 41598_2023_28388_MOESM11_ESM.jpg]

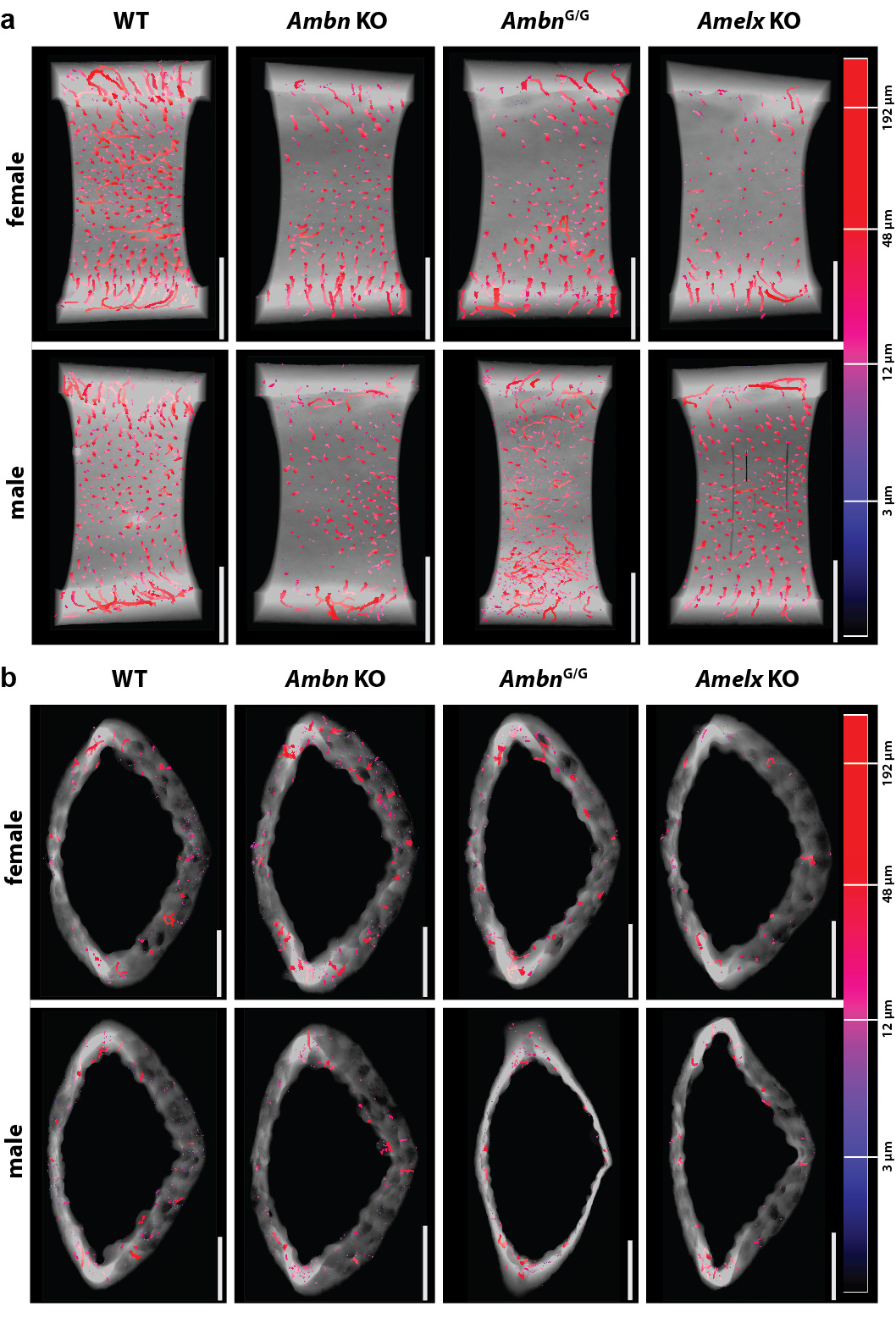

Supplement: Supplementary file 12 — Supplementary Information 12. [file 41598_2023_28388_MOESM12_ESM.jpg]
