# Supplementary material for: Early evolution of enamel matrix proteins is reflected by pleiotropy of physiological functions
Source: Sci Rep. 2023 Jan 26;13:1471. doi: 10.1038/s41598-023-28388-4 (PMC9879986; doi:10.1038/s41598-023-28388-4)
Supplement: Supplementary file 3 — Supplementary Information 3. [file 41598_2023_28388_MOESM3_ESM.zip › Ambn_ggg.html]

Ambn\_ggg


# Ambn\_ggg

The Czech Centre for Phenogenomics (CCP) is a large research infrastructure dedicated to providing; first-class expertise, tools, and services to reveal gene functions in human diseases, identifying new therapeutic targets for treating human diseases and enhancing the understanding of the genetic bases for human diseases. CCP was established in 2015 as part of the wider Biotechnology and Biomedicine Center of the Academy of Sciences and Charles University in Vestec (BIOCEV) project and is hosted by the Institute of Molecular Genetics of the ASCR, v. v. i. Located in the municipality of Vestec, Czech Republic, CCP provides open access, state of the art services for both the national and international research community. As a member of two large consortia (IMPC and INFRAFRONTIER), CCP is committed to discovering and ascribing biological function to each gene through the generation of mutant mouse lines and use of the broad based primary phenotyping pipeline which includes all the major adult organ systems.

**Director** : Radislav Sedlacek, Assoc. Prof., PhD

**Phenotyping Module Head**: Jan Prochazka, PhD

Czech Centre for Phenogenomics, BIOCEV (IMG), Prumyslova 595, 252 50 Vestec, Czech Republic

# 1 Introduction

## 1.1 Overview of gene function

[1] “Official Symbol: Ambn (provided by MGI)  
Official full name: ameloblastin (provided by MGI)  
Primary source: MGI:MGI:104655  
See related: Ensembl:ENSMUSG00000029288  
Gene type: protein coding  
RefSeq status: REVIEWED  
Organism: Mus musculus  
  
Lineage: Eukaryota; Metazoa; Chordata; Craniata; Vertebrata; Euteleostomi; Mammalia;  
Eutheria; Euarchontoglires; Glires; Rodentia; Myomorpha; Muroidea; Muridae;  
Murinae; Mus; Mus  
  
  
Summary: This gene encodes an extracellular matrix glycoprotein that is involved in the  
formation of dental enamel. Mice lacking the encoded protein fail to undergo  
normal ameloblast differentiation and develop enamel. Mice overproducing the  
product of this gene develop thinner and more porous enamel, with disrupted rod  
patterns and abnormal crystallites. Alternate splicing of this gene results in  
multiple transcript variants. [provided by RefSeq, Dec 2014]  
  
Orthologsall (/gene/?Term=ortholog\_gene\_258[group]), human (https://www.ncbi.nlm.nih.gov/gene/258)”   
  
[1] “Official Symbol: AMBN (provided by HGNC)  
Official full name: ameloblastin (provided by HGNC)  
Primary source: HGNC:HGNC:452  
See related: Ensembl:ENSG00000178522; MIM:601259  
Gene type: protein coding  
RefSeq status: REVIEWED  
Organism: Homo sapiens  
  
Lineage: Eukaryota; Metazoa; Chordata; Craniata; Vertebrata; Euteleostomi; Mammalia;  
Eutheria; Euarchontoglires; Primates; Haplorrhini; Catarrhini; Hominidae; Homo  
  
Also known as: AI1F  
  
Summary: This gene encodes the nonamelogenin enamel matrix protein ameloblastin. The  
encoded protein may be important in enamel matrix formation and mineralization.  
This gene is located in the calcium-binding phosphoprotein gene cluster on  
chromosome 4. Mutations in this gene may be associated with dentinogenesis  
imperfect and autosomal dominant amylogenesis imperfect. [provided by RefSeq, Aug  
2011]  
  
Orthologsall (/gene/?Term=ortholog\_gene\_258[group]), mouse (https://www.ncbi.nlm.nih.gov/gene/11698)”

# 2 Primary Screening at CCP

## 2.1 Staff Members

### Biochemistry and Hematology

**Head:** Karel Chalupsky

**Technician(s):** Krystof Klima, Michaela Ptackova and Pavla Rachacova

### Bioimaging and Embryology

**Head:** Jan Prochazka

**Specialist:** Frantisek Spoutil

**Technician(s):** Michaela Prochazkova, Sarah Clewel, Ivana Uramova, Tereza Michalcikova and Marie Kleisnerova

### Cardiovascular

**Head:** Silvia Petrezselyova

**Technician(s):** Petr Macek

### Hearing

**Head:** Jiri Lindovsky

**Technician(s):** Miles J. Raishbrook

### Histopathology

**Head:** Sarka Suchanova

**Specialist(s):** Jan Kucera, Dagmar Zudova and Peter Makovicky

**Technician(s):** Attila Juhasz, Jana Zima, Linda Kutlíková, Vanessa Pianta and Gaia Streparola

### Immunology

**Head:** Jana Balounova

**Technician(s):** Maria Kuzmina

### Lung function

**Head:** Benoit Piavaux

### Metabolism

**Head:** J Nicole Chambers

**Specialist(s):** Jiri Lindovsky

**Technician(s):** Rajasree Sain

### Neurobiology and Behaviour

**Head:** Agnieszka Kubik-Zahorodna

**Technician(s):** Jan Majernik, Rozalie Novakova, Anastasia Smart and Vaclav Zatecka

### Vision

**Head:** Marcela Palkova

**Specialist:** Jiri Lindovsky

**Technician(s):** Miles J. Raishbrook

### Bioinformatics

**Head:** Vendula Novosadova

**Specialist:** Karla Fejfarova, Frantisek Malinka and Ashkan Zareie

## 2.2 Animal Care

At CCP, mice are housed in GM500 cages in individually and digital ventilated caging (IVC and DVC) systems (IVC and DVC System Green Line, Tecniplast, Italy) on aspen bedding (Tapvei, Estonia). The IVCs operate with positive pressure. Mice are transferred to new cages at 2 week intervals in Laminar Flow Class II changing stations (Tecniplast, Italy). Mice are fed with autoclaved standard rodent high energy breeding diet (Altromin 1314 Forti) and given reverse osmosis filtered and chlorinated water ad libitum. Light is adjusted to a 12h/12h light/dark cycle; temperature and relative humidity are maintained at 22±1 °C and 55±5 %, respectively. In specified modules husbandry conditions are adjusted according to the experiment requirements. All materials entering the breeding and experimental area are decontaminated by autoclaves or by Vaporized hydrogen peroxide (VHP). All persons entering the facility change their clothes (autoclaved jackets, trousers and socks) and shoes, wear caps, masks and gloves before entering the CCP facility. Entry into the facility is by wet or air shower depending on the barrier status of the designated room.

Outbred 6-week-old SPF CD 1 mice are used as sentinels and kept on a mixture of soiled bedding from all cages of the IVC racks connected to one IVC blower. Health monitoring is carried out by outsourced service of the sentinel mice by certified laboratories according to FELASA recommendations http://www.felasa.org.

All animals are kept according to Directive 2010/63/EU and Czech legislation.

## 2.3 Quality Assurance

All experiments and investigations carried out at the Czech Centre for Phenogenomics (CCP) are conducted according to standard operating procedures (SOPs) and with approval of the IMG ethics committee.

All data generated is validated by CCP’s expert scientists who check the biological and mathematical relevance of the data.

The constant development of staff is a vital part of CCP’s quality assurance. To this end, CCP staff members are cross trained in multiple techniques and regularly attend workshops, seminars and conferences to further develop their skills and knowledge.

## 2.4 Workflow

The Czech Centre for Phenogenomics standard primary workflow follows the international mouse phenotyping consortium (IMPC) pipeline.

## 2.5 Statistical Analysis

All numerical univariate data are analyzed using generalised mixed model. The fixed variables are always Genotype, Gender, in the case that data are dependent on weight the other fixed variable is weight, for time series data the last fixed variable is time. The random variables are Date and Experimenter. Categorical data are analyzed by Fisher test with grouping WildType Female, WildType Male, Knockout Female, Knockout Male.

# 3 Standard Screening Procedures

## 3.1 Viability and Fertility

Purpose of these tests is to assess the fertility of homozygous knockout mice and the postnatal viability, sub-viability, and lethality of homozygous mice during cohort production.

For fertility screen homozygous mice (minimum age of 8 weeks, maximum age of 14 weeks) are mated for 4-6 weeks. Strains that produce no progeny or pregnant dams after 4 to 6 weeks progress to secondary screening.

For viability screen mice are monitored for their genotypes after Het X Het breeding. At least 28 pups are genotyped. Then strains which produce no homo/hemizygotes are considered as **lethal**. Strains that produce less than normal numbers of homozygous/hemizygous male or female pups are considered as **subviable**.

## 3.2 Body Weight

The body weight of each mouse is to be measured weekly, from the 4th until the 16th week. Optional additional weighs may also be recorded.

## 3.3 Neurobiology and Behaviour

Neurobehavioral tests using transgenic animal models make it possible to understand genetic mechanisms underlying neurological and psychiatric disorders including, but not limited to, anxiety, schizophrenia, mood disorders, and Parkinson’s disease. To understand the mechanisms underlying these disorders we employ a number of tests which analyse motor abilities, cognitive functions, emotion, sensory processing as well as neurological, gait, auditory, and vision screens of rodents.

All animals are adapted to the testing room for 1hr prior to testing. Before placement of an animal in any test, the equipment is cleaned with alcohol to remove olfactory traces.

### 3.3.1 Animal emotionality and affect

To study animal emotionality and affect we put into service tests like Open Field, Elevated Plus Maze, Light/Dark box, Tail Suspension Test, and Forced Swim Test.

**Open Field**

The open Field test is conducted according to IMPC protocol and evaluates animal motility triggered by exploratory drive in a new environment (Welker 1959). It is also used as an initial screen for general anxiety elicited in a well-lit, open unprotected space (Whimbey & Denenberg 1967). Detailed and fully automated analysis of animal behaviour in open space is based on video tracking system (Viewer, Biobserve GmbH). The software distinguishes an animal as an object contrasting with the background (Fig.1). To obtain the sufficient contrast of diversely pigmented animals with simultaneous preservation of experimental conditions we are using infrared light built into the base, on which all IR translucent mazes are placed. Testing apparatus is uniformly illuminated with the light intensity of 200 lux in the centre of the field. Simultaneously four animals are tested in four separated open fields for 20 min. Testing arena is virtually divided to periphery and centre zones, where centre zone constitutes 38% of whole arena. Presence time, distance travelled, no. of visits, latency to enter centre zone, average speed, resting and activity time, velocity classes, and many others indices are automatically computed for periphery and centre zone as well as for each 5 min. interval.

**Fig. 1:** Tracking the animals in open fields

**Light-Dark Box**

LDB is divided into two compartments; the light part and enclosed dark one. The dark part constitute of one third of total apparatus area. The light part is uniformly illuminated with the light intensity of 430 lux. Each animal is allowed to freely explore the EPM for 10 minutes. Presence time, distance travelled, no. of visits, latency to enter, average speed, resting and activity time, velocity classes, and many others indices are automatically computed for both box’s compartments.

### 3.3.2 Neuromotor Abilities

Animal sense of balance and motor coordination can be evaluated in Beam Walk test and Rotarod, which also allows assessing motor learning abilities. Other neuromotor functions are assessed by grip strength measurements and detailed automated gait analysis. Grip Strength measures maximal muscle strength of forelimbs and hind limbs on an automated grip strength meter. The test can indicate neuromuscular abnormalities. Gait Analysis is based on a fully automated analysis of video records of animal foot prints. Gait analysis provides not only information about motor coordination but also detailed kinematic description of animal gait. The test can be used to studding models of Amyotrophic Lateral Sclerosis (ALS), pain/arthritis, Parkinson’s disease, muscle injury model or spinal cord injury.

**SHIRPA**

The purpose of SHIRPA assessments is to examine mice for obvious physical characteristics, behaviours, and morphological abnormalities.

After one hour of acclimatisation to the experimental room conditions the mouse is placed into the transparent jar and basic observations of animal activity and presence are recorded. Subsequently transfer arousal, animal appearance, basic reflexes, and gait in arena are estimated. Above the arena the mouse is examined for trunk curl, limb grasping, and positional passivity. At the end of the evaluation the animal is placed into the transparent tube. The tube is turned to position mouse upside down for observing its ability of righting.

**Grip Strength**

Testing is performed in the room with light intensity of 110 lux. Forelimbs and combined forelimbs and hindlimbs grip-strengths are determined using an automated meter (Bioseb, Vitrolles, France). To assess neuromuscular function, the mouse is pulled over a grid; test is recorded in three trials. To account for potential effects of body mass all grip strength measurements are normalized to body mass.

### 3.3.3 Sensorimotor Gating

**Acoustic Startle**

Animal sensorimotor gating is tested in Startle Reflex system, which allows to determine also Prepulse Inhibition (PPI). The acoustic Startle Reflex test (SR) is an automated analysis of startle reflex in response to acoustic stimuli. The test assesses sensorimotor processing by measuring both afferent sensory information transmission and efferent motor response (Yeomans and Frankland, 1995). The test can also serve as a primary screen for hearing impairment. The lack of sufficient sensory gating mechanism is thought to lead to an overflow of the sensory stimulation and disintegration of the cognitive functions (Grillon et al., 1994). SR paradigm is therefore largely used to assess the effects of putative anti-psychotics and to explore possible genetic and neurobiological mechanisms of psychosis-related behaviour. PPI is an attenuation of startle response magnitude by pre-exposure to non-startling stimulus. PPI provides operational measurement of sensorimotor gating reflecting the ability of an animal to successfully integrate relevant and inhibit irrelevant sensory information (Norris and Blumenthal, 1996). Impaired PPI is observed in schizophrenia as well as in other neuropsychiatric disorders (Swerdlow and Geyer, 1998). Testing is automated and performed in three sound proofed cabinets; protocol execution is controlled by software (Med Associates Inc., USA). Animals are habituated to the holder for 10 minutes, and then test is initiated. Four different pre-pulse magnitudes are used for pre-pulse alone and with startle stimulus. Ten repetitions of startle, pre-pulse and combination of pre-pulse with startle stimuli are conducted in pseudorandom order. The amplitude of animal response to all stimuli is automatically recorded and % of startle response decrease in PPI is computed manually.

### 3.3.4 Cognitive functions

Cued and Contextual Conditioning, Context Discrimination, Barnes Maze, Novel Object Recognition (NOR), and IntelliCage are tests used in our facility to evaluate animal learning and memory

**Cued and Contextual Conditioning**

Cued and Contextual Conditioning are based on classical Pavlovian fear conditioning, an associative learning test routinely used to study biological basis of fear, learning, and memory. Contextual, but not cued, fear conditioning is regarded as hippocampus-dependent. Although this statement is true in most of experimental designs, animals can compensate for hippocampal damage (Frankland et al, 1998). To overcome this limitation, we also employed Context Discrimination test where intact hippocampus is critical (Bidenkapp and Rudy, 2007).

Fear Conditioning (FC) is one of the forms of passive learning that can be used in many strains of mice and rats, even when more pronounced motor deficits are problematic in other learning assays. The dependent measure used in contextual and cued FC is a freezing, defined as “absence of movement except for respiration”, which takes place following pairing of an unconditioned stimulus (US) with a conditioned stimulus (CS). Aversive stimulus, foot shock, is used for US, and neutral cue, tone and a particular context, are used for CS. The associative learning take place in a cage enclosed within a soundproof cabinet (Ugo Basile, Gemonio, Italy). The cage is equipped with a stainless steel rod floor for shock delivery (Fig.2). Testing is performed in the room with light intensity of 110 lux. The acquisition trial starts with a 3 min of adaptation period, after which mice are presented with two pairings of the CS (20 sec of 4 kHz pure tone at 77 dB) and the US (a 1 sec, 0.3 mA constant current to the cage floor). The US is presented with the termination of the CS. The intra-trial interval (ITI) is 2 min. Mice remains in the chamber for 1 min after the last shock delivery. Animals are tested for contextual memory 24 hours after training. They are reintroduced to the training context and behaviour is recorded for 3 min with no CS or US presentation. Delayed cue memory is tested three hours later. Animals are placed in a novel context, with different cage wall pattern and a smooth floor texture. The freezing response to the CS is monitored for 2 min. for 2 min.

**Fig. 2:** Freezing detection of the animal in fear conditioning box

## 3.4 Metabolism

The metabolism unit uses a multi-technique approach to build a complex picture of the overall energy storage and expenditure in the whole animal model. In humans, disturbances in energy balance result in the development of obesity or anorexia nervosa which presents as weight gain or weight loss respectively. Cancer cachexia describes a syndrome of progressive weight loss, anorexia, and persistent erosion of host body cell mass in response to a malignant growth. A decline in food intake relative to energy expenditure (which may be increased, normal, or decreased) is the fundamental physiologic derangement leading to cancer-associated weight loss. The primary metabolic screen uses indirect calorimetry, intraperitoneal glucose tolerance test (IPGTT) and body composition analysis to analyse the overall energy expenditure, glucose metabolism and energy storage of the model. This data can be used to guide more in depth analysis at the molecular level to further understand the complex mechanism pathways controlling and affecting energy homeostasis.

### 3.4.1 Indirect Calorimetry

The oxygen consumption, carbon dioxide production and heat production are measured using the PhenoMaster system from TSE systems. Animals are individually housed in their home cages (Sealsafe Plus/Green line; Techniplast) with food and water available ad libitum. Food and water intake are measured for the duration of the study as well as locomotor activity (measured by infrared beam cuts). Animals are weighed & placed in individual cages. The animals are allowed to acclimatise in the system for 24 hrs. During this time,measurements are recorded, however the data obtained during this time is not used in the final analysis (Fig. 1). At the end of the experiment, animals are weighed and returned to their original home cage.

**Fig. 1:** Calorimetry workflow

### 3.4.2 Intraperitoneal glucose tolerance test (IPGTT)

The glucose tolerance test measures the clearance of an intraperitoneally injected glucose load from the body. It is used to detect disturbances in glucose metabolism that can be linked to human conditions such as diabetes or metabolic syndrome.

Animals are transferred to clean cages and fasted overnight (16-18hrs) with water available ad libitum. Animals are then weighed and the fasted blood glucose levels is recorded (T=0) from whole blood taken from the tail vein using a standard glucometer. 20% glucose solution (2g of glucose/kg body mass) in PBS is by intraperitoneal (IP) injection. The blood glucose level is measured 15, 30, 60 and 120 mins after the administration of glucose. After the final measurement, animals are returned to the original home cage and food and water available ad libitum.

## 3.5 Cardiovascular function

Echocardiography and electrocardiography (ECG) are the most frequently used techniques for diagnosing cardiovascular diseases. These methods are non-invasive and the combination of both techniques allows a first characterization of cardiovascular physiology and function of genetically modified mice.

### 3.5.1 Echocardiography

Murine echocardiography is a “gold standard” method to assess cardiovascular structure and function (systolic and diastolic) in anaesthetized mice that has been adopted from human system (Ram et al, 2011). In order to visualize the small mouse heart and record the rapid heart rates, the cardiac ultrasound imaging is acquired using the Vevo 2100 Imaging System (VisualSonics, Inc.) with a 30 MHz transducer (MS400) operating at a frequency that provides highly reliable and reproducible image quality. Echocardiography is performed on anaesthetised (isoflurane) mice and during imaging, the concentration of anaesthesia is controlled to maintain a heart rate of 450-500 beats/min. The primary echocardiographic screen includes two-dimensional (2D) B-mode (“brightness” mode) imaging view of left ventricle (LV) along the parasternal long axis (PLAX) and M-mode (“motion” mode) image of the heart in short-axis view (SAX) for accurate linear measurements of left ventricular internal dimensions (Collins et al., 2009, Moreth et al., 2014, Stypmann et al, 2009).

**Fig. 1:** Equipment of the Cardiovascular Phenotyping Unit. Upper panel – Vevo2100 Ultrasound Imaging System (VisualSonics, Inc.); bottom panel – ECGenie system (Mouse Specifics, Inc.)

A proper cardiac B-mode view allows visualization of the left atrium and ventricle, a small portion of the right ventricular wall and the output of aorta. The apex and the beginning of ascending aorta of the heart lay on the same horizontal line. M-mode echocardiography is performed at the ventricular level at the papillary muscle level and leads to a 1D high-resolution temporal course of the diameter changes of the LV and the wall thickness of the anterior and posterior LV wall in systole as well in diastole. All M-mode echocardiography measurements follow the guidelines of the American Society of Echocardiography (Sahn et al, 1978). The SAX M-mode images are used to measure left ventricular end-diastolic internal diameter (LVEDD), left ventricular end-systolic internal diameter (LVESD), and diastolic and systolic posterior wall thickness (LVPW) in three consecutive beats. Fractional shortening (FS) is calculated as FS%=[(LVEDD-LVESD)/LVEDD]x100. Ejection fraction (EF) is calculated as EF%=100 x ((LVvolD-LVvolS)LVvolD) with LVvol=((7.0/(2.4+LVID) x LVID3). The corrected left ventricular mass (LV Mass Cor) is calculated as LV Mass Cor=0.8 (1.053 x ((LVIDD + LVPWD + IVSD)3 - LVIDD3)). The Stroke volume (SV) is the volume of blood pumped from one ventricle of the heart with each beat. The Stroke volume of the LV is obtained by subtracting end-systolic volume (ESV) from end-diastolic volume (EDV). The cardiac output (CO) is the volume of blood pumped by the heart per minute. In addition, heart rate and respiratory rate are calculated by measuring three systolic intervals, respectively three respiratory intervals.

**Fig. 2:** Representative B-mode echocardiographic image of an adult mouse heart, PLAX view (a) and SAX view (b). M-mode image of the left ventricle at level of papillary muscles (c)

### 3.5.2 Electrocardiography

Genetically modified mice with a cardiovascular phenotype can be characterized by recording changes of the rhythm of the heart, these include slowing (bradycardia) or quickening (tachycardia) of the heart rate, heart rate variability and/or alterations in the length of the interval of sinus waves that are reflected in electrocardiogram (ECG) traces. ECG reflects the electrical activity of the heart and is comprised of P, Q, R, S, and T waves. The P wave is produced by atrial depolarization, the QRS pulses represent ventricular depolarization, and the T wave represents ventricular repolarization (Goldbarg et al., 1968). Many studies have been conducted to examine the patterns of activation and repolarization in the mouse heart and their relation to the electrical cardiac activity (for a review see Boukens et al., 2014). In our facility, the murine ECG is recorded in conscious mice through the animal`s paws using the ECGenie system (Mouse Specifics, Inc.). The size and spacing of disposable footplate electrodes facilitate contact between the electrodes and the paws to provide Einthoven lead II ECG (Boukens et al., 2014). For each animal, heart depolarization and repolarization intervals and amplitudes are evaluated from continuous ECG recording after 5-min acclimation period. Only runs where at least 15 ECG beats could be included in the analysis are chosen.

Data are analysed using standard protocols for ECG signal analysis by e-MouseTM (Mouse Specifics, Inc.). The software uses a peak detection algorithm to find the peak of the R-waves and to calculate heart rate (HR). The software plots its interpretation of P, Q, R, S, and T for each beat so that unfiltered noise or motion artefacts are rejected. This is followed by calculations of the mean of the ECG time intervals for each set of waveforms. The corrected QT interval (QTc) is calculated by dividing the QT interval by the square root of the preceding RR interval. QT dispersion is measured as inter-lead variability of QT intervals. The QTc dispersion was calculated as the rate corrected QT dispersion.

**Fig. 3:** Schematic representation of the murine ECG

## 3.6 Lung function

For an optimal gas-exchange in the lungs the air should be able to easily flow in and out of the lungs. Key parameters for this are the resistance the air encounters and the elasticity of the lung tissue which allows the lungs to empty themselves efficiently during the exhalation phase.

Both these parameters can be measured by the ‘Forced Oscillation’ technique in which pressure waves, called perturbations, are applied to the lungs and the response to these pressure waves is recorded and analyzed. To perform these perturbation mice are first anaesthetized with ketamine and xylazine and intubated after which they are attached to a computer controlled piston ventilator, the Flexivent FX from Scireq. Mice are ventilated at 190 breaths per minute, which allows for an optimal oxygenation and fast recovery of the oxygen saturation after perturbations.

For the analysis of the response curves of the perturbations, a mathematical model of the lungs is used. There are mainly 2 models and also 2 types of perturbation.

In the first model, called the single compartment model, the lung is modelled as a balloon, which confers the elasticity and represents the lung tissue, attached to a tube, which confers the resistance to the airflow and represents the airways. This model is simple but very robust and the perturbation applied is relatively simple, it just consists of a sinusoidal wave at a frequency of 150 cycles per minute. This perturbation is called the SnapShot-150, which is abbreviated SN in figures. From this 2 variables are calculated, the airway resistance (R) and the elastance (E) of the lungs. Sometimes the elasticity parameter is expressed as compliance (C), which is the reciprocal of the elastance (C = 1/E).

The second model, the complex phase model, is more complex and it models the airway resistance (R) and the resistance in the lung tissue, called tissue damping (G), separately. This is possible due to a difference in frequency response of these 2 structures. For the elasticity parameters, the component of the airways is negligible and thus only 1 elasticity parameter is calculated, the tissue elastance (H). As the difference between airways and lung-tissue is the frequency response of these tissues, the perturbation is a multi-frequency perturbation. We use 2 different ones in our primary screen. The first one takes 3 seconds, which gives a less precise estimate, but which can be performed in mice with severely compromised lung-functions as the period without fresh air is only 3 seconds. This manoeuver is called Quick-prime 3, abbreviated as QP3 in figures. The second manoeuver, the Prime-8, abbreviated P8, last as its name suggests, 8 seconds. For a mouse breathing normally at about 180 to 200 breaths per minute this is relatively long and could thus lead to oxygen desaturation in animals with severely compromised lung function. In this case the animal will start spontaneous breathing movements which will compromise the measurement. This estimation of the lung function parameters is more accurate as more data points are available for model fitting.

There is an additional manoeuver which is performed which is called ‘Deep inflation’ and is a forced deep inhalation. This serves 2 purposes, firstly, to measure the lung capacity of the animal and secondly to open up the lungs completely so that all parts of the lung are recruited in the consecutive manoeuvers.

All manoeuvers are executed in an automated script 5 times. If some measurements are invalid during script execution, they are repeated until 5 valid measures are obtained, with the exception of the prime-8 measurements. These measurements are not reattempted after the script if obvious oxygen desaturation occurs, as it would be harmful for the animal and would not lead to good quality data.

## 3.7 Bioimaging

The bioimaging unit is dedicated to comprehensive morphological and functional characterization of animal models by whole-body imaging systems in vivo and ex vivo. The primary tests cover morphological analysis of the entire skeleton and quantitative analysis of mineral content. Data for 3D body composition analysis is also acquired during the same scan (Judex et al., 2010). Secondary tests and supplemental approaches are established for more detailed and comprehensive characterization of skeleton related phenotypes:

- detail ex-vivo bone structure imaging with quantification of bone porosity and mineral content (osteoporosis or arthritis models), or in-vivo of selected skeleton area (e.g. femur or autopodium)
- segmentation and volumetric analysis of individual bones – for craniofacial phenotypes)
- enamel and dentin mineralization imaging (Wald et al., 2017)
- customized optical imaging including fluorescence protein detection, fluorescence probes for inflammation, tumour development or bone remodeling
- skeletal preparation and alizarin/alcian staining for bone/cartilage visualization.

Animals are anaesthetised using 20% zoletil solution. Once anaesthetized, animals are weighed and body length is recorded with dial caliper. Animals are placed into the animal restrainer and the scan is initiated using the SkyScan software.

**microCT (Skyscan 1176, Bruker)** *in vivo* scanner provides sensitive and high resolution 3D imaging based on X-ray projections with voxel size 9 – 35μm. Our imaging set up is suitable either for *in vivo* or *ex vivo* imaging of large specimen up to size of rat. *In vivo* scans can provide comprehensive 3D visualization of bones and other mineralized tissues like teeth, but also quantification of total body fat mass and lean mass (alternative to DEXA analysis) and their changes in time.

*Ex vivo* scanning mode can be used for higher resolution imaging of large body parts or use of contrast agents for imaging soft tissues (liver, kidney, heart, neuronal tissue) or imaging fixed embryos (E9.5 – E18.5) with use of appropriate contrast agents (Iodine, PTA). For complete microCT analysis post-processing of projection data is necessary, especially 3D reconstruction and further segmentation for visualization of morphological phenotypes and quantification of selected organs.

The system calibrates automatically after turning on after 3 or more days without usage, plus flat field correction is processed whenever the scanning parameters are changed.

**Optical whole body imaging system (Xtreme, Bruker)** includes highly sensitive camera for detection of emitted photons based on enzymatic activity or fluorescence. This approach is suitable for in vivo imaging with emphasis on noninvasive imaging setup for longitudinal monitoring of studied process. This imaging modality is established for inflammation, tumour progression, metastasis or cell homing experiments. We have successfully published our imaging protocol in the study of genetic regulation of DSS colitis (Brauer et al., 2015).

**Software tool box:** NRecon, DataViewer, ITK-snap, 3D-slicer, CTvox, CTanalyser, CTvolume,Imaris, microCT (Skyscan 1176, Bruker), Optical whole body imaging system (Xtreme, Bruker)

## 3.8 Auditory Brain Stem Response (Hearing)

Auditory Brainstem Response (ABR) is an acoustically evoked electrical potential recorded by needle electrodes placed subdermally on the top of animal’s head. It represents a sum of activity of brainstem neurons responsible for sound processing. Typical ABR evoked by a short sound of supra-threshold intensity is a waveform composed of several successive peaks that appear between 2 – 6 ms after the sound onset. Their amplitudes vary in the range of hundreds of nanovolts and correlate with the stimulus intensity. The latencies of individual ABR peaks reflect successive activation of the auditory nerve, the cochlear nucleus, the superior olivary complex and the inferior colliculus, which comprise the brainstem portion of the auditory pathway (for review see Biacabe et al., 2001; Møller, 2006).

The ABR recording protocol follows guidelines as described by Ingham et al., 2011. The mouse is anaesthetized by i.p. injection of Zoletil (mixture of tiletamine and zolazepam) and placed on a heating pad inside an 6 m^3^ anechoic soundproof chamber. Three subdermal needle electrodes are positioned as follows: active electrode on the vertex, reference electrode behind the left ear and ground electrode behind the right ear, see Fig. 1. The system for sound production and biosignal recording is based on TDT workstation RZ6 (Tucker-Davies Technologies, USA). Acoustic stimuli are delivered by a high quality ribbon tweeter RAAL 140-15D (RAAL Advanced Loudspeakers, Serbia) placed approximately 40 cm in front of the mouse. Each stimulus is played repeatedly and the averaged ABR signal is recorded. Initially, the ABRs are evoked by broadband stimuli (“clicks”, i.e. 100 microseconds lasting rectangular pulses) followed by bursts of pure tones (5 ms long, 1 ms rise/fall times) of 306, 2412, 1818, 12 24 and 630 kHz frequency respectively. Stimulation always starts with the lowest sound intensity of 0 dB SPL (sound pressure level) then the intensity is increased by 5 dB steps until 85 dB SPL is reached.

**Fig. 1:** mouse under general anesthesia with subcutaneous electrodes for ABR recording. Black wire - reference electrode, behind the left ear, green wire - ground electrode behind the right ear, red wire - active electrode on the vertex.

The primary output of ABR analysis is detection of hearing thresholds for the broadband stimulus (click) and for each frequency used. The threshold is defined as the lowest intensity of sound which evoked distinguishable response related to (time-locked to) the stimulus. Statistical significance of the difference in thresholds between two groups of animals is tested by two-way ANOVA with replication combined with post-hoc tests. Other parameters such as latency, amplitude and waveform shape may be examined in addition if it is of interest.

**Fig. 2:** Auditory Brainstem Response. The animal was stimulated by a pure tone of 24 kHz frequency, 5 ms duration and intensities ranging between 0 - 85 dB SPL, the stimulus onset is marked with a vertical arrow (Stim.). Each curve represents an average of 600 repetitions. The lowest sound intensity that evoked a response is referred to as the hearing threshold for the respective frequency, marked with a horizontal arrow (Thr.). Five typical peaks of ABR signal are labelled with Roman numerals I to V. Note that with increasing intensity not only the peaks are higher but they have shorter latency, too.

## 3.9 Eye morphology

Eyes are a very important sense organ for human beings. Unfortunately, many people suffer from serious ocular diseases like cataracts, glaucoma or retinal disorders and their life quality is strongly affected. Many genes are involved in a variety of ocular disorders. As the genome of the mouse is well known, the mouse is a suitable model for understanding of the biochemical, genetic and physiological mechanisms of many hereditary degenerative ocular disorders.

The vision screen uses non-invasive imaging devices to examine the anterior segment (Pentacam) and retina of the eyes (OCT). The animals are anaesthetised using 20% Zoletil and the pupils are dilated using eye drops containing 0.5% Atropin. The mouse is placed on a platform of the Pentacam and the centre of the pupil is orientated to the light-beam slit of the Pentacam´s head. This device enables the measurement of many parameters of the cornea and the lens (e.g. surface, form, opacity, thickness and density) for each eye. All 25 scanned images from different angles of the eye are carried out by a rotating Scheimpflug Camera of the Pentacam they are analysed in order to assess any abnormalities of the eye.

The optical coherent tomograph (OCT) scans, quantifies the reflection of a light beam sending from the layers of the retina and composes cross-sectional images of the retina. Each cross-section image of the retina will be evaluated and a variety of parameters are measured e.g. the thickness and the gross morphology of the retina, form and position of the optical disc, the blood vessels and their pattern.

**Fig. 1:** Representative example of the retinal fundus with the optic disc and blood vessel system (left) and a cross-sectional image of retina (right) in the wild type mouse C57Bl/6NCrl.

## 3.10 Biochemistry and Hematology

Biochemical and haematology phenotyping is based on robust primary screening developed under Eumorphia and EMPReSS, a European Mouse Phenotyping Resource for Standardised Screens. Phenotyping investigation includes also newest development in INFRAFRONTIER and IMPC consortia. These tests comprise primary phenotyping pipeline and are carried out according the standardised protocols as detailed in (IMPReSS). The Biochemical and haematology unit currently analyses various blood metabolites, ions, hormones, and enzymes of genetically modified mice compared with age matched controls. Changes in these data can be linked to metabolic and functional abnormalities of specific organs such as liver, kidney, and gastrointestinal tract.

**Blood Withdrawal and Storage**

Blood samples are taken from isoflurane-anesthetized mice by retro-bulbar sinus puncture with non-heparinized glass capillaries. Samples are collected in two different types of coated tubes (Lithium/Heparin (KABE cat # 078028) and EDTA (KABE cat # 078035). After collection each sample is mixed by gentle inversion and then kept on RT until centrifugation. Samples are centrifuged within 1 hour of collection at 5000 X g, for 10 minutes at 8°C. Once separated from the cells, plasma samples are refrigerated if analysis is delayed. If plasma cannot be analyzed fresh, samples are stored at -20oC (short-term) or -70oC (long-term) storage. Freeze/thaw cycles are avoided.

Plasma is analyzed on Beckman AU480 biochemical analyzer. All calibration and quality control samples are within expiration period and measured values fulfilled required parameters for each method used in this report.

**Hematology**

Our hematology test requires 20 μl of whole blood in EDTA coated tubes per sample. 20 μl of whole blood is diluted to final volume is 200 μl. Complete and differential blood cell counts were measured on Mindray-BC-5000Vet

## 3.11 Immunology

As a integral part of the terminal screen, immunophenotyping involves characterisation of particular immune cell populations in terms of their cellularity and phenotype using multicolor flow cytometry.

The procedures are based on standard immunophenotyping protocols of the Adult and Embryonic Phenotype Pipeline that has been agreed by the research institutions involved MPReSS -International Mouse Phenotyping Resource of Standardised Screens.

According to these guidelines, we use mouse spleenocytes for standard immunophenotyping. However, we are able to analyze peripheral blood, lymph nodes, thymi, bone marrow or peritoneal lavage samples as well.

**Fig. 1:** Gating strategy to discriminate T cell populations using Panel A

Routinelly, we utilize two panels to detect lymphoid and myeloid cells. In addition, the spleen weight is an important indicator which is also recorded. The procedure includes several quality control steps (cell viability upon digestion, number of cells/sample after acquisition).

For primary tests, we typically use three males and three females knock out (KO) animals plus three male and three female wild type controls (WT), plus optionally one or two control mice as internal controls. Flow cytometry data are acquired using BD LSRFortessaTM SORP, manualy analyzed in FlowJo software and statistically evaluated.

**Fig. 2:** Gating strategy to discriminate B cell and myeloid populations using Panel B

## 3.12 Histopathology

The histopathology screen is dedicated for macroscopic and microscopic analysis of pathological alterations occurring in mutant mouse models. The unit is comparing gross morphology and microscopic differences between wild-type and gene-engineered mouse models. A major task is to screen for set of pathologies connected to specific genetic status using the tools and techniques of the classical and molecular pathology.

### 3.12.1 Gross Morphology and Tissue Processing

**Full mouse necropsy with organ isolation**

A complete necropsy is performed to detect and record abnormal macroscopic alterations in internal and external organs, record body, spleen, liver, kidney (dx., sin.) and heart weights (according to IMPC Heart Weight SOP) and tibia length.

Samples from IMPC ‘Tissue Collection List’ (minimal number is 2 mutant males and 2 mutant females) are fixed, trimmed, processed and embedded in paraffin blocks. The femur is also prepared and blood smears and bone marrow smears are also prepared.

Images of all abnormal findings are documented and saved in database. All collected tissues are placed in a labelled jar containing a sufficient volume of fixative (minimum of 10:1 fixative to tissue). All abnormal findings are recorded according to our Centre-specific database using the standardized IMPC Gross Pathology ontology. For long-term storage, the fixed samples are stored in paraffin blocks.

**Organ sampling and trimming**

Individual organs are processed. Unless otherwise specified, organs are processed according to the *Revised guides for organ sampling and trimming in rats and mice*, published in 2003 and 2004 by the Registry of Industrial Toxicology Animal-data (RITA) and the North American Control Animal Database (NACAD) groups (Exp Toxic Pathol 55: 91-106, Exp Tox Pathol 55: 413-431, and Exp Tox Pathol 55: 433-449).

**Tissue processing and embedding**

By using a state-of-the-art automated vacuum tissue processor (Leica ASP6025), we process and paraffin-embed specimens with the highest levels of reproducibility and quality. Where applicable, decalcification to remove mineral from bone or other calcified tissues is performed prior to processing to paraffin. Tissues are embedded in paraffin using standard orientation according to IMPC Tissue Embedding and Block Banking IMPC\_BLK\_001 SOP.

### 3.12.2 Sectioning and Staining

**Sectioning**

Standard paraffin sections are cut using a Leica RM2255-FU microtome. The standard thickness of sections is 2µm.

**H&E staining**

The standard primary staining procedure is the hematoxylin and eosin (H&E) stain. The use of the Ventana Symphony automated staining system ensures high throughput processing with consistent and reproducible results.

### 3.12.3 Histopathological examination

Histopathological examination is performed using the Carl Zeiss Axio Imager.Z2 or Leica DM3000 microscope. All abnormal findings are recorded in our Centre-specific database using the standardized IMPC Histopatology ontology.

# 4 Results

## 4.1 Weight Curve - 4 week to 16 week

| STRAIN | GENDER | count |
| --- | --- | --- |
| AMB-YLF-GGG | female | 8 |
| AMB-YLF-GGG | male | 8 |
| C57Bl/6NCrl | female | 1032 |
| C57Bl/6NCrl | male | 1088 |

|  | test | Difference | p\_value | Significance |
| --- | --- | --- | --- | --- |
| c2 | CTRl vs KO | -3.0716 | 0 | TRUE |
| c3 | Female vs Male | -3.6106 | 0 | TRUE |
| c4 | Female CTRL vs Female KO | -2.6764 | 0 | TRUE |
| c5 | Male CTRL vs Male KO | -3.4667 | 0 | TRUE |
| c6 | Female KO vs Male KO | -4.0058 | 0 | TRUE |

## 4.2 Open field

**Number of animals**

| STRAIN | GENDER | count |
| --- | --- | --- |
| AMB-YLF-GGG | female | 8 |
| AMB-YLF-GGG | male | 8 |
| C57Bl/6NCrl | female | 971 |
| C57Bl/6NCrl | male | 1000 |

### 4.2.1 Center average speed - total (cm/s)

|  | test | Difference | p\_value | Significance |
| --- | --- | --- | --- | --- |
| c2 | CTRl vs KO | -0.2081 | 0.9910 | FALSE |
| c3 | Female vs Male | 0.6649 | 0.7786 | FALSE |
| c4 | Female CTRL vs Female KO | -0.7972 | 0.8651 | FALSE |
| c5 | Male CTRL vs Male KO | 0.3811 | 0.9786 | FALSE |
| c6 | Female KO vs Male KO | 1.2541 | 0.0000 | TRUE |

### 4.2.2 Center distance travelled - total (cm)

|  | test | Difference | p\_value | Significance |
| --- | --- | --- | --- | --- |
| c2 | CTRl vs KO | 5.2402 | 1.0000 | FALSE |
| c3 | Female vs Male | 112.7510 | 0.8879 | FALSE |
| c4 | Female CTRL vs Female KO | 11.2653 | 1.0000 | FALSE |
| c5 | Male CTRL vs Male KO | -0.7850 | 1.0000 | FALSE |
| c6 | Female KO vs Male KO | 106.7259 | 0.0005 | TRUE |

### 4.2.3 Center permanence time - total (s)

|  | test | Difference | p\_value | Significance |
| --- | --- | --- | --- | --- |
| c2 | CTRl vs KO | -47.2148 | 0.7200 | FALSE |
| c3 | Female vs Male | 19.1202 | 0.9731 | FALSE |
| c4 | Female CTRL vs Female KO | 14.5965 | 0.9959 | FALSE |
| c5 | Male CTRL vs Male KO | -109.0260 | 0.2988 | FALSE |
| c6 | Female KO vs Male KO | -42.6911 | 0.0000 | TRUE |

### 4.2.4 Center resting time - total (s)

|  | test | Difference | p\_value | Significance |
| --- | --- | --- | --- | --- |
| c2 | CTRl vs KO | -49.8949 | 0.6140 | FALSE |
| c3 | Female vs Male | 13.3846 | 0.9873 | FALSE |
| c4 | Female CTRL vs Female KO | 8.0997 | 0.9990 | FALSE |
| c5 | Male CTRL vs Male KO | -107.8895 | 0.2276 | FALSE |
| c6 | Female KO vs Male KO | -44.6100 | 0.0000 | TRUE |

### 4.2.5 Distance travelled - total (cm)

|  | test | Difference | p\_value | Significance |
| --- | --- | --- | --- | --- |
| c2 | CTRl vs KO | 235.8816 | 0.9300 | FALSE |
| c3 | Female vs Male | 265.3277 | 0.9045 | FALSE |
| c4 | Female CTRL vs Female KO | -109.2800 | 0.9977 | FALSE |
| c5 | Male CTRL vs Male KO | 581.0433 | 0.6695 | FALSE |
| c6 | Female KO vs Male KO | 610.4893 | 0.0000 | TRUE |

### 4.2.6 Number of center entries - total

|  | test | Difference | p\_value | Significance |
| --- | --- | --- | --- | --- |
| c2 | CTRl vs KO | 6.1985 | 0.9155 | FALSE |
| c3 | Female vs Male | 4.5527 | 0.9635 | FALSE |
| c4 | Female CTRL vs Female KO | 8.4968 | 0.9442 | FALSE |
| c5 | Male CTRL vs Male KO | 3.9002 | 0.9861 | FALSE |
| c6 | Female KO vs Male KO | 2.2544 | 0.4721 | FALSE |

### 4.2.7 Periphery average speed - total (cm/s)

|  | test | Difference | p\_value | Significance |
| --- | --- | --- | --- | --- |
| c2 | CTRl vs KO | -0.0346 | 0.9994 | FALSE |
| c3 | Female vs Male | 0.3263 | 0.6694 | FALSE |
| c4 | Female CTRL vs Female KO | -0.1263 | 0.9913 | FALSE |
| c5 | Male CTRL vs Male KO | 0.0571 | 0.9987 | FALSE |
| c6 | Female KO vs Male KO | 0.4180 | 0.0000 | TRUE |

### 4.2.8 Periphery distance travelled - total (cm)

|  | test | Difference | p\_value | Significance |
| --- | --- | --- | --- | --- |
| c2 | CTRl vs KO | 260.5428 | 0.8105 | FALSE |
| c3 | Female vs Male | 140.4710 | 0.9636 | FALSE |
| c4 | Female CTRL vs Female KO | -100.7366 | 0.9958 | FALSE |
| c5 | Male CTRL vs Male KO | 621.8222 | 0.3726 | FALSE |
| c6 | Female KO vs Male KO | 501.7504 | 0.0000 | TRUE |

### 4.2.9 Periphery permanence time - total (s)

|  | test | Difference | p\_value | Significance |
| --- | --- | --- | --- | --- |
| c2 | CTRl vs KO | 47.2101 | 0.7201 | FALSE |
| c3 | Female vs Male | -19.1043 | 0.9732 | FALSE |
| c4 | Female CTRL vs Female KO | -14.5915 | 0.9959 | FALSE |
| c5 | Male CTRL vs Male KO | 109.0117 | 0.2989 | FALSE |
| c6 | Female KO vs Male KO | 42.6973 | 0.0000 | TRUE |

### 4.2.10 Periphery resting time - total (s)

|  | test | Difference | p\_value | Significance |
| --- | --- | --- | --- | --- |
| c2 | CTRl vs KO | 38.4430 | 0.8005 | FALSE |
| c3 | Female vs Male | -30.0344 | 0.8900 | FALSE |
| c4 | Female CTRL vs Female KO | -12.1980 | 0.9971 | FALSE |
| c5 | Male CTRL vs Male KO | 89.0840 | 0.4246 | FALSE |
| c6 | Female KO vs Male KO | 20.6067 | 0.0255 | TRUE |

### 4.2.11 Whole arena average speed - total (cm/s)

|  | test | Difference | p\_value | Significance |
| --- | --- | --- | --- | --- |
| c2 | CTRl vs KO | 0.2103 | 0.9164 | FALSE |
| c3 | Female vs Male | 0.2229 | 0.9029 | FALSE |
| c4 | Female CTRL vs Female KO | -0.0779 | 0.9986 | FALSE |
| c5 | Male CTRL vs Male KO | 0.4984 | 0.6501 | FALSE |
| c6 | Female KO vs Male KO | 0.5110 | 0.0000 | TRUE |

### 4.2.12 Whole arena resting time - total (s)

|  | test | Difference | p\_value | Significance |
| --- | --- | --- | --- | --- |
| c2 | CTRl vs KO | -18.3547 | 0.7672 | FALSE |
| c3 | Female vs Male | -10.3209 | 0.9479 | FALSE |
| c4 | Female CTRL vs Female KO | -3.7404 | 0.9992 | FALSE |
| c5 | Male CTRL vs Male KO | -32.9691 | 0.5464 | FALSE |
| c6 | Female KO vs Male KO | -24.9353 | 0.0000 | TRUE |

### 4.2.13 Center average speed

|  | test | Difference | p\_value | Significance |
| --- | --- | --- | --- | --- |
| c2 | CTRl vs KO | -0.8935 | 0.6036 | FALSE |
| c3 | Female vs Male | 0.7858 | 0.6900 | FALSE |
| c4 | Female CTRL vs Female KO | -1.5438 | 0.4541 | FALSE |
| c5 | Male CTRL vs Male KO | -0.2432 | 0.9946 | FALSE |
| c6 | Female KO vs Male KO | 1.4361 | 0.0000 | TRUE |

### 4.2.14 Center distance travelled

|  | test | Difference | p\_value | Significance |
| --- | --- | --- | --- | --- |
| c2 | CTRl vs KO | -9.1066 | 0.9953 | FALSE |
| c3 | Female vs Male | 26.2594 | 0.9019 | FALSE |
| c4 | Female CTRL vs Female KO | -8.8808 | 0.9986 | FALSE |
| c5 | Male CTRL vs Male KO | -9.3323 | 0.9980 | FALSE |
| c6 | Female KO vs Male KO | 26.0337 | 0.0006 | TRUE |

### 4.2.15 Center permanence time

|  | test | Difference | p\_value | Significance |
| --- | --- | --- | --- | --- |
| c2 | CTRl vs KO | -13.2756 | 0.6047 | FALSE |
| c3 | Female vs Male | 4.6351 | 0.9719 | FALSE |
| c4 | Female CTRL vs Female KO | 1.0256 | 0.9999 | FALSE |
| c5 | Male CTRL vs Male KO | -27.5767 | 0.2518 | FALSE |
| c6 | Female KO vs Male KO | -9.6661 | 0.0000 | TRUE |

### 4.2.16 Center resting time

|  | test | Difference | p\_value | Significance |
| --- | --- | --- | --- | --- |
| c2 | CTRl vs KO | -12.2811 | 0.5737 | FALSE |
| c3 | Female vs Male | 3.2465 | 0.9859 | FALSE |
| c4 | Female CTRL vs Female KO | 0.8258 | 0.9999 | FALSE |
| c5 | Male CTRL vs Male KO | -25.3879 | 0.2254 | FALSE |
| c6 | Female KO vs Male KO | -9.8603 | 0.0000 | TRUE |

### 4.2.17 Distance travelled

|  | test | Difference | p\_value | Significance |
| --- | --- | --- | --- | --- |
| c2 | CTRl vs KO | 31.8545 | 0.9869 | FALSE |
| c3 | Female vs Male | 65.7660 | 0.9001 | FALSE |
| c4 | Female CTRL vs Female KO | -51.7922 | 0.9834 | FALSE |
| c5 | Male CTRL vs Male KO | 115.5012 | 0.7949 | FALSE |
| c6 | Female KO vs Male KO | 149.4126 | 0.0000 | TRUE |

### 4.2.18 Number of center entries

|  | test | Difference | p\_value | Significance |
| --- | --- | --- | --- | --- |
| c2 | CTRl vs KO | 1.2199 | 0.9553 | FALSE |
| c3 | Female vs Male | 1.0650 | 0.9691 | FALSE |
| c4 | Female CTRL vs Female KO | 1.8064 | 0.9636 | FALSE |
| c5 | Male CTRL vs Male KO | 0.6334 | 0.9960 | FALSE |
| c6 | Female KO vs Male KO | 0.4785 | 0.6037 | FALSE |

### 4.2.19 Periphery average speed

|  | test | Difference | p\_value | Significance |
| --- | --- | --- | --- | --- |
| c2 | CTRl vs KO | -0.0369 | 0.9992 | FALSE |
| c3 | Female vs Male | 0.3283 | 0.6622 | FALSE |
| c4 | Female CTRL vs Female KO | -0.1154 | 0.9932 | FALSE |
| c5 | Male CTRL vs Male KO | 0.0417 | 0.9995 | FALSE |
| c6 | Female KO vs Male KO | 0.4068 | 0.0000 | TRUE |

### 4.2.20 Periphery distance travelled

|  | test | Difference | p\_value | Significance |
| --- | --- | --- | --- | --- |
| c2 | CTRl vs KO | 47.0793 | 0.9112 | FALSE |
| c3 | Female vs Male | 36.5611 | 0.9558 | FALSE |
| c4 | Female CTRL vs Female KO | -38.8082 | 0.9836 | FALSE |
| c5 | Male CTRL vs Male KO | 132.9669 | 0.4927 | FALSE |
| c6 | Female KO vs Male KO | 122.4486 | 0.0000 | TRUE |

### 4.2.21 Periphery permanence time

|  | test | Difference | p\_value | Significance |
| --- | --- | --- | --- | --- |
| c2 | CTRl vs KO | 13.2947 | 0.6039 | FALSE |
| c3 | Female vs Male | -4.6632 | 0.9714 | FALSE |
| c4 | Female CTRL vs Female KO | -1.0149 | 0.9999 | FALSE |
| c5 | Male CTRL vs Male KO | 27.6043 | 0.2513 | FALSE |
| c6 | Female KO vs Male KO | 9.6465 | 0.0000 | TRUE |

### 4.2.22 Periphery resting time

|  | test | Difference | p\_value | Significance |
| --- | --- | --- | --- | --- |
| c2 | CTRl vs KO | 11.8222 | 0.6683 | FALSE |
| c3 | Female vs Male | -7.6012 | 0.8813 | FALSE |
| c4 | Female CTRL vs Female KO | -0.5557 | 1.0000 | FALSE |
| c5 | Male CTRL vs Male KO | 24.2000 | 0.3358 | FALSE |
| c6 | Female KO vs Male KO | 4.7767 | 0.0398 | TRUE |

### 4.2.23 Whole arena average speed

|  | test | Difference | p\_value | Significance |
| --- | --- | --- | --- | --- |
| c2 | CTRl vs KO | 0.1059 | 0.9869 | FALSE |
| c3 | Female vs Male | 0.2184 | 0.9009 | FALSE |
| c4 | Female CTRL vs Female KO | -0.1734 | 0.9831 | FALSE |
| c5 | Male CTRL vs Male KO | 0.3853 | 0.7945 | FALSE |
| c6 | Female KO vs Male KO | 0.4977 | 0.0000 | TRUE |

### 4.2.24 Whole arena resting time

|  | test | Difference | p\_value | Significance |
| --- | --- | --- | --- | --- |
| c2 | CTRl vs KO | -2.9444 | 0.9192 | FALSE |
| c3 | Female vs Male | -2.5276 | 0.9469 | FALSE |
| c4 | Female CTRL vs Female KO | 0.5210 | 0.9998 | FALSE |
| c5 | Male CTRL vs Male KO | -6.4098 | 0.7173 | FALSE |
| c6 | Female KO vs Male KO | -5.9929 | 0.0000 | TRUE |

### 4.2.25 Latency to center entry (s)

|  | test | Difference | p\_value | Significance |
| --- | --- | --- | --- | --- |
| c2 | CTRl vs KO | -21.2142 | 0.8420 | FALSE |
| c3 | Female vs Male | -13.6328 | 0.9502 | FALSE |
| c4 | Female CTRL vs Female KO | -23.1023 | 0.9249 | FALSE |
| c5 | Male CTRL vs Male KO | -19.3262 | 0.9461 | FALSE |
| c6 | Female KO vs Male KO | -11.7447 | 0.0397 | TRUE |

### 4.2.26 Percentage center time - total (%)

|  | test | Difference | p\_value | Significance |
| --- | --- | --- | --- | --- |
| c2 | CTRl vs KO | -3.9363 | 0.7198 | FALSE |
| c3 | Female vs Male | 1.5941 | 0.9731 | FALSE |
| c4 | Female CTRL vs Female KO | 1.2153 | 0.9959 | FALSE |
| c5 | Male CTRL vs Male KO | -9.0880 | 0.2986 | FALSE |
| c6 | Female KO vs Male KO | -3.5575 | 0.0000 | TRUE |

## 4.3 Shirpa and Dismorphology

**Number of animals**

| STRAIN | GENDER | count |
| --- | --- | --- |
| AMB-YLF-GGG | female | 8 |
| AMB-YLF-GGG | male | 8 |
| C57Bl/6NCrl | female | 982 |
| C57Bl/6NCrl | male | 1002 |

### 4.3.1 Activity (body position)

### 4.3.2 Aggression

|  | female AMB-YLF-GGG | female C57Bl/6NCrl | male AMB-YLF-GGG |
| --- | --- | --- | --- |
| female C57Bl/6NCrl | 1 | NA | NA |
| male AMB-YLF-GGG | 1 | 1 | NA |
| male C57Bl/6NCrl | 1 | 1 | 1 |

### 4.3.3 Coat - color - abdomen

|  | female AMB-YLF-GGG | female C57Bl/6NCrl | male AMB-YLF-GGG |
| --- | --- | --- | --- |
| female C57Bl/6NCrl | 1 | NA | NA |
| male AMB-YLF-GGG | 1 | 1 | NA |
| male C57Bl/6NCrl | 1 | 1 | 1 |

### 4.3.4 Coat - color - back

### 4.3.5 Coat - color - head

|  | female AMB-YLF-GGG | female C57Bl/6NCrl | male AMB-YLF-GGG |
| --- | --- | --- | --- |
| female C57Bl/6NCrl | 1 | NA | NA |
| male AMB-YLF-GGG | 1 | 1 | NA |
| male C57Bl/6NCrl | 1 | 1 | 1 |

### 4.3.6 Coat - color pattern - abdomen

|  | female AMB-YLF-GGG | female C57Bl/6NCrl | male AMB-YLF-GGG |
| --- | --- | --- | --- |
| female C57Bl/6NCrl | 1 | NA | NA |
| male AMB-YLF-GGG | 1 | 1 | NA |
| male C57Bl/6NCrl | 1 | 1 | 1 |

### 4.3.7 Coat - color pattern - back

### 4.3.8 Coat - color pattern - head

### 4.3.9 Coat - hair distribution - abdomen

|  | female AMB-YLF-GGG | female C57Bl/6NCrl | male AMB-YLF-GGG |
| --- | --- | --- | --- |
| female C57Bl/6NCrl | 1 | NA | NA |
| male AMB-YLF-GGG | 1 | 1 | NA |
| male C57Bl/6NCrl | 1 | 1 | 1 |

### 4.3.10 Coat - hair distribution - back

|  | female AMB-YLF-GGG | female C57Bl/6NCrl | male AMB-YLF-GGG |
| --- | --- | --- | --- |
| female C57Bl/6NCrl | 1 | NA | NA |
| male AMB-YLF-GGG | 1 | 1 | NA |
| male C57Bl/6NCrl | 1 | 1 | 1 |

### 4.3.11 Coat - hair distribution - head

|  | female AMB-YLF-GGG | female C57Bl/6NCrl | male AMB-YLF-GGG |
| --- | --- | --- | --- |
| female C57Bl/6NCrl | 1 | NA | NA |
| male AMB-YLF-GGG | 1 | 1 | NA |
| male C57Bl/6NCrl | 1 | 1 | 1 |

### 4.3.12 Coat - hair texture\_appearance - abdomen

|  | female AMB-YLF-GGG | female C57Bl/6NCrl | male AMB-YLF-GGG |
| --- | --- | --- | --- |
| female C57Bl/6NCrl | 1 | NA | NA |
| male AMB-YLF-GGG | 1 | 1 | NA |
| male C57Bl/6NCrl | 1 | 1 | 1 |

### 4.3.13 Coat - hair texture\_appearance - back

|  | female AMB-YLF-GGG | female C57Bl/6NCrl | male AMB-YLF-GGG |
| --- | --- | --- | --- |
| female C57Bl/6NCrl | 1 | NA | NA |
| male AMB-YLF-GGG | 1 | 1 | NA |
| male C57Bl/6NCrl | 1 | 1 | 1 |

### 4.3.14 Coat - hair texture\_appearance - head

|  | female AMB-YLF-GGG | female C57Bl/6NCrl | male AMB-YLF-GGG |
| --- | --- | --- | --- |
| female C57Bl/6NCrl | 1 | NA | NA |
| male AMB-YLF-GGG | 1 | 1 | NA |
| male C57Bl/6NCrl | 1 | 1 | 1 |

### 4.3.15 Contact righting

|  | female AMB-YLF-GGG | female C57Bl/6NCrl | male AMB-YLF-GGG |
| --- | --- | --- | --- |
| female C57Bl/6NCrl | 1 | NA | NA |
| male AMB-YLF-GGG | 1 | 1 | NA |
| male C57Bl/6NCrl | 1 | 1 | 1 |

### 4.3.16 Ears

### 4.3.17 Forelimb digit - number

### 4.3.18 Forelimb digit - shape

### 4.3.19 Forelimb digit - size

### 4.3.20 Forelimb nail - length

### 4.3.21 Forelimb nail - number

### 4.3.22 Forelimb nail - shape

### 4.3.23 Forelimbs - position

### 4.3.24 Forelimbs - shape

|  | female AMB-YLF-GGG | female C57Bl/6NCrl | male AMB-YLF-GGG |
| --- | --- | --- | --- |
| female C57Bl/6NCrl | 1 | NA | NA |
| male AMB-YLF-GGG | 1 | 1 | NA |
| male C57Bl/6NCrl | 1 | 1 | 1 |

### 4.3.25 Forelimbs - size

### 4.3.26 Forepaw - shape

### 4.3.27 Forepaw - size

### 4.3.28 Gait

|  | female AMB-YLF-GGG | female C57Bl/6NCrl | male AMB-YLF-GGG |
| --- | --- | --- | --- |
| female C57Bl/6NCrl | 1 | NA | NA |
| male AMB-YLF-GGG | 1 | 1 | NA |
| male C57Bl/6NCrl | 1 | 1 | 1 |

### 4.3.29 Genitalia - morphology

|  | female AMB-YLF-GGG | female C57Bl/6NCrl | male AMB-YLF-GGG |
| --- | --- | --- | --- |
| female C57Bl/6NCrl | 1 | NA | NA |
| male AMB-YLF-GGG | 1 | 1.0000 | NA |
| male C57Bl/6NCrl | 1 | 0.7482 | 1 |

### 4.3.30 Genitalia - presence

### 4.3.31 Genitalia - size

### 4.3.32 Head bobbing

### 4.3.33 Head morphology

|  | female AMB-YLF-GGG | female C57Bl/6NCrl | male AMB-YLF-GGG |
| --- | --- | --- | --- |
| female C57Bl/6NCrl | 1 | NA | NA |
| male AMB-YLF-GGG | 1 | 1 | NA |
| male C57Bl/6NCrl | 1 | 1 | 1 |

### 4.3.34 Head size

### 4.3.35 Hindlimb digit - number

|  | female AMB-YLF-GGG | female C57Bl/6NCrl | male AMB-YLF-GGG |
| --- | --- | --- | --- |
| female C57Bl/6NCrl | 1 | NA | NA |
| male AMB-YLF-GGG | 1 | 1 | NA |
| male C57Bl/6NCrl | 1 | 1 | 1 |

### 4.3.36 Hindlimb digit - shape

### 4.3.37 Hindlimb digit - size

### 4.3.38 Hindlimb nail - length

### 4.3.39 Hindlimb nail - number

### 4.3.40 Hindlimb nail - shape

### 4.3.41 Hindlimbs - position

### 4.3.42 Hindlimbs - shape

|  | female AMB-YLF-GGG | female C57Bl/6NCrl | male AMB-YLF-GGG |
| --- | --- | --- | --- |
| female C57Bl/6NCrl | 1 | NA | NA |
| male AMB-YLF-GGG | 1 | 1 | NA |
| male C57Bl/6NCrl | 1 | 1 | 1 |

### 4.3.43 Hindlimbs - size

|  | female AMB-YLF-GGG | female C57Bl/6NCrl | male AMB-YLF-GGG |
| --- | --- | --- | --- |
| female C57Bl/6NCrl | 1 | NA | NA |
| male AMB-YLF-GGG | 1 | 1 | NA |
| male C57Bl/6NCrl | 1 | 1 | 1 |

### 4.3.44 Hindpaw - shape

### 4.3.45 Hindpaw - size

### 4.3.46 Limb grasp

|  | female AMB-YLF-GGG | female C57Bl/6NCrl | male AMB-YLF-GGG |
| --- | --- | --- | --- |
| female C57Bl/6NCrl | 1 | NA | NA |
| male AMB-YLF-GGG | 1 | 1 | NA |
| male C57Bl/6NCrl | 1 | 1 | 1 |

### 4.3.47 Lower lip morphology

### 4.3.48 Lower teeth appearance

|  | female AMB-YLF-GGG | female C57Bl/6NCrl | male AMB-YLF-GGG |
| --- | --- | --- | --- |
| female C57Bl/6NCrl | 1 | NA | NA |
| male AMB-YLF-GGG | 1 | 1 | NA |
| male C57Bl/6NCrl | 1 | 1 | 1 |

### 4.3.49 Mouth morphology

|  | female AMB-YLF-GGG | female C57Bl/6NCrl | male AMB-YLF-GGG |
| --- | --- | --- | --- |
| female C57Bl/6NCrl | 1 | NA | NA |
| male AMB-YLF-GGG | 1 | 1 | NA |
| male C57Bl/6NCrl | 1 | 1 | 1 |

### 4.3.50 Skin color - back paws

### 4.3.51 Skin color - ear

### 4.3.52 Skin color - front paws

### 4.3.53 Skin color - snout

### 4.3.54 Skin color - tail

### 4.3.55 Skin color - whole body

### 4.3.56 Skin texture - back paws

### 4.3.57 Skin texture - ear

### 4.3.58 Skin texture - front paws

|  | female AMB-YLF-GGG | female C57Bl/6NCrl | male AMB-YLF-GGG |
| --- | --- | --- | --- |
| female C57Bl/6NCrl | 1 | NA | NA |
| male AMB-YLF-GGG | 1 | 1 | NA |
| male C57Bl/6NCrl | 1 | 1 | 1 |

### 4.3.59 Skin texture - snout

### 4.3.60 Skin texture - tail

### 4.3.61 Skin texture - whole body

### 4.3.62 Snout size

### 4.3.63 Startle response

|  | female AMB-YLF-GGG | female C57Bl/6NCrl | male AMB-YLF-GGG |
| --- | --- | --- | --- |
| female C57Bl/6NCrl | 1 | NA | NA |
| male AMB-YLF-GGG | 1 | 1.0000 | NA |
| male C57Bl/6NCrl | 1 | 0.7264 | 1 |

### 4.3.64 Tail - length

|  | female AMB-YLF-GGG | female C57Bl/6NCrl | male AMB-YLF-GGG |
| --- | --- | --- | --- |
| female C57Bl/6NCrl | 1 | NA | NA |
| male AMB-YLF-GGG | 1 | 1 | NA |
| male C57Bl/6NCrl | 1 | 1 | 1 |

### 4.3.65 Tail - morphology

|  | female AMB-YLF-GGG | female C57Bl/6NCrl | male AMB-YLF-GGG |
| --- | --- | --- | --- |
| female C57Bl/6NCrl | 1 | NA | NA |
| male AMB-YLF-GGG | 1 | 1.0000 | NA |
| male C57Bl/6NCrl | 1 | 0.3735 | 1 |

### 4.3.66 Tail - presence

### 4.3.67 Tail - thickness

### 4.3.68 Tail elevation

### 4.3.69 Teeth presence

### 4.3.70 Transfer arousal

### 4.3.71 Tremor

|  | female AMB-YLF-GGG | female C57Bl/6NCrl | male AMB-YLF-GGG |
| --- | --- | --- | --- |
| female C57Bl/6NCrl | 1 | NA | NA |
| male AMB-YLF-GGG | 1 | 1 | NA |
| male C57Bl/6NCrl | 1 | 1 | 1 |

### 4.3.72 Trunk curl

|  | female AMB-YLF-GGG | female C57Bl/6NCrl | male AMB-YLF-GGG |
| --- | --- | --- | --- |
| female C57Bl/6NCrl | 1 | NA | NA |
| male AMB-YLF-GGG | 1 | 1 | NA |
| male C57Bl/6NCrl | 1 | 1 | 1 |

### 4.3.73 Unexpected behaviors

|  | female AMB-YLF-GGG | female C57Bl/6NCrl | male AMB-YLF-GGG |
| --- | --- | --- | --- |
| female C57Bl/6NCrl | 1 | NA | NA |
| male AMB-YLF-GGG | 1 | 0.1401 | NA |
| male C57Bl/6NCrl | 1 | 1.0000 | 0.1401 |

### 4.3.74 Upper lip morphology

### 4.3.75 Upper teeth appearance

|  | female AMB-YLF-GGG | female C57Bl/6NCrl | male AMB-YLF-GGG |
| --- | --- | --- | --- |
| female C57Bl/6NCrl | 1 | NA | NA |
| male AMB-YLF-GGG | 1 | 1 | NA |
| male C57Bl/6NCrl | 1 | 1 | 1 |

### 4.3.76 Vibrissae - appearance

|  | female AMB-YLF-GGG | female C57Bl/6NCrl | male AMB-YLF-GGG |
| --- | --- | --- | --- |
| female C57Bl/6NCrl | 1 | NA | NA |
| male AMB-YLF-GGG | 1 | 1 | NA |
| male C57Bl/6NCrl | 1 | 1 | 1 |

### 4.3.77 Vibrissae - presence

|  | female AMB-YLF-GGG | female C57Bl/6NCrl | male AMB-YLF-GGG |
| --- | --- | --- | --- |
| female C57Bl/6NCrl | 1 | NA | NA |
| male AMB-YLF-GGG | 1 | 1 | NA |
| male C57Bl/6NCrl | 1 | 1 | 1 |

### 4.3.78 Vocalization

|  | female AMB-YLF-GGG | female C57Bl/6NCrl | male AMB-YLF-GGG |
| --- | --- | --- | --- |
| female C57Bl/6NCrl | 1 | NA | NA |
| male AMB-YLF-GGG | 1 | 1 | NA |
| male C57Bl/6NCrl | 1 | 1 | 1 |

## 4.4 Grip Strength

**Number of animals**

| STRAIN | GENDER | count |
| --- | --- | --- |
| AMB-YLF-GGG | female | 8 |
| AMB-YLF-GGG | male | 8 |
| C57Bl/6NCrl | female | 982 |
| C57Bl/6NCrl | male | 1003 |

|  | test | Difference | p\_value | Significance |
| --- | --- | --- | --- | --- |
| c2 | CTRl vs KO | -26.9982 | 0.0024 | TRUE |
| c3 | Female vs Male | -11.2344 | 0.4311 | FALSE |
| c4 | Female CTRL vs Female KO | -29.2309 | 0.0563 | FALSE |
| c5 | Male CTRL vs Male KO | -24.7656 | 0.0524 | FALSE |
| c6 | Female KO vs Male KO | -9.0017 | 0.0000 | TRUE |

|  | test | Difference | p\_value | Significance |
| --- | --- | --- | --- | --- |
| c2 | CTRl vs KO | -45.7434 | 0.0028 | TRUE |
| c3 | Female vs Male | -14.8929 | 0.6467 | FALSE |
| c4 | Female CTRL vs Female KO | -44.7537 | 0.1307 | FALSE |
| c5 | Male CTRL vs Male KO | -46.7331 | 0.0160 | TRUE |
| c6 | Female KO vs Male KO | -15.8826 | 0.0000 | TRUE |

## 4.5 Light Dark Box

**Number of animals**

| STRAIN | GENDER | count |
| --- | --- | --- |
| AMB-YLF-GGG | female | 8 |
| AMB-YLF-GGG | male | 8 |
| C57Bl/6NCrl | female | 741 |
| C57Bl/6NCrl | male | 757 |

### 4.5.1 Dark side time spent

|  | test | Difference | p\_value | Significance |
| --- | --- | --- | --- | --- |
| c2 | CTRl vs KO | -17.3365 | 0.7654 | FALSE |
| c3 | Female vs Male | -15.5162 | 0.8194 | FALSE |
| c4 | Female CTRL vs Female KO | -25.7006 | 0.7992 | FALSE |
| c5 | Male CTRL vs Male KO | -8.9724 | 0.9757 | FALSE |
| c6 | Female KO vs Male KO | -7.1521 | 0.1271 | FALSE |

### 4.5.2 Light side time spent

|  | test | Difference | p\_value | Significance |
| --- | --- | --- | --- | --- |
| c2 | CTRl vs KO | 15.7991 | 0.7891 | FALSE |
| c3 | Female vs Male | 16.8092 | 0.7548 | FALSE |
| c4 | Female CTRL vs Female KO | 25.6422 | 0.7701 | FALSE |
| c5 | Male CTRL vs Male KO | 5.9559 | 0.9920 | FALSE |
| c6 | Female KO vs Male KO | 6.9660 | 0.1181 | FALSE |

### 4.5.3 Latency to first transition

|  | test | Difference | p\_value | Significance |
| --- | --- | --- | --- | --- |
| c2 | CTRl vs KO | -5.0919 | 0.9969 | FALSE |
| c3 | Female vs Male | -21.4144 | 0.8134 | FALSE |
| c4 | Female CTRL vs Female KO | -3.1102 | 0.9998 | FALSE |
| c5 | Male CTRL vs Male KO | -7.0737 | 0.9966 | FALSE |
| c6 | Female KO vs Male KO | -23.3962 | 0.0000 | TRUE |

### 4.5.4 Side changes

|  | test | Difference | p\_value | Significance |
| --- | --- | --- | --- | --- |
| c2 | CTRl vs KO | 3.0642 | 0.0665 | FALSE |
| c3 | Female vs Male | 0.3965 | 0.9876 | FALSE |
| c4 | Female CTRL vs Female KO | 2.8382 | 0.3965 | FALSE |
| c5 | Male CTRL vs Male KO | 3.2902 | 0.1888 | FALSE |
| c6 | Female KO vs Male KO | 0.6224 | 0.0369 | TRUE |

### 4.5.5 Time mobile active dark side

|  | test | Difference | p\_value | Significance |
| --- | --- | --- | --- | --- |
| c2 | CTRl vs KO | -4.0894 | 0.9259 | FALSE |
| c3 | Female vs Male | -11.9455 | 0.2724 | FALSE |
| c4 | Female CTRL vs Female KO | -24.4211 | 0.0795 | FALSE |
| c5 | Male CTRL vs Male KO | 16.2423 | 0.2313 | FALSE |
| c6 | Female KO vs Male KO | 8.3863 | 0.0000 | TRUE |

### 4.5.6 Time mobile active light side

|  | test | Difference | p\_value | Significance |
| --- | --- | --- | --- | --- |
| c2 | CTRl vs KO | 5.9546 | 0.4246 | FALSE |
| c3 | Female vs Male | 9.3523 | 0.0808 | FALSE |
| c4 | Female CTRL vs Female KO | 11.3714 | 0.1929 | FALSE |
| c5 | Male CTRL vs Male KO | 0.5377 | 0.9996 | FALSE |
| c6 | Female KO vs Male KO | 3.9355 | 0.0000 | TRUE |

## 4.6 Acustic startle/PPI

**Number of animals**

| STRAIN | GENDER | count |
| --- | --- | --- |
| AMB-YLF-GGG | female | 8 |
| AMB-YLF-GGG | male | 8 |
| C57Bl/6NCrl | female | 970 |
| C57Bl/6NCrl | male | 992 |

### 4.6.1 % Pre-pulse inhibition - Global

|  | test | Difference | p\_value | Significance |
| --- | --- | --- | --- | --- |
| c2 | CTRl vs KO | -3.5182 | 0.9299 | FALSE |
| c3 | Female vs Male | -1.4772 | 0.9941 | FALSE |
| c4 | Female CTRL vs Female KO | -4.0995 | 0.9664 | FALSE |
| c5 | Male CTRL vs Male KO | -2.9369 | 0.9794 | FALSE |
| c6 | Female KO vs Male KO | -0.8959 | 0.7907 | FALSE |

### 4.6.2 % Pre-pulse inhibition - PPI1

|  | test | Difference | p\_value | Significance |
| --- | --- | --- | --- | --- |
| c2 | CTRl vs KO | -2.3482 | 0.9745 | FALSE |
| c3 | Female vs Male | -0.1579 | 1.0000 | FALSE |
| c4 | Female CTRL vs Female KO | -3.8315 | 0.9635 | FALSE |
| c5 | Male CTRL vs Male KO | -0.8649 | 0.9995 | FALSE |
| c6 | Female KO vs Male KO | 1.3254 | 0.5064 | FALSE |

### 4.6.3 % Pre-pulse inhibition - PPI2

|  | test | Difference | p\_value | Significance |
| --- | --- | --- | --- | --- |
| c2 | CTRl vs KO | -1.7062 | 0.9931 | FALSE |
| c3 | Female vs Male | -1.3308 | 0.9966 | FALSE |
| c4 | Female CTRL vs Female KO | -2.9223 | 0.9898 | FALSE |
| c5 | Male CTRL vs Male KO | -0.4902 | 0.9999 | FALSE |
| c6 | Female KO vs Male KO | -0.1147 | 0.9995 | FALSE |

### 4.6.4 % Pre-pulse inhibition - PPI3

|  | test | Difference | p\_value | Significance |
| --- | --- | --- | --- | --- |
| c2 | CTRl vs KO | -2.5426 | 0.9782 | FALSE |
| c3 | Female vs Male | -0.5514 | 0.9998 | FALSE |
| c4 | Female CTRL vs Female KO | -1.2904 | 0.9992 | FALSE |
| c5 | Male CTRL vs Male KO | -3.7949 | 0.9658 | FALSE |
| c6 | Female KO vs Male KO | -1.8037 | 0.3277 | FALSE |

### 4.6.5 % Pre-pulse inhibition - PPI4

|  | test | Difference | p\_value | Significance |
| --- | --- | --- | --- | --- |
| c2 | CTRl vs KO | -7.1971 | 0.6163 | FALSE |
| c3 | Female vs Male | -4.3364 | 0.8808 | FALSE |
| c4 | Female CTRL vs Female KO | -8.5174 | 0.7781 | FALSE |
| c5 | Male CTRL vs Male KO | -5.8768 | 0.8658 | FALSE |
| c6 | Female KO vs Male KO | -3.0161 | 0.0149 | TRUE |

## 4.7 Fear Conditioning

**Number of animals**

| STRAIN | GENDER | count |
| --- | --- | --- |
| AMB-YLF-GGG | female | 8 |
| AMB-YLF-GGG | male | 8 |
| C57Bl/6NCrl | female | 981 |
| C57Bl/6NCrl | male | 1001 |

|  | test | Difference | p\_value | Significance |
| --- | --- | --- | --- | --- |
| c2 | CTRl vs KO | 1.7860 | 0.9926 | FALSE |
| c3 | Female vs Male | 3.7244 | 0.2579 | FALSE |
| c4 | Female CTRL vs Female KO | 3.4233 | 0.9268 | FALSE |
| c5 | Male CTRL vs Male KO | 0.2563 | 1.0000 | FALSE |
| c6 | Female KO vs Male KO | 2.1947 | 0.0019 | TRUE |
| c7 | CTRl vs KO for baseline FT | 0.9248 | 0.9999 | FALSE |
| c8 | CTRl vs KO for Context FT | 0.7944 | 0.9999 | FALSE |
| c9 | CTRl vs KO for Cue FT | 0.9716 | 0.9973 | FALSE |

## 4.8 Echocardiography

**Number of animals**

| STRAIN | GENDER | count |
| --- | --- | --- |
| AMB-YLF-GGG | female | 8 |
| AMB-YLF-GGG | male | 8 |
| C57Bl/6NCrl | female | 934 |
| C57Bl/6NCrl | male | 938 |

### 4.8.1 Aortic diameter (Dao)

|  | test | Difference | p\_value | Significance |
| --- | --- | --- | --- | --- |
| c2 | CTRl vs KO | -0.0776 | 0.4372 | FALSE |
| c3 | Female vs Male | -0.0285 | 0.9449 | FALSE |
| c4 | Female CTRL vs Female KO | -0.0830 | 0.7151 | FALSE |
| c5 | Male CTRL vs Male KO | -0.0721 | 0.6985 | FALSE |
| c6 | Female KO vs Male KO | -0.0231 | 0.0015 | TRUE |

### 4.8.2 Cardiac Output

|  | test | Difference | p\_value | Significance |
| --- | --- | --- | --- | --- |
| c2 | CTRl vs KO | -1.1363 | 0.7481 | FALSE |
| c3 | Female vs Male | -2.2760 | 0.1929 | FALSE |
| c4 | Female CTRL vs Female KO | -1.3379 | 0.8479 | FALSE |
| c5 | Male CTRL vs Male KO | -0.9347 | 0.9314 | FALSE |
| c6 | Female KO vs Male KO | -2.0745 | 0.0000 | TRUE |

### 4.8.3 Ejection Fraction

|  | test | Difference | p\_value | Significance |
| --- | --- | --- | --- | --- |
| c2 | CTRl vs KO | 0.6482 | 0.9943 | FALSE |
| c3 | Female vs Male | 6.5114 | 0.0603 | FALSE |
| c4 | Female CTRL vs Female KO | 5.0033 | 0.5513 | FALSE |
| c5 | Male CTRL vs Male KO | -3.7068 | 0.7049 | FALSE |
| c6 | Female KO vs Male KO | 2.1564 | 0.0000 | TRUE |

### 4.8.4 End-Diastolic Diameter

|  | test | Difference | p\_value | Significance |
| --- | --- | --- | --- | --- |
| c2 | CTRl vs KO | -0.1726 | 0.2358 | FALSE |
| c3 | Female vs Male | -0.3878 | 0.0002 | TRUE |
| c4 | Female CTRL vs Female KO | -0.3019 | 0.1056 | FALSE |
| c5 | Male CTRL vs Male KO | -0.0434 | 0.9857 | FALSE |
| c6 | Female KO vs Male KO | -0.2586 | 0.0000 | TRUE |

### 4.8.5 End-Systolic Diameter

|  | test | Difference | p\_value | Significance |
| --- | --- | --- | --- | --- |
| c2 | CTRl vs KO | -0.1489 | 0.5308 | FALSE |
| c3 | Female vs Male | -0.4425 | 0.0005 | TRUE |
| c4 | Female CTRL vs Female KO | -0.3422 | 0.1484 | FALSE |
| c5 | Male CTRL vs Male KO | 0.0445 | 0.9910 | FALSE |
| c6 | Female KO vs Male KO | -0.2491 | 0.0000 | TRUE |

### 4.8.6 Fractional Shortening

|  | test | Difference | p\_value | Significance |
| --- | --- | --- | --- | --- |
| c2 | CTRl vs KO | 0.3276 | 0.9967 | FALSE |
| c3 | Female vs Male | 3.9031 | 0.0681 | FALSE |
| c4 | Female CTRL vs Female KO | 3.0372 | 0.5573 | FALSE |
| c5 | Male CTRL vs Male KO | -2.3821 | 0.6712 | FALSE |
| c6 | Female KO vs Male KO | 1.1934 | 0.0000 | TRUE |

### 4.8.7 Heart rate

|  | test | Difference | p\_value | Significance |
| --- | --- | --- | --- | --- |
| c2 | CTRl vs KO | 16.8834 | 0.7792 | FALSE |
| c3 | Female vs Male | -16.5044 | 0.7903 | FALSE |
| c4 | Female CTRL vs Female KO | 7.0872 | 0.9936 | FALSE |
| c5 | Male CTRL vs Male KO | 26.6796 | 0.6597 | FALSE |
| c6 | Female KO vs Male KO | -6.7082 | 0.0131 | TRUE |

### 4.8.8 Left ventricle anterior wall (diastole)

|  | test | Difference | p\_value | Significance |
| --- | --- | --- | --- | --- |
| c2 | CTRl vs KO | -0.0265 | 0.9069 | FALSE |
| c3 | Female vs Male | -0.0085 | 0.9964 | FALSE |
| c4 | Female CTRL vs Female KO | -0.0335 | 0.9347 | FALSE |
| c5 | Male CTRL vs Male KO | -0.0194 | 0.9842 | FALSE |
| c6 | Female KO vs Male KO | -0.0014 | 0.9947 | FALSE |

### 4.8.9 Left ventricle anterior wall (systole)

|  | test | Difference | p\_value | Significance |
| --- | --- | --- | --- | --- |
| c2 | CTRl vs KO | 0.0270 | 0.9668 | FALSE |
| c3 | Female vs Male | 0.0834 | 0.4836 | FALSE |
| c4 | Female CTRL vs Female KO | 0.1055 | 0.6317 | FALSE |
| c5 | Male CTRL vs Male KO | -0.0515 | 0.9051 | FALSE |
| c6 | Female KO vs Male KO | 0.0049 | 0.9001 | FALSE |

### 4.8.10 Left ventricle interior diameter (diastole)

|  | test | Difference | p\_value | Significance |
| --- | --- | --- | --- | --- |
| c2 | CTRl vs KO | -0.1463 | 0.3970 | FALSE |
| c3 | Female vs Male | -0.3605 | 0.0010 | TRUE |
| c4 | Female CTRL vs Female KO | -0.2514 | 0.2456 | FALSE |
| c5 | Male CTRL vs Male KO | -0.0412 | 0.9884 | FALSE |
| c6 | Female KO vs Male KO | -0.2554 | 0.0000 | TRUE |

### 4.8.11 Left ventricle interior diameter (systole)

|  | test | Difference | p\_value | Significance |
| --- | --- | --- | --- | --- |
| c2 | CTRl vs KO | -0.2236 | 0.2621 | FALSE |
| c3 | Female vs Male | -0.4512 | 0.0019 | TRUE |
| c4 | Female CTRL vs Female KO | -0.4257 | 0.0830 | FALSE |
| c5 | Male CTRL vs Male KO | -0.0215 | 0.9992 | FALSE |
| c6 | Female KO vs Male KO | -0.2491 | 0.0000 | TRUE |

### 4.8.12 Left ventricle posterior wall (diastole)

|  | test | Difference | p\_value | Significance |
| --- | --- | --- | --- | --- |
| c2 | CTRl vs KO | -0.0230 | 0.8705 | FALSE |
| c3 | Female vs Male | -0.0136 | 0.9691 | FALSE |
| c4 | Female CTRL vs Female KO | -0.0005 | 1.0000 | FALSE |
| c5 | Male CTRL vs Male KO | -0.0455 | 0.6922 | FALSE |
| c6 | Female KO vs Male KO | -0.0361 | 0.0000 | TRUE |

### 4.8.13 Left ventricle posterior wall (systole)

|  | test | Difference | p\_value | Significance |
| --- | --- | --- | --- | --- |
| c2 | CTRl vs KO | -0.0503 | 0.6415 | FALSE |
| c3 | Female vs Male | 0.0038 | 0.9997 | FALSE |
| c4 | Female CTRL vs Female KO | -0.0081 | 0.9992 | FALSE |
| c5 | Male CTRL vs Male KO | -0.0926 | 0.4079 | FALSE |
| c6 | Female KO vs Male KO | -0.0384 | 0.0000 | TRUE |

### 4.8.14 Stroke Volume

|  | test | Difference | p\_value | Significance |
| --- | --- | --- | --- | --- |
| c2 | CTRl vs KO | -3.5165 | 0.3898 | FALSE |
| c3 | Female vs Male | -3.2107 | 0.4702 | FALSE |
| c4 | Female CTRL vs Female KO | -3.0778 | 0.7781 | FALSE |
| c5 | Male CTRL vs Male KO | -3.9552 | 0.5539 | FALSE |
| c6 | Female KO vs Male KO | -3.6494 | 0.0000 | TRUE |

## 4.9 ECG

**Number of animals**

| STRAIN | GENDER | count |
| --- | --- | --- |
| AMB-YLF-GGG | female | 8 |
| AMB-YLF-GGG | male | 8 |
| C57Bl/6NCrl | female | 864 |
| C57Bl/6NCrl | male | 870 |

### 4.9.1 Heart rate

|  | test | Difference | p\_value | Significance |
| --- | --- | --- | --- | --- |
| c2 | CTRl vs KO | -17.4215 | 0.6426 | FALSE |
| c3 | Female vs Male | 1.6170 | 0.9978 | FALSE |
| c4 | Female CTRL vs Female KO | -17.1635 | 0.7526 | FALSE |
| c5 | Male CTRL vs Male KO | -17.6795 | 0.7347 | FALSE |
| c6 | Female KO vs Male KO | 1.3590 | 0.8594 | FALSE |

### 4.9.2 HRV

|  | test | Difference | p\_value | Significance |
| --- | --- | --- | --- | --- |
| c2 | CTRl vs KO | 5.6636 | 0.5089 | FALSE |
| c3 | Female vs Male | 2.1309 | 0.8437 | FALSE |
| c4 | Female CTRL vs Female KO | 4.3952 | 0.7993 | FALSE |
| c5 | Male CTRL vs Male KO | 6.9320 | 0.4806 | FALSE |
| c6 | Female KO vs Male KO | 3.3993 | 0.0000 | TRUE |

### 4.9.3 Mean R amplitude

|  | test | Difference | p\_value | Significance |
| --- | --- | --- | --- | --- |
| c2 | CTRl vs KO | -0.0414 | 0.9675 | FALSE |
| c3 | Female vs Male | 0.0776 | 0.4953 | FALSE |
| c4 | Female CTRL vs Female KO | -0.0040 | 1.0000 | FALSE |
| c5 | Male CTRL vs Male KO | -0.0787 | 0.8777 | FALSE |
| c6 | Female KO vs Male KO | 0.0403 | 0.0015 | TRUE |

### 4.9.4 Mean SR amplitude

|  | test | Difference | p\_value | Significance |
| --- | --- | --- | --- | --- |
| c2 | CTRl vs KO | 0.0460 | 0.9800 | FALSE |
| c3 | Female vs Male | 0.0932 | 0.5721 | FALSE |
| c4 | Female CTRL vs Female KO | 0.0915 | 0.9121 | FALSE |
| c5 | Male CTRL vs Male KO | 0.0004 | 1.0000 | FALSE |
| c6 | Female KO vs Male KO | 0.0477 | 0.0053 | TRUE |

### 4.9.5 PQ

|  | test | Difference | p\_value | Significance |
| --- | --- | --- | --- | --- |
| c2 | CTRl vs KO | 0.5109 | 0.9353 | FALSE |
| c3 | Female vs Male | 0.5156 | 0.7466 | FALSE |
| c4 | Female CTRL vs Female KO | 1.1578 | 0.6617 | FALSE |
| c5 | Male CTRL vs Male KO | -0.1361 | 0.9991 | FALSE |
| c6 | Female KO vs Male KO | -0.1314 | 0.5558 | FALSE |

### 4.9.6 PR

|  | test | Difference | p\_value | Significance |
| --- | --- | --- | --- | --- |
| c2 | CTRl vs KO | 0.8283 | 0.7946 | FALSE |
| c3 | Female vs Male | 0.6279 | 0.6626 | FALSE |
| c4 | Female CTRL vs Female KO | 1.5379 | 0.4685 | FALSE |
| c5 | Male CTRL vs Male KO | 0.1187 | 0.9995 | FALSE |
| c6 | Female KO vs Male KO | -0.0817 | 0.8692 | FALSE |

### 4.9.7 QRS

|  | test | Difference | p\_value | Significance |
| --- | --- | --- | --- | --- |
| c2 | CTRl vs KO | -0.0044 | 1.0000 | FALSE |
| c3 | Female vs Male | 0.0751 | 0.9786 | FALSE |
| c4 | Female CTRL vs Female KO | 0.0581 | 0.9973 | FALSE |
| c5 | Male CTRL vs Male KO | -0.0668 | 0.9958 | FALSE |
| c6 | Female KO vs Male KO | 0.0126 | 0.9855 | FALSE |

### 4.9.8 QT

|  | test | Difference | p\_value | Significance |
| --- | --- | --- | --- | --- |
| c2 | CTRl vs KO | 0.7948 | 0.7917 | FALSE |
| c3 | Female vs Male | -0.4902 | 0.8223 | FALSE |
| c4 | Female CTRL vs Female KO | 0.5475 | 0.9511 | FALSE |
| c5 | Male CTRL vs Male KO | 1.0421 | 0.7423 | FALSE |
| c6 | Female KO vs Male KO | -0.2429 | 0.1294 | FALSE |

### 4.9.9 QT\_dispersion

|  | test | Difference | p\_value | Significance |
| --- | --- | --- | --- | --- |
| c2 | CTRl vs KO | 2.8596 | 0.4940 | FALSE |
| c3 | Female vs Male | 0.9792 | 0.7438 | FALSE |
| c4 | Female CTRL vs Female KO | 3.1930 | 0.4889 | FALSE |
| c5 | Male CTRL vs Male KO | 2.5263 | 0.6732 | FALSE |
| c6 | Female KO vs Male KO | 0.6458 | 0.0056 | TRUE |

### 4.9.10 QTc

|  | test | Difference | p\_value | Significance |
| --- | --- | --- | --- | --- |
| c2 | CTRl vs KO | 0.2888 | 0.9698 | FALSE |
| c3 | Female vs Male | -0.5494 | 0.6301 | FALSE |
| c4 | Female CTRL vs Female KO | -0.0118 | 1.0000 | FALSE |
| c5 | Male CTRL vs Male KO | 0.5894 | 0.8782 | FALSE |
| c6 | Female KO vs Male KO | -0.2488 | 0.0313 | TRUE |

### 4.9.11 QTc Dispersion

|  | test | Difference | p\_value | Significance |
| --- | --- | --- | --- | --- |
| c2 | CTRl vs KO | 2.7644 | 0.5199 | FALSE |
| c3 | Female vs Male | 0.9379 | 0.7573 | FALSE |
| c4 | Female CTRL vs Female KO | 3.0516 | 0.5222 | FALSE |
| c5 | Male CTRL vs Male KO | 2.4773 | 0.6820 | FALSE |
| c6 | Female KO vs Male KO | 0.6507 | 0.0041 | TRUE |

### 4.9.12 RR

|  | test | Difference | p\_value | Significance |
| --- | --- | --- | --- | --- |
| c2 | CTRl vs KO | 1.9314 | 0.6725 | FALSE |
| c3 | Female vs Male | -0.0331 | 1.0000 | FALSE |
| c4 | Female CTRL vs Female KO | 1.9770 | 0.7578 | FALSE |
| c5 | Male CTRL vs Male KO | 1.8857 | 0.7826 | FALSE |
| c6 | Female KO vs Male KO | -0.0788 | 0.9801 | FALSE |

## 4.10 Lung screen

**Number of animals**

| STRAIN | GENDER | count |
| --- | --- | --- |
| AMB-YLF-GGG | female | 8 |
| AMB-YLF-GGG | male | 8 |
| C57Bl/6NCrl | female | 941 |
| C57Bl/6NCrl | male | 943 |

### 4.10.1 Compliance

|  | test | Difference | p\_value | Significance |
| --- | --- | --- | --- | --- |
| c2 | CTRl vs KO | -0.0038 | 0.2119 | FALSE |
| c3 | Female vs Male | 0.0011 | 0.6001 | FALSE |
| c4 | Female CTRL vs Female KO | -0.0055 | 0.0546 | FALSE |
| c5 | Male CTRL vs Male KO | -0.0021 | 0.7675 | FALSE |
| c6 | Female KO vs Male KO | 0.0029 | 0.0000 | TRUE |

### 4.10.2 Elastance

|  | test | Difference | p\_value | Significance |
| --- | --- | --- | --- | --- |
| c2 | CTRl vs KO | 3.1561 | 0.0618 | FALSE |
| c3 | Female vs Male | 0.0410 | 0.9999 | FALSE |
| c4 | Female CTRL vs Female KO | 5.0608 | 0.0020 | TRUE |
| c5 | Male CTRL vs Male KO | 1.2515 | 0.7958 | FALSE |
| c6 | Female KO vs Male KO | -1.8637 | 0.0000 | TRUE |

### 4.10.3 Inspiratory Capacity

|  | test | Difference | p\_value | Significance |
| --- | --- | --- | --- | --- |
| c2 | CTRl vs KO | -0.0623 | 0.1217 | FALSE |
| c3 | Female vs Male | 0.0371 | 0.0692 | FALSE |
| c4 | Female CTRL vs Female KO | -0.0895 | 0.0279 | TRUE |
| c5 | Male CTRL vs Male KO | -0.0351 | 0.6777 | FALSE |
| c6 | Female KO vs Male KO | 0.0643 | 0.0000 | TRUE |

### 4.10.4 Resistance

|  | test | Difference | p\_value | Significance |
| --- | --- | --- | --- | --- |
| c2 | CTRl vs KO | 0.0418 | 0.7056 | FALSE |
| c3 | Female vs Male | 0.0092 | 0.9534 | FALSE |
| c4 | Female CTRL vs Female KO | 0.0822 | 0.2254 | FALSE |
| c5 | Male CTRL vs Male KO | 0.0015 | 1.0000 | FALSE |
| c6 | Female KO vs Male KO | -0.0312 | 0.0000 | TRUE |

### 4.10.5 Resistance of the airways (Prime8)

|  | test | Difference | p\_value | Significance |
| --- | --- | --- | --- | --- |
| c2 | CTRl vs KO | 0.0207 | 0.8589 | FALSE |
| c3 | Female vs Male | 0.0034 | 0.9935 | FALSE |
| c4 | Female CTRL vs Female KO | 0.0338 | 0.6519 | FALSE |
| c5 | Male CTRL vs Male KO | 0.0076 | 0.9936 | FALSE |
| c6 | Female KO vs Male KO | -0.0097 | 0.0862 | FALSE |

### 4.10.6 Resistance of the airways (QuickPrime3)

|  | test | Difference | p\_value | Significance |
| --- | --- | --- | --- | --- |
| c2 | CTRl vs KO | 0.0094 | 0.9882 | FALSE |
| c3 | Female vs Male | -0.0069 | 0.9641 | FALSE |
| c4 | Female CTRL vs Female KO | 0.0182 | 0.9424 | FALSE |
| c5 | Male CTRL vs Male KO | 0.0006 | 1.0000 | FALSE |
| c6 | Female KO vs Male KO | -0.0157 | 0.0049 | TRUE |

### 4.10.7 Tissue damping (Prime8)

|  | test | Difference | p\_value | Significance |
| --- | --- | --- | --- | --- |
| c2 | CTRl vs KO | 0.2073 | 0.7966 | FALSE |
| c3 | Female vs Male | 0.0313 | 0.9930 | FALSE |
| c4 | Female CTRL vs Female KO | 0.5531 | 0.1381 | FALSE |
| c5 | Male CTRL vs Male KO | -0.1385 | 0.9470 | FALSE |
| c6 | Female KO vs Male KO | -0.3145 | 0.0000 | TRUE |

### 4.10.8 Tissue damping (QuickPrime3)

|  | test | Difference | p\_value | Significance |
| --- | --- | --- | --- | --- |
| c2 | CTRl vs KO | 0.3571 | 0.3279 | FALSE |
| c3 | Female vs Male | 0.0289 | 0.9921 | FALSE |
| c4 | Female CTRL vs Female KO | 0.6426 | 0.0350 | TRUE |
| c5 | Male CTRL vs Male KO | 0.0716 | 0.9897 | FALSE |
| c6 | Female KO vs Male KO | -0.2566 | 0.0000 | TRUE |

### 4.10.9 Tissue elastance (Prime8)

|  | test | Difference | p\_value | Significance |
| --- | --- | --- | --- | --- |
| c2 | CTRl vs KO | 3.1637 | 0.0749 | FALSE |
| c3 | Female vs Male | -0.1608 | 0.9929 | FALSE |
| c4 | Female CTRL vs Female KO | 5.2618 | 0.0020 | TRUE |
| c5 | Male CTRL vs Male KO | 1.0655 | 0.8730 | FALSE |
| c6 | Female KO vs Male KO | -2.2590 | 0.0000 | TRUE |

### 4.10.10 Tissue elastance (QuickPrime3)

|  | test | Difference | p\_value | Significance |
| --- | --- | --- | --- | --- |
| c2 | CTRl vs KO | 3.0564 | 0.0578 | FALSE |
| c3 | Female vs Male | -0.1176 | 0.9965 | FALSE |
| c4 | Female CTRL vs Female KO | 4.9776 | 0.0016 | TRUE |
| c5 | Male CTRL vs Male KO | 1.1351 | 0.8198 | FALSE |
| c6 | Female KO vs Male KO | -2.0389 | 0.0000 | TRUE |

## 4.11 Intraperitoneal Glucose Tolerance Test

**Number of animals**

| STRAIN | GENDER | count |
| --- | --- | --- |
| AMB-YLF-GGG | female | 6 |
| AMB-YLF-GGG | male | 6 |
| C57Bl/6NCrl | female | 336 |
| C57Bl/6NCrl | male | 345 |

|  | test | Difference | p\_value | Significance |
| --- | --- | --- | --- | --- |
| c2 | CTRl vs KO | -2.8913 | 0.9925 | FALSE |
| c3 | Female vs Male | -11.0943 | 0.7166 | FALSE |
| c4 | Female CTRL vs Female KO | -1.0973 | 0.9999 | FALSE |
| c5 | Male CTRL vs Male KO | -4.6852 | 0.9858 | FALSE |
| c6 | Female KO vs Male KO | -12.8882 | 0.0013 | TRUE |

## 4.12 Auditory Brain Stem Response

**Number of animals**

| STRAIN | GENDER | count |
| --- | --- | --- |
| AMB-YLF-GGG | female | 8 |
| AMB-YLF-GGG | male | 7 |
| C57Bl/6NCrl | female | 894 |
| C57Bl/6NCrl | male | 872 |

### 4.12.1 Click-evoked ABR threshold

|  | test | Difference | p\_value | Significance |
| --- | --- | --- | --- | --- |
| c2 | CTRl vs KO | 0.1783 | 0.9997 | FALSE |
| c3 | Female vs Male | 0.9578 | 0.7864 | FALSE |
| c4 | Female CTRL vs Female KO | 0.4224 | 0.9975 | FALSE |
| c5 | Male CTRL vs Male KO | -0.0659 | 1.0000 | FALSE |
| c6 | Female KO vs Male KO | 0.7137 | 0.0022 | TRUE |

### 4.12.2 6kHz-evoked ABR Threshold

|  | test | Difference | p\_value | Significance |
| --- | --- | --- | --- | --- |
| c2 | CTRl vs KO | 1.1319 | 0.9732 | FALSE |
| c3 | Female vs Male | -0.7213 | 0.9732 | FALSE |
| c4 | Female CTRL vs Female KO | 0.2716 | 0.9998 | FALSE |
| c5 | Male CTRL vs Male KO | 1.9922 | 0.9065 | FALSE |
| c6 | Female KO vs Male KO | 0.1391 | 0.9711 | FALSE |

### 4.12.3 12kHz-evoked ABR Threshold

|  | test | Difference | p\_value | Significance |
| --- | --- | --- | --- | --- |
| c2 | CTRl vs KO | -4.0934 | 0.4083 | FALSE |
| c3 | Female vs Male | -1.9090 | 0.6791 | FALSE |
| c4 | Female CTRL vs Female KO | -4.7832 | 0.4686 | FALSE |
| c5 | Male CTRL vs Male KO | -3.4036 | 0.6625 | FALSE |
| c6 | Female KO vs Male KO | -1.2192 | 0.0013 | TRUE |

### 4.12.4 18kHz-evoked ABR Threshold

|  | test | Difference | p\_value | Significance |
| --- | --- | --- | --- | --- |
| c2 | CTRl vs KO | -0.4369 | 0.9995 | FALSE |
| c3 | Female vs Male | -1.1493 | 0.9636 | FALSE |
| c4 | Female CTRL vs Female KO | -2.0562 | 0.9733 | FALSE |
| c5 | Male CTRL vs Male KO | 1.1823 | 0.9926 | FALSE |
| c6 | Female KO vs Male KO | 0.4699 | 0.7271 | FALSE |

### 4.12.5 24kHz-evoked ABR Threshold

|  | test | Difference | p\_value | Significance |
| --- | --- | --- | --- | --- |
| c2 | CTRl vs KO | -6.1973 | 0.8219 | FALSE |
| c3 | Female vs Male | 2.2687 | 0.9356 | FALSE |
| c4 | Female CTRL vs Female KO | -5.9294 | 0.9028 | FALSE |
| c5 | Male CTRL vs Male KO | -6.4653 | 0.8268 | FALSE |
| c6 | Female KO vs Male KO | 2.0007 | 0.0418 | TRUE |

### 4.12.6 30kHz-evoked ABR Threshold

|  | test | Difference | p\_value | Significance |
| --- | --- | --- | --- | --- |
| c2 | CTRl vs KO | -10.7585 | 0.4182 | FALSE |
| c3 | Female vs Male | 0.0838 | 1.0000 | FALSE |
| c4 | Female CTRL vs Female KO | -14.7677 | 0.3385 | FALSE |
| c5 | Male CTRL vs Male KO | -6.7493 | 0.8315 | FALSE |
| c6 | Female KO vs Male KO | 4.0930 | 0.0007 | TRUE |

```
Error in `[.data.frame`(IMPC_DXA_001, , c("SPECIMEN_ID", "EXPERIMENT_ID", : undefined columns selected
```

```
Error in rbind(deparse.level, ...): object 'df2' not found
```

## 4.13 Skeleton morfology

**Number of animals**

| STRAIN | GENDER | count |
| --- | --- | --- |
| AMB-YLF-GGG | female | 8 |
| AMB-YLF-GGG | male | 7 |
| C57Bl/6NCrl | female | 914 |
| C57Bl/6NCrl | male | 899 |

### 4.13.1 Tibia length

|  | test | Difference | p\_value | Significance |
| --- | --- | --- | --- | --- |
| c2 | CTRl vs KO | -0.8134 | 0.0577 | FALSE |
| c3 | Female vs Male | -0.5876 | 0.0180 | TRUE |
| c4 | Female CTRL vs Female KO | -1.1479 | 0.0231 | TRUE |
| c5 | Male CTRL vs Male KO | -0.4788 | 0.5221 | FALSE |
| c6 | Female KO vs Male KO | -0.2531 | 0.0012 | TRUE |

### 4.13.2 Number of caudal vertebrae

|  | 23:24 | 23:25 | 23:26 | 23:27 | 23:28 | 23:29 | 23:30 | 24:25 | 24:26 | 24:27 | 24:28 | 24:29 | 24:30 | 25:26 | 25:27 | 25:28 | 25:29 | 25:30 | 26:27 | 26:28 | 26:29 | 26:30 | 27:28 | 27:29 | 27:30 | 28:29 | 28:30 | 29:30 |
| --- | --- | --- | --- | --- | --- | --- | --- | --- | --- | --- | --- | --- | --- | --- | --- | --- | --- | --- | --- | --- | --- | --- | --- | --- | --- | --- | --- | --- |
| female AMB-YLF-GGG:female C57Bl/6NCrl | 1 | 1 | 1 | 1 | 1 | 1 | 1 | 1 | 1 | 1 | 1 | 1 | 1 | 1 | 1 | 1 | 1 | 1 | 1 | 1 | 1 | 1 | 1 | 1 | 1 | 1 | 1 | 1 |
| female AMB-YLF-GGG:male AMB-YLF-GGG | 1 | 1 | 1 | 1 | 1 | 1 | 1 | 1 | 1 | 1 | 1 | 1 | 1 | 1 | 1 | 1 | 1 | 1 | 1 | 1 | 1 | 1 | 1 | 1 | 1 | 1 | 1 | 1 |
| female AMB-YLF-GGG:male C57Bl/6NCrl | 1 | 1 | 1 | 1 | 1 | 1 | 1 | 1 | 1 | 1 | 1 | 1 | 1 | 1 | 1 | 1 | 1 | 1 | 1 | 1 | 1 | 1 | 1 | 1 | 1 | 1 | 1 | 1 |
| female C57Bl/6NCrl:male AMB-YLF-GGG | 1 | 1 | 1 | 1 | 1 | 1 | 1 | 1 | 1 | 1 | 1 | 1 | 1 | 1 | 1 | 1 | 1 | 1 | 1 | 1 | 1 | 1 | 1 | 1 | 1 | 1 | 1 | 1 |
| female C57Bl/6NCrl:male C57Bl/6NCrl | 1 | 1 | 1 | 1 | 1 | 1 | 1 | 1 | 1 | 1 | 1 | 1 | 1 | 1 | 1 | 1 | 1 | 1 | 1 | 1 | 1 | 1 | 1 | 1 | 1 | 1 | 1 | 1 |
| male AMB-YLF-GGG:male C57Bl/6NCrl | 1 | 1 | 1 | 1 | 1 | 1 | 1 | 1 | 1 | 1 | 1 | 1 | 1 | 1 | 1 | 1 | 1 | 1 | 1 | 1 | 1 | 1 | 1 | 1 | 1 | 1 | 1 | 1 |

### 4.13.3 Number of cervical vertebrae

### 4.13.4 Number of lumbar vertebrae

|  | female AMB-YLF-GGG | female C57Bl/6NCrl | male AMB-YLF-GGG |
| --- | --- | --- | --- |
| female C57Bl/6NCrl | 1 | NA | NA |
| male AMB-YLF-GGG | 1 | 1.0000 | NA |
| male C57Bl/6NCrl | 1 | 0.3615 | 1 |

### 4.13.5 Number of pelvic vertebrae

|  | 4:5 | 4:6 | 5:6 |
| --- | --- | --- | --- |
| female AMB-YLF-GGG:female C57Bl/6NCrl | 1 | 1 | 1 |
| female AMB-YLF-GGG:male AMB-YLF-GGG | 1 | 1 | 1 |
| female AMB-YLF-GGG:male C57Bl/6NCrl | 1 | 1 | 1 |
| female C57Bl/6NCrl:male AMB-YLF-GGG | 1 | 1 | 1 |
| female C57Bl/6NCrl:male C57Bl/6NCrl | 1 | 1 | 1 |
| male AMB-YLF-GGG:male C57Bl/6NCrl | 1 | 1 | 1 |

### 4.13.6 Number of thoracic vertebrae

### 4.13.7 Number of digits

|  | female AMB-YLF-GGG | female C57Bl/6NCrl | male AMB-YLF-GGG |
| --- | --- | --- | --- |
| female C57Bl/6NCrl | 0.0000 | NA | NA |
| male AMB-YLF-GGG | 0.0028 | 1.0000 | NA |
| male C57Bl/6NCrl | 0.0000 | 0.5553 | 1 |

### 4.13.8 Number of digits in forepaws

### 4.13.9 Number of digits lo hindpaws

|  | female AMB-YLF-GGG | female C57Bl/6NCrl | male AMB-YLF-GGG |
| --- | --- | --- | --- |
| female C57Bl/6NCrl | 1 | NA | NA |
| male AMB-YLF-GGG | 1 | 1 | NA |
| male C57Bl/6NCrl | 1 | 1 | 1 |

### 4.13.10 Number of ribs left

|  | 12:13 | 12:14 | 13:14 |
| --- | --- | --- | --- |
| female AMB-YLF-GGG:female C57Bl/6NCrl | 1 | 1 | 1 |
| female AMB-YLF-GGG:male AMB-YLF-GGG | 1 | 1 | 1 |
| female AMB-YLF-GGG:male C57Bl/6NCrl | 1 | 1 | 1 |
| female C57Bl/6NCrl:male AMB-YLF-GGG | 1 | 1 | 1 |
| female C57Bl/6NCrl:male C57Bl/6NCrl | 1 | 1 | 1 |
| male AMB-YLF-GGG:male C57Bl/6NCrl | 1 | 1 | 1 |

### 4.13.11 Number of ribs right

|  | female AMB-YLF-GGG | female C57Bl/6NCrl | male AMB-YLF-GGG |
| --- | --- | --- | --- |
| female C57Bl/6NCrl | 1 | NA | NA |
| male AMB-YLF-GGG | 1 | 1 | NA |
| male C57Bl/6NCrl | 1 | 1 | 1 |

### 4.13.12 Brachydactyly

|  | no:unobservable | no:yes | unobservable:yes |
| --- | --- | --- | --- |
| female AMB-YLF-GGG:female C57Bl/6NCrl | 1 | 0.0795 | 1 |
| female AMB-YLF-GGG:male AMB-YLF-GGG | 1 | 1.0000 | 1 |
| female AMB-YLF-GGG:male C57Bl/6NCrl | 1 | 0.0795 | 1 |
| female C57Bl/6NCrl:male AMB-YLF-GGG | 1 | 1.0000 | 1 |
| female C57Bl/6NCrl:male C57Bl/6NCrl | 1 | 1.0000 | 1 |
| male AMB-YLF-GGG:male C57Bl/6NCrl | 1 | 1.0000 | 1 |

### 4.13.13 Caudal processes

|  | abnormal:normal | abnormal:unobservable | normal:unobservable |
| --- | --- | --- | --- |
| female AMB-YLF-GGG:female C57Bl/6NCrl | 1 | 1 | 1 |
| female AMB-YLF-GGG:male AMB-YLF-GGG | 1 | 1 | 1 |
| female AMB-YLF-GGG:male C57Bl/6NCrl | 1 | 1 | 1 |
| female C57Bl/6NCrl:male AMB-YLF-GGG | 1 | 1 | 1 |
| female C57Bl/6NCrl:male C57Bl/6NCrl | 1 | 1 | 1 |
| male AMB-YLF-GGG:male C57Bl/6NCrl | 1 | 1 | 1 |

### 4.13.14 Cervical processes

|  | female AMB-YLF-GGG | female C57Bl/6NCrl | male AMB-YLF-GGG |
| --- | --- | --- | --- |
| female C57Bl/6NCrl | 1.0 | NA | NA |
| male AMB-YLF-GGG | 0.7 | 0.5657 | NA |
| male C57Bl/6NCrl | 1.0 | 0.7000 | 0.5657 |

### 4.13.15 Clavicle

### 4.13.16 Digit integrity

|  | no:unobservable | no:yes | unobservable:yes |
| --- | --- | --- | --- |
| female AMB-YLF-GGG:female C57Bl/6NCrl | 1 | 0.253 | 1 |
| female AMB-YLF-GGG:male AMB-YLF-GGG | 1 | 1.000 | 1 |
| female AMB-YLF-GGG:male C57Bl/6NCrl | 1 | 0.253 | 1 |
| female C57Bl/6NCrl:male AMB-YLF-GGG | 1 | 0.253 | 1 |
| female C57Bl/6NCrl:male C57Bl/6NCrl | 1 | 1.000 | 1 |
| male AMB-YLF-GGG:male C57Bl/6NCrl | 1 | 0.253 | 1 |

### 4.13.17 Femur

|  | abnormal:normal | abnormal:unobservable | normal:unobservable |
| --- | --- | --- | --- |
| female AMB-YLF-GGG:female C57Bl/6NCrl | 1 | 1 | 1 |
| female AMB-YLF-GGG:male AMB-YLF-GGG | 1 | 1 | 1 |
| female AMB-YLF-GGG:male C57Bl/6NCrl | 1 | 1 | 1 |
| female C57Bl/6NCrl:male AMB-YLF-GGG | 1 | 1 | 1 |
| female C57Bl/6NCrl:male C57Bl/6NCrl | 1 | 1 | 1 |
| male AMB-YLF-GGG:male C57Bl/6NCrl | 1 | 1 | 1 |

### 4.13.18 Fibula

|  | female AMB-YLF-GGG | female C57Bl/6NCrl | male AMB-YLF-GGG |
| --- | --- | --- | --- |
| female C57Bl/6NCrl | 1 | NA | NA |
| male AMB-YLF-GGG | 1 | 1 | NA |
| male C57Bl/6NCrl | 1 | 1 | 1 |

### 4.13.19 Fusion of processes

|  | female AMB-YLF-GGG | female C57Bl/6NCrl | male AMB-YLF-GGG |
| --- | --- | --- | --- |
| female C57Bl/6NCrl | 1 | NA | NA |
| male AMB-YLF-GGG | 1 | 1 | NA |
| male C57Bl/6NCrl | 1 | 1 | 1 |

### 4.13.20 Fusion of ribs

|  | female AMB-YLF-GGG | female C57Bl/6NCrl | male AMB-YLF-GGG |
| --- | --- | --- | --- |
| female C57Bl/6NCrl | 1 | NA | NA |
| male AMB-YLF-GGG | 1 | 1 | NA |
| male C57Bl/6NCrl | 1 | 1 | 1 |

### 4.13.21 Fusion of vertebrae

|  | female AMB-YLF-GGG | female C57Bl/6NCrl | male AMB-YLF-GGG |
| --- | --- | --- | --- |
| female C57Bl/6NCrl | 1 | NA | NA |
| male AMB-YLF-GGG | 1 | 1 | NA |
| male C57Bl/6NCrl | 1 | 1 | 1 |

### 4.13.22 Humerus

### 4.13.23 Joints

|  | abnormal:normal | abnormal:unobservable | normal:unobservable |
| --- | --- | --- | --- |
| female AMB-YLF-GGG:female C57Bl/6NCrl | 1.0000 | 1 | 1 |
| female AMB-YLF-GGG:male AMB-YLF-GGG | 0.9000 | 1 | 1 |
| female AMB-YLF-GGG:male C57Bl/6NCrl | 1.0000 | 1 | 1 |
| female C57Bl/6NCrl:male AMB-YLF-GGG | 0.6193 | 1 | 1 |
| female C57Bl/6NCrl:male C57Bl/6NCrl | 0.3152 | 1 | 1 |
| male AMB-YLF-GGG:male C57Bl/6NCrl | 0.8055 | 1 | 1 |

### 4.13.24 Kyphosis

|  | female AMB-YLF-GGG | female C57Bl/6NCrl | male AMB-YLF-GGG |
| --- | --- | --- | --- |
| female C57Bl/6NCrl | 1 | NA | NA |
| male AMB-YLF-GGG | 1 | 1.0000 | NA |
| male C57Bl/6NCrl | 1 | 0.0229 | 1 |

### 4.13.25 Lordosis

|  | female AMB-YLF-GGG | female C57Bl/6NCrl | male AMB-YLF-GGG |
| --- | --- | --- | --- |
| female C57Bl/6NCrl | 1 | NA | NA |
| male AMB-YLF-GGG | 1 | 1 | NA |
| male C57Bl/6NCrl | 1 | 1 | 1 |

### 4.13.26 Lumbar processes

|  | female AMB-YLF-GGG | female C57Bl/6NCrl | male AMB-YLF-GGG |
| --- | --- | --- | --- |
| female C57Bl/6NCrl | 1 | NA | NA |
| male AMB-YLF-GGG | 1 | 1e+00 | NA |
| male C57Bl/6NCrl | 1 | 1e-04 | 1 |

### 4.13.27 Mandibles

### 4.13.28 Maxilla/Pre-maxilla

|  | female AMB-YLF-GGG | female C57Bl/6NCrl | male AMB-YLF-GGG |
| --- | --- | --- | --- |
| female C57Bl/6NCrl | 1 | NA | NA |
| male AMB-YLF-GGG | 1 | 1 | NA |
| male C57Bl/6NCrl | 1 | 1 | 1 |

### 4.13.29 Missing cranial rib

### 4.13.30 Pelvis

|  | female AMB-YLF-GGG | female C57Bl/6NCrl | male AMB-YLF-GGG |
| --- | --- | --- | --- |
| female C57Bl/6NCrl | 0.0001 | NA | NA |
| male AMB-YLF-GGG | 0.0385 | 1.0000 | NA |
| male C57Bl/6NCrl | 0.0000 | 0.0033 | 1 |

### 4.13.31 Polysyndactylism

|  | no:unobservable | no:yes | unobservable:yes |
| --- | --- | --- | --- |
| female AMB-YLF-GGG:female C57Bl/6NCrl | 1 | 1 | 1 |
| female AMB-YLF-GGG:male AMB-YLF-GGG | 1 | 1 | 1 |
| female AMB-YLF-GGG:male C57Bl/6NCrl | 1 | 1 | 1 |
| female C57Bl/6NCrl:male AMB-YLF-GGG | 1 | 1 | 1 |
| female C57Bl/6NCrl:male C57Bl/6NCrl | 1 | 1 | 1 |
| male AMB-YLF-GGG:male C57Bl/6NCrl | 1 | 1 | 1 |

### 4.13.32 Presence of baculum bone

|  | no:unobservable | no:yes | unobservable:yes |
| --- | --- | --- | --- |
| female AMB-YLF-GGG:female C57Bl/6NCrl | 1 | 0.2485 | 0.2648 |
| female AMB-YLF-GGG:male AMB-YLF-GGG | 1 | 1.0000 | 1.0000 |
| female AMB-YLF-GGG:male C57Bl/6NCrl | 1 | 0.2343 | 1.0000 |
| female C57Bl/6NCrl:male AMB-YLF-GGG | 1 | 0.0915 | 0.2648 |
| female C57Bl/6NCrl:male C57Bl/6NCrl | 1 | 0.0000 | 0.0000 |
| male AMB-YLF-GGG:male C57Bl/6NCrl | 1 | 1.0000 | 1.0000 |

### 4.13.33 Processes on vertebrae

|  | female AMB-YLF-GGG | female C57Bl/6NCrl | male AMB-YLF-GGG |
| --- | --- | --- | --- |
| female C57Bl/6NCrl | 1 | NA | NA |
| male AMB-YLF-GGG | 1 | 1.0000 | NA |
| male C57Bl/6NCrl | 1 | 0.0638 | 1 |

### 4.13.34 Quality of enamel on incisors

|  | female AMB-YLF-GGG | female C57Bl/6NCrl | male AMB-YLF-GGG |
| --- | --- | --- | --- |
| female C57Bl/6NCrl | 0.0000 | NA | NA |
| male AMB-YLF-GGG | 0.6084 | 0.0000 | NA |
| male C57Bl/6NCrl | 0.0000 | 0.1446 | 0 |

### 4.13.35 Quality of enamel on molars

|  | female AMB-YLF-GGG | female C57Bl/6NCrl | male AMB-YLF-GGG |
| --- | --- | --- | --- |
| female C57Bl/6NCrl | 0.0002 | NA | NA |
| male AMB-YLF-GGG | 0.9333 | 1 | NA |
| male C57Bl/6NCrl | 0.0002 | 1 | 1 |

### 4.13.36 Radius

### 4.13.37 Sacral processes

|  | female AMB-YLF-GGG | female C57Bl/6NCrl | male AMB-YLF-GGG |
| --- | --- | --- | --- |
| female C57Bl/6NCrl | 1 | NA | NA |
| male AMB-YLF-GGG | 1 | 1 | NA |
| male C57Bl/6NCrl | 1 | 1 | 1 |

### 4.13.38 Scapulae

### 4.13.39 Scoliosis

|  | female AMB-YLF-GGG | female C57Bl/6NCrl | male AMB-YLF-GGG |
| --- | --- | --- | --- |
| female C57Bl/6NCrl | 0.6511 | NA | NA |
| male AMB-YLF-GGG | 1.0000 | 0.6511 | NA |
| male C57Bl/6NCrl | 0.6511 | 0.1050 | 0.6511 |

### 4.13.40 Shape of ribcage

|  | female AMB-YLF-GGG | female C57Bl/6NCrl | male AMB-YLF-GGG |
| --- | --- | --- | --- |
| female C57Bl/6NCrl | 1.0 | NA | NA |
| male AMB-YLF-GGG | 0.3 | 0.0379 | NA |
| male C57Bl/6NCrl | 1.0 | 0.0000 | 0.2315 |

### 4.13.41 Shape of ribs

|  | female AMB-YLF-GGG | female C57Bl/6NCrl | male AMB-YLF-GGG |
| --- | --- | --- | --- |
| female C57Bl/6NCrl | 1 | NA | NA |
| male AMB-YLF-GGG | 1 | 1 | NA |
| male C57Bl/6NCrl | 1 | 1 | 1 |

### 4.13.42 Shape of spine

|  | female AMB-YLF-GGG | female C57Bl/6NCrl | male AMB-YLF-GGG |
| --- | --- | --- | --- |
| female C57Bl/6NCrl | 1 | NA | NA |
| male AMB-YLF-GGG | 1 | 1.0000 | NA |
| male C57Bl/6NCrl | 1 | 0.0021 | 1 |

### 4.13.43 Shape of vertebrae

|  | female AMB-YLF-GGG | female C57Bl/6NCrl | male AMB-YLF-GGG |
| --- | --- | --- | --- |
| female C57Bl/6NCrl | 1.0 | NA | NA |
| male AMB-YLF-GGG | 0.7 | 0.4085 | NA |
| male C57Bl/6NCrl | 1.0 | 0.4085 | 0.4085 |

### 4.13.44 Skull shape

|  | female AMB-YLF-GGG | female C57Bl/6NCrl | male AMB-YLF-GGG |
| --- | --- | --- | --- |
| female C57Bl/6NCrl | 0.0003 | NA | NA |
| male AMB-YLF-GGG | 0.0608 | 1e+00 | NA |
| male C57Bl/6NCrl | 0.0000 | 3e-04 | 0.5009 |

### 4.13.45 Syndactylism

|  | no syndactylism:Syndactylism | no syndactylism:unobservable | Syndactylism:unobservable |
| --- | --- | --- | --- |
| female AMB-YLF-GGG:female C57Bl/6NCrl | 1 | 1 | 1 |
| female AMB-YLF-GGG:male AMB-YLF-GGG | 1 | 1 | 1 |
| female AMB-YLF-GGG:male C57Bl/6NCrl | 1 | 1 | 1 |
| female C57Bl/6NCrl:male AMB-YLF-GGG | 1 | 1 | 1 |
| female C57Bl/6NCrl:male C57Bl/6NCrl | 1 | 1 | 1 |
| male AMB-YLF-GGG:male C57Bl/6NCrl | 1 | 1 | 1 |

### 4.13.46 Teeth

|  | female AMB-YLF-GGG | female C57Bl/6NCrl | male AMB-YLF-GGG |
| --- | --- | --- | --- |
| female C57Bl/6NCrl | 0.0000 | NA | NA |
| male AMB-YLF-GGG | 0.4667 | 0.0000 | NA |
| male C57Bl/6NCrl | 0.0000 | 0.4667 | 0 |

### 4.13.47 Thoracic processes

|  | female AMB-YLF-GGG | female C57Bl/6NCrl | male AMB-YLF-GGG |
| --- | --- | --- | --- |
| female C57Bl/6NCrl | 1 | NA | NA |
| male AMB-YLF-GGG | 1 | 1 | NA |
| male C57Bl/6NCrl | 1 | 1 | 1 |

### 4.13.48 Tibia

|  | female AMB-YLF-GGG | female C57Bl/6NCrl | male AMB-YLF-GGG |
| --- | --- | --- | --- |
| female C57Bl/6NCrl | 1 | NA | NA |
| male AMB-YLF-GGG | 1 | 1 | NA |
| male C57Bl/6NCrl | 1 | 1 | 1 |

### 4.13.49 Transitional vertebrae

|  | female AMB-YLF-GGG | female C57Bl/6NCrl | male AMB-YLF-GGG |
| --- | --- | --- | --- |
| female C57Bl/6NCrl | 1 | NA | NA |
| male AMB-YLF-GGG | 1 | 1 | NA |
| male C57Bl/6NCrl | 1 | 1 | 1 |

### 4.13.50 Ulna

|  | female AMB-YLF-GGG | female C57Bl/6NCrl | male AMB-YLF-GGG |
| --- | --- | --- | --- |
| female C57Bl/6NCrl | 1 | NA | NA |
| male AMB-YLF-GGG | 1 | 1 | NA |
| male C57Bl/6NCrl | 1 | 1 | 1 |

### Normal C57Bl/6N Male

#### Dorso Ventral

#### Dorso Ventral

#### Lateral Orientation

#### Forepaw

#### Skull Lateral Orientation

#### Skull Dorso Ventral Orientation

#### Hind Leg and Hip

#### Hind Leg and Hip

### Normal C57Bl/6N Female

#### Dorso Ventral

#### Dorso Ventral

#### Lateral Orientation

#### Forepaw

#### Skull Lateral Orientation

#### Skull Dorso Ventral Orientation

#### Hind Leg and Hip

#### Hind Leg and Hip

### E18-00633

#### Dorso Ventral

#### Dorso Ventral

#### Lateral Orientation

#### Forepaw

#### Skull Lateral Orientation

#### Skull Dorso Ventral Orientation

#### Hind Leg and Hip

#### Hind Leg and Hip

#### uCT image: detail 1

### E18-00634

#### Dorso Ventral

#### Dorso Ventral

#### Lateral Orientation

#### Forepaw

#### Skull Lateral Orientation

#### Skull Dorso Ventral Orientation

#### Hind Leg and Hip

#### Hind Leg and Hip

#### uCT image: detail 1

### E18-00635

#### Dorso Ventral

#### Dorso Ventral

#### Lateral Orientation

#### Forepaw

#### Skull Lateral Orientation

#### Skull Dorso Ventral Orientation

#### Hind Leg and Hip

#### Hind Leg and Hip

#### uCT image: detail 1

### E18-00637

#### Dorso Ventral

#### Dorso Ventral

#### Lateral Orientation

#### Forepaw

#### Skull Lateral Orientation

#### Skull Dorso Ventral Orientation

#### Hind Leg and Hip

#### Hind Leg and Hip

#### uCT image: detail 2

#### uCT image: detail 1

### E18-00638

#### Dorso Ventral

#### Dorso Ventral

#### Lateral Orientation

#### Forepaw

#### Skull Lateral Orientation

#### Skull Dorso Ventral Orientation

#### Hind Leg and Hip

#### Hind Leg and Hip

#### uCT image: detail 1

### E18-00640

#### Dorso Ventral

#### Dorso Ventral

#### Lateral Orientation

#### Forepaw

#### Skull Lateral Orientation

#### Skull Dorso Ventral Orientation

#### Hind Leg and Hip

#### Hind Leg and Hip

#### uCT image: detail 1

### E18-00642

#### Dorso Ventral

#### Dorso Ventral

#### Lateral Orientation

#### Forepaw

#### Skull Lateral Orientation

#### Skull Dorso Ventral Orientation

#### Hind Leg and Hip

#### Hind Leg and Hip

### E18-00643

#### Dorso Ventral

#### Dorso Ventral

#### Lateral Orientation

#### Forepaw

#### Skull Lateral Orientation

#### Skull Dorso Ventral Orientation

#### Hind Leg and Hip

#### Hind Leg and Hip

#### uCT image: detail 1

### E18-00644

#### Dorso Ventral

#### Dorso Ventral

#### Lateral Orientation

#### Forepaw

#### Skull Lateral Orientation

#### Skull Dorso Ventral Orientation

#### Hind Leg and Hip

#### Hind Leg and Hip

#### uCT image: detail 1

### E18-00645

#### Dorso Ventral

#### Dorso Ventral

#### Lateral Orientation

#### Forepaw

#### Skull Lateral Orientation

#### Skull Dorso Ventral Orientation

#### Hind Leg and Hip

#### Hind Leg and Hip

#### uCT image: detail 1

### E18-00646

#### Dorso Ventral

#### Dorso Ventral

#### Lateral Orientation

#### Forepaw

#### Skull Lateral Orientation

#### Skull Dorso Ventral Orientation

#### Hind Leg and Hip

#### Hind Leg and Hip

### E18-00647

#### Dorso Ventral

#### Dorso Ventral

#### Lateral Orientation

#### Forepaw

#### Skull Lateral Orientation

#### Skull Dorso Ventral Orientation

#### Hind Leg and Hip

#### Hind Leg and Hip

### E18-00649

#### Dorso Ventral

#### Dorso Ventral

#### Lateral Orientation

#### Forepaw

#### Skull Lateral Orientation

#### Skull Dorso Ventral Orientation

#### Hind Leg and Hip

#### Hind Leg and Hip

#### uCT image: detail 1

### E18-00651

#### Dorso Ventral

#### Dorso Ventral

#### Lateral Orientation

#### Forepaw

#### Skull Lateral Orientation

#### Skull Dorso Ventral Orientation

#### Hind Leg and Hip

#### Hind Leg and Hip

## 4.14 Hematology

**Number of animals**

| STRAIN | GENDER | count |
| --- | --- | --- |
| AMB-YLF-GGG | female | 8 |
| AMB-YLF-GGG | male | 7 |
| C57Bl/6NCrl | female | 923 |
| C57Bl/6NCrl | male | 879 |

### 4.14.1 Eosinophil cell count

|  | test | Difference | p\_value | Significance |
| --- | --- | --- | --- | --- |
| c2 | CTRl vs KO | -0.0088 | 0.9181 | FALSE |
| c3 | Female vs Male | -0.0145 | 0.7134 | FALSE |
| c4 | Female CTRL vs Female KO | -0.0121 | 0.9209 | FALSE |
| c5 | Male CTRL vs Male KO | -0.0055 | 0.9922 | FALSE |
| c6 | Female KO vs Male KO | -0.0112 | 0.0000 | TRUE |

### 4.14.2 Eosinophil differential count

|  | test | Difference | p\_value | Significance |
| --- | --- | --- | --- | --- |
| c2 | CTRl vs KO | -0.1831 | 0.9006 | FALSE |
| c3 | Female vs Male | -0.1120 | 0.9742 | FALSE |
| c4 | Female CTRL vs Female KO | -0.3694 | 0.7490 | FALSE |
| c5 | Male CTRL vs Male KO | 0.0031 | 1.0000 | FALSE |
| c6 | Female KO vs Male KO | 0.0742 | 0.1699 | FALSE |

### 4.14.3 Hematocrit

|  | test | Difference | p\_value | Significance |
| --- | --- | --- | --- | --- |
| c2 | CTRl vs KO | 1.8214 | 0.2952 | FALSE |
| c3 | Female vs Male | -0.2669 | 0.9937 | FALSE |
| c4 | Female CTRL vs Female KO | 1.5990 | 0.6815 | FALSE |
| c5 | Male CTRL vs Male KO | 2.0439 | 0.5080 | FALSE |
| c6 | Female KO vs Male KO | -0.0444 | 0.9844 | FALSE |

### 4.14.4 Hemoglobin

|  | test | Difference | p\_value | Significance |
| --- | --- | --- | --- | --- |
| c2 | CTRl vs KO | 0.6006 | 0.0897 | FALSE |
| c3 | Female vs Male | 0.0238 | 0.9997 | FALSE |
| c4 | Female CTRL vs Female KO | 0.5144 | 0.4643 | FALSE |
| c5 | Male CTRL vs Male KO | 0.6869 | 0.2366 | FALSE |
| c6 | Female KO vs Male KO | 0.1100 | 0.0066 | TRUE |

### 4.14.5 Lymphocyte cell count

|  | test | Difference | p\_value | Significance |
| --- | --- | --- | --- | --- |
| c2 | CTRl vs KO | -0.3062 | 0.9720 | FALSE |
| c3 | Female vs Male | -1.4995 | 0.1476 | FALSE |
| c4 | Female CTRL vs Female KO | -0.3163 | 0.9884 | FALSE |
| c5 | Male CTRL vs Male KO | -0.2961 | 0.9908 | FALSE |
| c6 | Female KO vs Male KO | -1.4894 | 0.0000 | TRUE |

### 4.14.6 Lymphocyte differential count

|  | test | Difference | p\_value | Significance |
| --- | --- | --- | --- | --- |
| c2 | CTRl vs KO | 0.5035 | 0.9939 | FALSE |
| c3 | Female vs Male | 3.3775 | 0.3116 | FALSE |
| c4 | Female CTRL vs Female KO | 1.1511 | 0.9739 | FALSE |
| c5 | Male CTRL vs Male KO | -0.1440 | 1.0000 | FALSE |
| c6 | Female KO vs Male KO | 2.7299 | 0.0000 | TRUE |

### 4.14.7 Mean cell hemoglobin concentration

|  | test | Difference | p\_value | Significance |
| --- | --- | --- | --- | --- |
| c2 | CTRl vs KO | 0.0999 | 0.9914 | FALSE |
| c3 | Female vs Male | 0.2361 | 0.9007 | FALSE |
| c4 | Female CTRL vs Female KO | 0.0549 | 0.9995 | FALSE |
| c5 | Male CTRL vs Male KO | 0.1449 | 0.9909 | FALSE |
| c6 | Female KO vs Male KO | 0.2811 | 0.0000 | TRUE |

### 4.14.8 Mean cell volume

|  | test | Difference | p\_value | Significance |
| --- | --- | --- | --- | --- |
| c2 | CTRl vs KO | -0.7351 | 0.5682 | FALSE |
| c3 | Female vs Male | 0.3221 | 0.9405 | FALSE |
| c4 | Female CTRL vs Female KO | -1.3679 | 0.3138 | FALSE |
| c5 | Male CTRL vs Male KO | -0.1024 | 0.9993 | FALSE |
| c6 | Female KO vs Male KO | 0.9548 | 0.0000 | TRUE |

### 4.14.9 Mean corpuscular hemoglobin

|  | test | Difference | p\_value | Significance |
| --- | --- | --- | --- | --- |
| c2 | CTRl vs KO | -0.1780 | 0.6666 | FALSE |
| c3 | Female vs Male | 0.2391 | 0.4210 | FALSE |
| c4 | Female CTRL vs Female KO | -0.3795 | 0.3069 | FALSE |
| c5 | Male CTRL vs Male KO | 0.0236 | 0.9996 | FALSE |
| c6 | Female KO vs Male KO | 0.4406 | 0.0000 | TRUE |

### 4.14.10 Mean platelet volume

|  | test | Difference | p\_value | Significance |
| --- | --- | --- | --- | --- |
| c2 | CTRl vs KO | -0.1665 | 0.1439 | FALSE |
| c3 | Female vs Male | -0.0032 | 1.0000 | FALSE |
| c4 | Female CTRL vs Female KO | -0.2669 | 0.0717 | FALSE |
| c5 | Male CTRL vs Male KO | -0.0661 | 0.9309 | FALSE |
| c6 | Female KO vs Male KO | 0.0972 | 0.0000 | TRUE |

### 4.14.11 Monocyte cell count

|  | test | Difference | p\_value | Significance |
| --- | --- | --- | --- | --- |
| c2 | CTRl vs KO | -0.0212 | 0.9922 | FALSE |
| c3 | Female vs Male | -0.1817 | 0.0811 | FALSE |
| c4 | Female CTRL vs Female KO | -0.0336 | 0.9886 | FALSE |
| c5 | Male CTRL vs Male KO | -0.0088 | 0.9998 | FALSE |
| c6 | Female KO vs Male KO | -0.1693 | 0.0000 | TRUE |

### 4.14.12 Monocyte differential count

|  | test | Difference | p\_value | Significance |
| --- | --- | --- | --- | --- |
| c2 | CTRl vs KO | 0.2817 | 0.9819 | FALSE |
| c3 | Female vs Male | -1.1602 | 0.4133 | FALSE |
| c4 | Female CTRL vs Female KO | 0.1508 | 0.9989 | FALSE |
| c5 | Male CTRL vs Male KO | 0.4126 | 0.9807 | FALSE |
| c6 | Female KO vs Male KO | -1.0293 | 0.0000 | TRUE |

### 4.14.13 Neutrophil cell count

|  | test | Difference | p\_value | Significance |
| --- | --- | --- | --- | --- |
| c2 | CTRl vs KO | -0.0789 | 0.9532 | FALSE |
| c3 | Female vs Male | -0.3497 | 0.1003 | FALSE |
| c4 | Female CTRL vs Female KO | -0.0928 | 0.9711 | FALSE |
| c5 | Male CTRL vs Male KO | -0.0651 | 0.9903 | FALSE |
| c6 | Female KO vs Male KO | -0.3359 | 0.0000 | TRUE |

### 4.14.14 Neutrophil differential count

|  | test | Difference | p\_value | Significance |
| --- | --- | --- | --- | --- |
| c2 | CTRl vs KO | -0.6527 | 0.9800 | FALSE |
| c3 | Female vs Male | -2.0374 | 0.6216 | FALSE |
| c4 | Female CTRL vs Female KO | -1.0511 | 0.9696 | FALSE |
| c5 | Male CTRL vs Male KO | -0.2544 | 0.9996 | FALSE |
| c6 | Female KO vs Male KO | -1.6390 | 0.0000 | TRUE |

### 4.14.15 Platelet count

|  | test | Difference | p\_value | Significance |
| --- | --- | --- | --- | --- |
| c2 | CTRl vs KO | -64.2022 | 0.6764 | FALSE |
| c3 | Female vs Male | -123.5168 | 0.1410 | FALSE |
| c4 | Female CTRL vs Female KO | -17.8461 | 0.9960 | FALSE |
| c5 | Male CTRL vs Male KO | -110.5583 | 0.5344 | FALSE |
| c6 | Female KO vs Male KO | -169.8729 | 0.0000 | TRUE |

### 4.14.16 Platelet crit

ERROR : undefined columns selected

ERROR : object ‘Platelet crit’ not found

### 4.14.17 Platelet distribution width

ERROR : undefined columns selected

ERROR : object ‘Platelet distribution width’ not found

### 4.14.18 Red blood cell count

|  | test | Difference | p\_value | Significance |
| --- | --- | --- | --- | --- |
| c2 | CTRl vs KO | 0.4843 | 0.0615 | FALSE |
| c3 | Female vs Male | -0.1106 | 0.9370 | FALSE |
| c4 | Female CTRL vs Female KO | 0.5408 | 0.1819 | FALSE |
| c5 | Male CTRL vs Male KO | 0.4278 | 0.4050 | FALSE |
| c6 | Female KO vs Male KO | -0.1672 | 0.0000 | TRUE |

### 4.14.19 Red blood cell distribution width

|  | test | Difference | p\_value | Significance |
| --- | --- | --- | --- | --- |
| c2 | CTRl vs KO | -0.6446 | 0.0493 | TRUE |
| c3 | Female vs Male | -0.4437 | 0.2733 | FALSE |
| c4 | Female CTRL vs Female KO | -0.6849 | 0.1927 | FALSE |
| c5 | Male CTRL vs Male KO | -0.6043 | 0.3214 | FALSE |
| c6 | Female KO vs Male KO | -0.4034 | 0.0000 | TRUE |

### 4.14.20 Red blood cell distribution width %

ERROR : undefined columns selected

ERROR : object ‘Red blood cell distribution width %’ not found

### 4.14.21 White blood cell count

|  | test | Difference | p\_value | Significance |
| --- | --- | --- | --- | --- |
| c2 | CTRl vs KO | -0.4143 | 0.9640 | FALSE |
| c3 | Female vs Male | -2.0694 | 0.0867 | FALSE |
| c4 | Female CTRL vs Female KO | -0.4300 | 0.9848 | FALSE |
| c5 | Male CTRL vs Male KO | -0.3985 | 0.9883 | FALSE |
| c6 | Female KO vs Male KO | -2.0537 | 0.0000 | TRUE |

## 4.15 Biochemistry

**Number of animals**

| STRAIN | GENDER | count |
| --- | --- | --- |
| AMB-YLF-GGG | female | 8 |
| AMB-YLF-GGG | male | 7 |
| C57Bl/6NCrl | female | 923 |
| C57Bl/6NCrl | male | 876 |

### 4.15.1 Alanine aminotransferase

|  | test | Difference | p\_value | Significance |
| --- | --- | --- | --- | --- |
| c2 | CTRl vs KO | -2.4414 | 0.9089 | FALSE |
| c3 | Female vs Male | -3.7242 | 0.7341 | FALSE |
| c4 | Female CTRL vs Female KO | -0.8065 | 0.9985 | FALSE |
| c5 | Male CTRL vs Male KO | -4.0764 | 0.8654 | FALSE |
| c6 | Female KO vs Male KO | -5.3591 | 0.0000 | TRUE |

### 4.15.2 Albumin

|  | test | Difference | p\_value | Significance |
| --- | --- | --- | --- | --- |
| c2 | CTRl vs KO | 2.3549 | 0.0783 | FALSE |
| c3 | Female vs Male | 1.2365 | 0.5721 | FALSE |
| c4 | Female CTRL vs Female KO | 2.3005 | 0.3331 | FALSE |
| c5 | Male CTRL vs Male KO | 2.4092 | 0.2985 | FALSE |
| c6 | Female KO vs Male KO | 1.2908 | 0.0000 | TRUE |

### 4.15.3 Alkaline phosphatase

|  | test | Difference | p\_value | Significance |
| --- | --- | --- | --- | --- |
| c2 | CTRl vs KO | 11.6781 | 0.4553 | FALSE |
| c3 | Female vs Male | 35.5269 | 0.0001 | TRUE |
| c4 | Female CTRL vs Female KO | 8.0798 | 0.8849 | FALSE |
| c5 | Male CTRL vs Male KO | 15.2764 | 0.5255 | FALSE |
| c6 | Female KO vs Male KO | 39.1252 | 0.0000 | TRUE |

### 4.15.4 Aspartate aminotransferase

|  | test | Difference | p\_value | Significance |
| --- | --- | --- | --- | --- |
| c2 | CTRl vs KO | -4.2852 | 0.9153 | FALSE |
| c3 | Female vs Male | 4.6522 | 0.8920 | FALSE |
| c4 | Female CTRL vs Female KO | 0.3841 | 1.0000 | FALSE |
| c5 | Male CTRL vs Male KO | -8.9545 | 0.7782 | FALSE |
| c6 | Female KO vs Male KO | -0.0171 | 1.0000 | FALSE |

### 4.15.5 Calcium

|  | test | Difference | p\_value | Significance |
| --- | --- | --- | --- | --- |
| c2 | CTRl vs KO | -0.0690 | 0.9909 | FALSE |
| c3 | Female vs Male | -0.0915 | 0.9789 | FALSE |
| c4 | Female CTRL vs Female KO | -0.0989 | 0.9904 | FALSE |
| c5 | Male CTRL vs Male KO | -0.0390 | 0.9994 | FALSE |
| c6 | Female KO vs Male KO | -0.0615 | 0.0001 | TRUE |

### 4.15.6 Chloride

|  | test | Difference | p\_value | Significance |
| --- | --- | --- | --- | --- |
| c2 | CTRl vs KO | -0.2049 | 0.9990 | FALSE |
| c3 | Female vs Male | 1.9482 | 0.5407 | FALSE |
| c4 | Female CTRL vs Female KO | -0.6620 | 0.9884 | FALSE |
| c5 | Male CTRL vs Male KO | 0.2522 | 0.9993 | FALSE |
| c6 | Female KO vs Male KO | 2.4053 | 0.0000 | TRUE |

### 4.15.7 Creatinine

|  | test | Difference | p\_value | Significance |
| --- | --- | --- | --- | --- |
| c2 | CTRl vs KO | -0.0089 | 0.6655 | FALSE |
| c3 | Female vs Male | 0.0068 | 0.8110 | FALSE |
| c4 | Female CTRL vs Female KO | -0.0086 | 0.8608 | FALSE |
| c5 | Male CTRL vs Male KO | -0.0092 | 0.8380 | FALSE |
| c6 | Female KO vs Male KO | 0.0066 | 0.0000 | TRUE |

### 4.15.8 Glucose

|  | test | Difference | p\_value | Significance |
| --- | --- | --- | --- | --- |
| c2 | CTRl vs KO | -38.3454 | 0.0372 | TRUE |
| c3 | Female vs Male | 0.8782 | 0.9999 | FALSE |
| c4 | Female CTRL vs Female KO | -36.1380 | 0.2595 | FALSE |
| c5 | Male CTRL vs Male KO | -40.5528 | 0.1825 | FALSE |
| c6 | Female KO vs Male KO | -1.3292 | 0.7960 | FALSE |

### 4.15.9 HDL-cholesterol

|  | test | Difference | p\_value | Significance |
| --- | --- | --- | --- | --- |
| c2 | CTRl vs KO | 1.2694 | 0.9846 | FALSE |
| c3 | Female vs Male | -9.6121 | 0.0397 | TRUE |
| c4 | Female CTRL vs Female KO | 1.7461 | 0.9850 | FALSE |
| c5 | Male CTRL vs Male KO | 0.7928 | 0.9987 | FALSE |
| c6 | Female KO vs Male KO | -10.0887 | 0.0000 | TRUE |

### 4.15.10 Iron

|  | test | Difference | p\_value | Significance |
| --- | --- | --- | --- | --- |
| c2 | CTRl vs KO | 0.0048 | 0.9238 | FALSE |
| c3 | Female vs Male | 0.0140 | 0.2638 | FALSE |
| c4 | Female CTRL vs Female KO | 0.0007 | 0.9999 | FALSE |
| c5 | Male CTRL vs Male KO | 0.0089 | 0.8459 | FALSE |
| c6 | Female KO vs Male KO | 0.0182 | 0.0000 | TRUE |

### 4.15.11 Phosphate

|  | test | Difference | p\_value | Significance |
| --- | --- | --- | --- | --- |
| c2 | CTRl vs KO | -0.1555 | 0.9802 | FALSE |
| c3 | Female vs Male | -0.1325 | 0.9872 | FALSE |
| c4 | Female CTRL vs Female KO | -0.7165 | 0.5809 | FALSE |
| c5 | Male CTRL vs Male KO | 0.4054 | 0.8930 | FALSE |
| c6 | Female KO vs Male KO | 0.4284 | 0.0000 | TRUE |

### 4.15.12 Potassium

|  | test | Difference | p\_value | Significance |
| --- | --- | --- | --- | --- |
| c2 | CTRl vs KO | 0.3389 | 0.6739 | FALSE |
| c3 | Female vs Male | -0.5509 | 0.2592 | FALSE |
| c4 | Female CTRL vs Female KO | 0.5114 | 0.6198 | FALSE |
| c5 | Male CTRL vs Male KO | 0.1664 | 0.9795 | FALSE |
| c6 | Female KO vs Male KO | -0.7233 | 0.0000 | TRUE |

### 4.15.13 Sodium

|  | test | Difference | p\_value | Significance |
| --- | --- | --- | --- | --- |
| c2 | CTRl vs KO | 0.6258 | 0.9820 | FALSE |
| c3 | Female vs Male | -0.9656 | 0.9372 | FALSE |
| c4 | Female CTRL vs Female KO | 0.1093 | 1.0000 | FALSE |
| c5 | Male CTRL vs Male KO | 1.1424 | 0.9624 | FALSE |
| c6 | Female KO vs Male KO | -0.4491 | 0.0000 | TRUE |

### 4.15.14 Total bilirubin

|  | test | Difference | p\_value | Significance |
| --- | --- | --- | --- | --- |
| c2 | CTRl vs KO | -0.0283 | 0.4594 | FALSE |
| c3 | Female vs Male | -0.0204 | 0.7085 | FALSE |
| c4 | Female CTRL vs Female KO | -0.0368 | 0.5154 | FALSE |
| c5 | Male CTRL vs Male KO | -0.0198 | 0.8888 | FALSE |
| c6 | Female KO vs Male KO | -0.0119 | 0.0000 | TRUE |

### 4.15.15 Total cholesterol

|  | test | Difference | p\_value | Significance |
| --- | --- | --- | --- | --- |
| c2 | CTRl vs KO | -5.8858 | 0.6225 | FALSE |
| c3 | Female vs Male | -11.8536 | 0.0740 | FALSE |
| c4 | Female CTRL vs Female KO | -6.6426 | 0.7588 | FALSE |
| c5 | Male CTRL vs Male KO | -5.1290 | 0.8811 | FALSE |
| c6 | Female KO vs Male KO | -11.0968 | 0.0000 | TRUE |

### 4.15.16 Total protein

|  | test | Difference | p\_value | Significance |
| --- | --- | --- | --- | --- |
| c2 | CTRl vs KO | -0.6184 | 0.9676 | FALSE |
| c3 | Female vs Male | -0.1189 | 0.9997 | FALSE |
| c4 | Female CTRL vs Female KO | -0.5536 | 0.9912 | FALSE |
| c5 | Male CTRL vs Male KO | -0.6832 | 0.9840 | FALSE |
| c6 | Female KO vs Male KO | -0.1836 | 0.1864 | FALSE |

### 4.15.17 Triglycerides

|  | test | Difference | p\_value | Significance |
| --- | --- | --- | --- | --- |
| c2 | CTRl vs KO | -4.8607 | 0.9074 | FALSE |
| c3 | Female vs Male | -25.2156 | 0.0036 | TRUE |
| c4 | Female CTRL vs Female KO | -11.5020 | 0.6630 | FALSE |
| c5 | Male CTRL vs Male KO | 1.7806 | 0.9982 | FALSE |
| c6 | Female KO vs Male KO | -18.5743 | 0.0000 | TRUE |

### 4.15.18 Urea (Blood Urea Nitrogen - BUN)

|  | test | Difference | p\_value | Significance |
| --- | --- | --- | --- | --- |
| c2 | CTRl vs KO | 2.8916 | 0.3474 | FALSE |
| c3 | Female vs Male | -1.5414 | 0.8063 | FALSE |
| c4 | Female CTRL vs Female KO | 3.5967 | 0.4507 | FALSE |
| c5 | Male CTRL vs Male KO | 2.1865 | 0.8108 | FALSE |
| c6 | Female KO vs Male KO | -2.2465 | 0.0000 | TRUE |

## 4.16 Organ weight

**Number of animals**

| STRAIN | GENDER | count |
| --- | --- | --- |
| AMB-YLF-GGG | female | 8 |
| AMB-YLF-GGG | male | 7 |
| C57Bl/6NCrl | female | 918 |
| C57Bl/6NCrl | male | 875 |

### 4.16.1 Heart weight

|  | test | Difference | p\_value | Significance |
| --- | --- | --- | --- | --- |
| c2 | CTRl vs KO | -46.1782 | 0.0049 | TRUE |
| c3 | Female vs Male | -22.1618 | 0.3592 | FALSE |
| c4 | Female CTRL vs Female KO | -36.2486 | 0.2291 | FALSE |
| c5 | Male CTRL vs Male KO | -56.1078 | 0.0225 | TRUE |
| c6 | Female KO vs Male KO | -32.0914 | 0.0000 | TRUE |

### 4.16.2 Heart weight normalised against body weight

|  | test | Difference | p\_value | Significance |
| --- | --- | --- | --- | --- |
| c2 | CTRl vs KO | -0.0010 | 0.1499 | FALSE |
| c3 | Female vs Male | 0.0001 | 0.9954 | FALSE |
| c4 | Female CTRL vs Female KO | -0.0008 | 0.6701 | FALSE |
| c5 | Male CTRL vs Male KO | -0.0013 | 0.2389 | FALSE |
| c6 | Female KO vs Male KO | -0.0002 | 0.0117 | TRUE |

### 4.16.3 Tibia length

|  | test | Difference | p\_value | Significance |
| --- | --- | --- | --- | --- |
| c2 | CTRl vs KO | -1.1508 | 0.0363 | TRUE |
| c3 | Female vs Male | 0.2839 | 0.9033 | FALSE |
| c4 | Female CTRL vs Female KO | -0.6870 | 0.6418 | FALSE |
| c5 | Male CTRL vs Male KO | -1.6146 | 0.0411 | TRUE |
| c6 | Female KO vs Male KO | -0.1799 | 0.0057 | TRUE |

### 4.16.4 Tibia length normalised against body weight

|  | test | Difference | p\_value | Significance |
| --- | --- | --- | --- | --- |
| c2 | CTRl vs KO | 0.0828 | 0.0079 | TRUE |
| c3 | Female vs Male | 0.1308 | 0.0000 | TRUE |
| c4 | Female CTRL vs Female KO | 0.1573 | 0.0001 | TRUE |
| c5 | Male CTRL vs Male KO | 0.0083 | 0.9956 | FALSE |
| c6 | Female KO vs Male KO | 0.0563 | 0.0000 | TRUE |

### 4.16.5 Body length

|  | test | Difference | p\_value | Significance |
| --- | --- | --- | --- | --- |
| c2 | CTRl vs KO | -4.2601 | 0.0926 | FALSE |
| c3 | Female vs Male | -1.5652 | 0.8205 | FALSE |
| c4 | Female CTRL vs Female KO | -3.7842 | 0.4432 | FALSE |
| c5 | Male CTRL vs Male KO | -4.7361 | 0.2618 | FALSE |
| c6 | Female KO vs Male KO | -2.0412 | 0.0000 | TRUE |

### 4.16.6 Body weight

|  | test | Difference | p\_value | Significance |
| --- | --- | --- | --- | --- |
| c2 | CTRl vs KO | -4.1031 | 0.0000 | TRUE |
| c3 | Female vs Male | -4.4870 | 0.0000 | TRUE |
| c4 | Female CTRL vs Female KO | -3.8857 | 0.0011 | TRUE |
| c5 | Male CTRL vs Male KO | -4.3205 | 0.0003 | TRUE |
| c6 | Female KO vs Male KO | -4.7044 | 0.0000 | TRUE |

### 4.16.7 Large intestine length

|  | test | Difference | p\_value | Significance |
| --- | --- | --- | --- | --- |
| c2 | CTRl vs KO | -4.7490 | 0.8771 | FALSE |
| c3 | Female vs Male | -7.4751 | 0.6418 | FALSE |
| c4 | Female CTRL vs Female KO | -6.0239 | 0.9043 | FALSE |
| c5 | Male CTRL vs Male KO | -3.4741 | 0.9808 | FALSE |
| c6 | Female KO vs Male KO | -6.2002 | 0.0000 | TRUE |

### 4.16.8 Left kidney weight

|  | test | Difference | p\_value | Significance |
| --- | --- | --- | --- | --- |
| c2 | CTRl vs KO | -0.0417 | 0.0011 | TRUE |
| c3 | Female vs Male | -0.0404 | 0.0018 | TRUE |
| c4 | Female CTRL vs Female KO | -0.0317 | 0.1658 | FALSE |
| c5 | Male CTRL vs Male KO | -0.0518 | 0.0065 | TRUE |
| c6 | Female KO vs Male KO | -0.0504 | 0.0000 | TRUE |

### 4.16.9 Liver weight

|  | test | Difference | p\_value | Significance |
| --- | --- | --- | --- | --- |
| c2 | CTRl vs KO | -0.2209 | 0.0037 | TRUE |
| c3 | Female vs Male | -0.2991 | 0.0000 | TRUE |
| c4 | Female CTRL vs Female KO | -0.2031 | 0.1024 | FALSE |
| c5 | Male CTRL vs Male KO | -0.2388 | 0.0475 | TRUE |
| c6 | Female KO vs Male KO | -0.3169 | 0.0000 | TRUE |

### 4.16.10 Lung weight

|  | test | Difference | p\_value | Significance |
| --- | --- | --- | --- | --- |
| c2 | CTRl vs KO | -0.0256 | 0.3406 | FALSE |
| c3 | Female vs Male | -0.0115 | 0.8746 | FALSE |
| c4 | Female CTRL vs Female KO | -0.0288 | 0.5362 | FALSE |
| c5 | Male CTRL vs Male KO | -0.0224 | 0.7297 | FALSE |
| c6 | Female KO vs Male KO | -0.0083 | 0.0000 | TRUE |

### 4.16.11 Right kidney weight

|  | test | Difference | p\_value | Significance |
| --- | --- | --- | --- | --- |
| c2 | CTRl vs KO | -0.0427 | 0.0131 | TRUE |
| c3 | Female vs Male | -0.0350 | 0.0596 | FALSE |
| c4 | Female CTRL vs Female KO | -0.0289 | 0.4338 | FALSE |
| c5 | Male CTRL vs Male KO | -0.0564 | 0.0268 | TRUE |
| c6 | Female KO vs Male KO | -0.0487 | 0.0000 | TRUE |

### 4.16.12 Small intestine length

|  | test | Difference | p\_value | Significance |
| --- | --- | --- | --- | --- |
| c2 | CTRl vs KO | -14.0128 | 0.5344 | FALSE |
| c3 | Female vs Male | -10.3495 | 0.7508 | FALSE |
| c4 | Female CTRL vs Female KO | -15.9847 | 0.6869 | FALSE |
| c5 | Male CTRL vs Male KO | -12.0408 | 0.8501 | FALSE |
| c6 | Female KO vs Male KO | -8.3775 | 0.0000 | TRUE |

### 4.16.13 Spleen weight

|  | test | Difference | p\_value | Significance |
| --- | --- | --- | --- | --- |
| c2 | CTRl vs KO | -0.0201 | 0.0808 | FALSE |
| c3 | Female vs Male | 0.0100 | 0.6258 | FALSE |
| c4 | Female CTRL vs Female KO | -0.0188 | 0.3731 | FALSE |
| c5 | Male CTRL vs Male KO | -0.0214 | 0.2783 | FALSE |
| c6 | Female KO vs Male KO | 0.0087 | 0.0000 | TRUE |

### 4.16.14 Thorax size

|  | test | Difference | p\_value | Significance |
| --- | --- | --- | --- | --- |
| c2 | CTRl vs KO | -0.9554 | 0.5113 | FALSE |
| c3 | Female vs Male | -2.1149 | 0.0146 | TRUE |
| c4 | Female CTRL vs Female KO | -1.0012 | 0.7247 | FALSE |
| c5 | Male CTRL vs Male KO | -0.9095 | 0.7933 | FALSE |
| c6 | Female KO vs Male KO | -2.0691 | 0.0000 | TRUE |

### 4.16.15 Testes weight (sin.)

ERROR : Can’t subset columns that don’t exist. ✖ Location 2 doesn’t exist. ℹ There are only 1 column.

### 4.16.16 Testes weight (dx.)

ERROR : Can’t subset columns that don’t exist. ✖ Location 2 doesn’t exist. ℹ There are only 1 column.

## 4.17 Immunology

**Number of animals**

| STRAIN | GENDER | count |
| --- | --- | --- |
| AMB-YLF-GGG | female | 8 |
| AMB-YLF-GGG | male | 7 |
| C57Bl/6NCrl | female | 661 |
| C57Bl/6NCrl | male | 602 |

```
Error in svd(x, nu = 0, nv = k): a dimension is zero
```

### 4.17.1 B cells - % of live leukocytes (Panel B)

|  | test | Difference | p\_value | Significance |
| --- | --- | --- | --- | --- |
| c2 | CTRl vs KO | 5.5418 | 0.2447 | FALSE |
| c3 | Female vs Male | -3.5066 | 0.6328 | FALSE |
| c4 | Female CTRL vs Female KO | 4.1776 | 0.7415 | FALSE |
| c5 | Male CTRL vs Male KO | 6.9060 | 0.3609 | FALSE |
| c6 | Female KO vs Male KO | -2.1424 | 0.0000 | TRUE |

### 4.17.2 B1a cells - % of B cells (Panel B)

|  | test | Difference | p\_value | Significance |
| --- | --- | --- | --- | --- |
| c2 | CTRl vs KO | 0.8625 | 0.0986 | FALSE |
| c3 | Female vs Male | 0.2653 | 0.8886 | FALSE |
| c4 | Female CTRL vs Female KO | 0.8568 | 0.3560 | FALSE |
| c5 | Male CTRL vs Male KO | 0.8682 | 0.3544 | FALSE |
| c6 | Female KO vs Male KO | 0.2709 | 0.0000 | TRUE |

### 4.17.3 B1a cells - % of live(Panel B)

|  | test | Difference | p\_value | Significance |
| --- | --- | --- | --- | --- |
| c2 | CTRl vs KO | 0.5015 | 0.0290 | TRUE |
| c3 | Female vs Male | 0.0801 | 0.9687 | FALSE |
| c4 | Female CTRL vs Female KO | 0.4764 | 0.2312 | FALSE |
| c5 | Male CTRL vs Male KO | 0.5266 | 0.1636 | FALSE |
| c6 | Female KO vs Male KO | 0.1052 | 0.0000 | TRUE |

### 4.17.4 CD11b- NK cells - % of live leukocytes (Panel B)

|  | test | Difference | p\_value | Significance |
| --- | --- | --- | --- | --- |
| c2 | CTRl vs KO | -0.7094 | 0.0015 | TRUE |
| c3 | Female vs Male | -0.1375 | 0.8852 | FALSE |
| c4 | Female CTRL vs Female KO | -0.5332 | 0.1900 | FALSE |
| c5 | Male CTRL vs Male KO | -0.8857 | 0.0079 | TRUE |
| c6 | Female KO vs Male KO | -0.3137 | 0.0000 | TRUE |

### 4.17.5 CD11b- NK cells - % of NK cells (Panel B)

|  | test | Difference | p\_value | Significance |
| --- | --- | --- | --- | --- |
| c2 | CTRl vs KO | -14.7176 | 0.0084 | TRUE |
| c3 | Female vs Male | -4.3142 | 0.7742 | FALSE |
| c4 | Female CTRL vs Female KO | -13.0367 | 0.1767 | FALSE |
| c5 | Male CTRL vs Male KO | -16.3984 | 0.0600 | FALSE |
| c6 | Female KO vs Male KO | -5.9951 | 0.0000 | TRUE |

### 4.17.6 CD11b-high cDC - % of conventional DC (Panel B)

|  | test | Difference | p\_value | Significance |
| --- | --- | --- | --- | --- |
| c2 | CTRl vs KO | 5.7996 | 0.4690 | FALSE |
| c3 | Female vs Male | -3.8512 | 0.7671 | FALSE |
| c4 | Female CTRL vs Female KO | 4.3799 | 0.8614 | FALSE |
| c5 | Male CTRL vs Male KO | 7.2192 | 0.5819 | FALSE |
| c6 | Female KO vs Male KO | -2.4315 | 0.0000 | TRUE |

### 4.17.7 CD11b-high cDC - % of live leukocytes (Panel B)

|  | test | Difference | p\_value | Significance |
| --- | --- | --- | --- | --- |
| c2 | CTRl vs KO | 0.3291 | 0.1349 | FALSE |
| c3 | Female vs Male | -0.2295 | 0.4239 | FALSE |
| c4 | Female CTRL vs Female KO | 0.3932 | 0.2463 | FALSE |
| c5 | Male CTRL vs Male KO | 0.2650 | 0.6081 | FALSE |
| c6 | Female KO vs Male KO | -0.2937 | 0.0000 | TRUE |

### 4.17.8 CD11b-low cDC - % of conventional DC (Panel B)

|  | test | Difference | p\_value | Significance |
| --- | --- | --- | --- | --- |
| c2 | CTRl vs KO | -3.8209 | 0.7532 | FALSE |
| c3 | Female vs Male | 3.8298 | 0.7503 | FALSE |
| c4 | Female CTRL vs Female KO | -2.4340 | 0.9691 | FALSE |
| c5 | Male CTRL vs Male KO | -5.2078 | 0.7766 | FALSE |
| c6 | Female KO vs Male KO | 2.4429 | 0.0000 | TRUE |

### 4.17.9 CD11b-low cDC - % of of live leukocytes (Panel B)

|  | test | Difference | p\_value | Significance |
| --- | --- | --- | --- | --- |
| c2 | CTRl vs KO | -0.0145 | 0.9998 | FALSE |
| c3 | Female vs Male | 0.0285 | 0.9987 | FALSE |
| c4 | Female CTRL vs Female KO | 0.0907 | 0.9850 | FALSE |
| c5 | Male CTRL vs Male KO | -0.1197 | 0.9680 | FALSE |
| c6 | Female KO vs Male KO | -0.0767 | 0.0000 | TRUE |

### 4.17.10 CD11b+ NK cells - % of live leukocytes (Panel B)

|  | test | Difference | p\_value | Significance |
| --- | --- | --- | --- | --- |
| c2 | CTRl vs KO | 0.1913 | 0.9428 | FALSE |
| c3 | Female vs Male | 0.3699 | 0.6962 | FALSE |
| c4 | Female CTRL vs Female KO | 0.3931 | 0.8395 | FALSE |
| c5 | Male CTRL vs Male KO | -0.0105 | 1.0000 | FALSE |
| c6 | Female KO vs Male KO | 0.1681 | 0.0001 | TRUE |

### 4.17.11 CD11b+ NK cells - % of NK cells (Panel B)

|  | test | Difference | p\_value | Significance |
| --- | --- | --- | --- | --- |
| c2 | CTRl vs KO | 17.0126 | 0.0025 | TRUE |
| c3 | Female vs Male | 4.4690 | 0.7720 | FALSE |
| c4 | Female CTRL vs Female KO | 15.1770 | 0.1020 | FALSE |
| c5 | Male CTRL vs Male KO | 18.8483 | 0.0281 | TRUE |
| c6 | Female KO vs Male KO | 6.3046 | 0.0000 | TRUE |

### 4.17.12 CD161+ B cells - % of B cells (Panel B)

|  | test | Difference | p\_value | Significance |
| --- | --- | --- | --- | --- |
| c2 | CTRl vs KO | -0.1183 | 0.8607 | FALSE |
| c3 | Female vs Male | -0.0447 | 0.9907 | FALSE |
| c4 | Female CTRL vs Female KO | -0.1828 | 0.8241 | FALSE |
| c5 | Male CTRL vs Male KO | -0.0539 | 0.9943 | FALSE |
| c6 | Female KO vs Male KO | 0.0198 | 0.0623 | FALSE |

### 4.17.13 CD161+ B cells - % of live leukocytes (Panel B)

|  | test | Difference | p\_value | Significance |
| --- | --- | --- | --- | --- |
| c2 | CTRl vs KO | -0.0395 | 0.9339 | FALSE |
| c3 | Female vs Male | -0.0318 | 0.9637 | FALSE |
| c4 | Female CTRL vs Female KO | -0.0739 | 0.8598 | FALSE |
| c5 | Male CTRL vs Male KO | -0.0052 | 0.9999 | FALSE |
| c6 | Female KO vs Male KO | 0.0026 | 0.8890 | FALSE |

### 4.17.14 CD4 T cells - % of live leukocytes (Panel A)

|  | test | Difference | p\_value | Significance |
| --- | --- | --- | --- | --- |
| c2 | CTRl vs KO | 2.2122 | 0.2449 | FALSE |
| c3 | Female vs Male | 1.0642 | 0.8013 | FALSE |
| c4 | Female CTRL vs Female KO | 1.8814 | 0.6549 | FALSE |
| c5 | Male CTRL vs Male KO | 2.5431 | 0.4441 | FALSE |
| c6 | Female KO vs Male KO | 1.3951 | 0.0000 | TRUE |

### 4.17.15 CD4- NKT cells - % of live leukocytes (Panel A)

|  | test | Difference | p\_value | Significance |
| --- | --- | --- | --- | --- |
| c2 | CTRl vs KO | 0.0572 | 0.8447 | FALSE |
| c3 | Female vs Male | 0.0414 | 0.9330 | FALSE |
| c4 | Female CTRL vs Female KO | 0.0608 | 0.9252 | FALSE |
| c5 | Male CTRL vs Male KO | 0.0536 | 0.9485 | FALSE |
| c6 | Female KO vs Male KO | 0.0378 | 0.0000 | TRUE |

### 4.17.16 CD4- NKT cells - % of NKT cells (Panel A)

|  | test | Difference | p\_value | Significance |
| --- | --- | --- | --- | --- |
| c2 | CTRl vs KO | 15.0381 | 0.0294 | TRUE |
| c3 | Female vs Male | -5.0018 | 0.7822 | FALSE |
| c4 | Female CTRL vs Female KO | 13.3315 | 0.2891 | FALSE |
| c5 | Male CTRL vs Male KO | 16.7446 | 0.1282 | FALSE |
| c6 | Female KO vs Male KO | -3.2953 | 0.0000 | TRUE |

### 4.17.17 CD4+ NKT cells - % of live leukocytes (Panel A)

|  | test | Difference | p\_value | Significance |
| --- | --- | --- | --- | --- |
| c2 | CTRl vs KO | -0.1944 | 0.3345 | FALSE |
| c3 | Female vs Male | 0.1211 | 0.7161 | FALSE |
| c4 | Female CTRL vs Female KO | -0.2013 | 0.5947 | FALSE |
| c5 | Male CTRL vs Male KO | -0.1874 | 0.6634 | FALSE |
| c6 | Female KO vs Male KO | 0.1280 | 0.0000 | TRUE |

### 4.17.18 CD4+ NKT cells - % of NKT cells (Panel A)

|  | test | Difference | p\_value | Significance |
| --- | --- | --- | --- | --- |
| c2 | CTRl vs KO | -15.3303 | 0.0242 | TRUE |
| c3 | Female vs Male | 4.7883 | 0.8011 | FALSE |
| c4 | Female CTRL vs Female KO | -13.7477 | 0.2585 | FALSE |
| c5 | Male CTRL vs Male KO | -16.9129 | 0.1192 | FALSE |
| c6 | Female KO vs Male KO | 3.2057 | 0.0000 | TRUE |

### 4.17.19 CD4+ T cells - % of T cells (Panel A)

|  | test | Difference | p\_value | Significance |
| --- | --- | --- | --- | --- |
| c2 | CTRl vs KO | 3.1544 | 0.3672 | FALSE |
| c3 | Female vs Male | 1.1782 | 0.9282 | FALSE |
| c4 | Female CTRL vs Female KO | 2.5679 | 0.7752 | FALSE |
| c5 | Male CTRL vs Male KO | 3.7410 | 0.5344 | FALSE |
| c6 | Female KO vs Male KO | 1.7648 | 0.0000 | TRUE |

### 4.17.20 CD4+ T helper cells - % of CD4 T cells (Panel A)

|  | test | Difference | p\_value | Significance |
| --- | --- | --- | --- | --- |
| c2 | CTRl vs KO | 0.0956 | 0.9999 | FALSE |
| c3 | Female vs Male | 0.3681 | 0.9924 | FALSE |
| c4 | Female CTRL vs Female KO | 0.1193 | 0.9999 | FALSE |
| c5 | Male CTRL vs Male KO | 0.0720 | 1.0000 | FALSE |
| c6 | Female KO vs Male KO | 0.3445 | 0.0010 | TRUE |

### 4.17.21 CD4+ T helper cells - %oflive leukocytes (Panel A

|  | test | Difference | p\_value | Significance |
| --- | --- | --- | --- | --- |
| c2 | CTRl vs KO | 2.0037 | 0.2942 | FALSE |
| c3 | Female vs Male | 1.0519 | 0.7873 | FALSE |
| c4 | Female CTRL vs Female KO | 1.7101 | 0.6933 | FALSE |
| c5 | Male CTRL vs Male KO | 2.2972 | 0.4981 | FALSE |
| c6 | Female KO vs Male KO | 1.3455 | 0.0000 | TRUE |

### 4.17.22 CD8+ T cells - % of live leukocytes (Panel A)

|  | test | Difference | p\_value | Significance |
| --- | --- | --- | --- | --- |
| c2 | CTRl vs KO | 0.5348 | 0.9706 | FALSE |
| c3 | Female vs Male | 0.2027 | 0.9983 | FALSE |
| c4 | Female CTRL vs Female KO | 0.4779 | 0.9919 | FALSE |
| c5 | Male CTRL vs Male KO | 0.5916 | 0.9859 | FALSE |
| c6 | Female KO vs Male KO | 0.2596 | 0.0934 | FALSE |

### 4.17.23 CD8+ T cells - % of T cells (Panel A)

|  | test | Difference | p\_value | Significance |
| --- | --- | --- | --- | --- |
| c2 | CTRl vs KO | -1.7070 | 0.8359 | FALSE |
| c3 | Female vs Male | -1.4408 | 0.8930 | FALSE |
| c4 | Female CTRL vs Female KO | -1.0245 | 0.9838 | FALSE |
| c5 | Male CTRL vs Male KO | -2.3894 | 0.8444 | FALSE |
| c6 | Female KO vs Male KO | -2.1232 | 0.0000 | TRUE |

### 4.17.24 Conventional DC - % of live leukocytes (Panel B)

|  | test | Difference | p\_value | Significance |
| --- | --- | --- | --- | --- |
| c2 | CTRl vs KO | 0.2662 | 0.8069 | FALSE |
| c3 | Female vs Male | -0.2075 | 0.8966 | FALSE |
| c4 | Female CTRL vs Female KO | 0.4371 | 0.7206 | FALSE |
| c5 | Male CTRL vs Male KO | 0.0954 | 0.9959 | FALSE |
| c6 | Female KO vs Male KO | -0.3784 | 0.0000 | TRUE |

### 4.17.25 dKlrg1+ gd T cells - % of live leukocytes (Panel A

ERROR : contrasts can be applied only to factors with 2 or more levels

### 4.17.26 Effector CD4- NKT c. - % of CD4- NKT c. (Panel A)

|  | test | Difference | p\_value | Significance |
| --- | --- | --- | --- | --- |
| c2 | CTRl vs KO | -15.5575 | 0.1426 | FALSE |
| c3 | Female vs Male | -0.6333 | 0.9998 | FALSE |
| c4 | Female CTRL vs Female KO | -18.4950 | 0.2681 | FALSE |
| c5 | Male CTRL vs Male KO | -12.6200 | 0.6049 | FALSE |
| c6 | Female KO vs Male KO | 2.3042 | 0.0000 | TRUE |

### 4.17.27 Effector CD4- NKT cells - % of live (Panel A)

|  | test | Difference | p\_value | Significance |
| --- | --- | --- | --- | --- |
| c2 | CTRl vs KO | -0.0265 | 0.8245 | FALSE |
| c3 | Female vs Male | 0.0124 | 0.9774 | FALSE |
| c4 | Female CTRL vs Female KO | -0.0391 | 0.7986 | FALSE |
| c5 | Male CTRL vs Male KO | -0.0138 | 0.9891 | FALSE |
| c6 | Female KO vs Male KO | 0.0250 | 0.0000 | TRUE |

### 4.17.28 Effector CD4+ NKT c. - % of CD4+ NKT c. (Panel A)

|  | test | Difference | p\_value | Significance |
| --- | --- | --- | --- | --- |
| c2 | CTRl vs KO | 3.2541 | 0.8715 | FALSE |
| c3 | Female vs Male | 0.1194 | 1.0000 | FALSE |
| c4 | Female CTRL vs Female KO | 2.4348 | 0.9773 | FALSE |
| c5 | Male CTRL vs Male KO | 4.0735 | 0.9074 | FALSE |
| c6 | Female KO vs Male KO | 0.9387 | 0.0026 | TRUE |

### 4.17.29 Effector CD4+ NKT cells - % of live (Panel A)

|  | test | Difference | p\_value | Significance |
| --- | --- | --- | --- | --- |
| c2 | CTRl vs KO | -0.1690 | 0.4321 | FALSE |
| c3 | Female vs Male | 0.1126 | 0.7416 | FALSE |
| c4 | Female CTRL vs Female KO | -0.1783 | 0.6627 | FALSE |
| c5 | Male CTRL vs Male KO | -0.1597 | 0.7467 | FALSE |
| c6 | Female KO vs Male KO | 0.1220 | 0.0000 | TRUE |

### 4.17.30 Effector CD4+ T help c.-% of CD4+ T help (Panel A)

|  | test | Difference | p\_value | Significance |
| --- | --- | --- | --- | --- |
| c2 | CTRl vs KO | -5.6615 | 0.2324 | FALSE |
| c3 | Female vs Male | -2.6416 | 0.8085 | FALSE |
| c4 | Female CTRL vs Female KO | -7.2157 | 0.2937 | FALSE |
| c5 | Male CTRL vs Male KO | -4.1072 | 0.7757 | FALSE |
| c6 | Female KO vs Male KO | -1.0873 | 0.0549 | FALSE |

### 4.17.31 Effector CD4+ T helper cells - % of live (Panel A)

|  | test | Difference | p\_value | Significance |
| --- | --- | --- | --- | --- |
| c2 | CTRl vs KO | -0.2245 | 0.9315 | FALSE |
| c3 | Female vs Male | -0.1922 | 0.9551 | FALSE |
| c4 | Female CTRL vs Female KO | -0.6339 | 0.6152 | FALSE |
| c5 | Male CTRL vs Male KO | 0.1850 | 0.9858 | FALSE |
| c6 | Female KO vs Male KO | 0.2172 | 0.0000 | TRUE |

### 4.17.32 Effector CD8+ T cells - % of CD8+ T cells (Panel A

|  | test | Difference | p\_value | Significance |
| --- | --- | --- | --- | --- |
| c2 | CTRl vs KO | 0.3132 | 0.9936 | FALSE |
| c3 | Female vs Male | -1.2064 | 0.7432 | FALSE |
| c4 | Female CTRL vs Female KO | -0.5191 | 0.9888 | FALSE |
| c5 | Male CTRL vs Male KO | 1.1455 | 0.9124 | FALSE |
| c6 | Female KO vs Male KO | -0.3741 | 0.1618 | FALSE |

### 4.17.33 Effector CD8+ T cells - %oflive leukocytes (Pane A

|  | test | Difference | p\_value | Significance |
| --- | --- | --- | --- | --- |
| c2 | CTRl vs KO | 0.0944 | 0.8481 | FALSE |
| c3 | Female vs Male | -0.1513 | 0.5617 | FALSE |
| c4 | Female CTRL vs Female KO | -0.0185 | 0.9994 | FALSE |
| c5 | Male CTRL vs Male KO | 0.2072 | 0.6048 | FALSE |
| c6 | Female KO vs Male KO | -0.0384 | 0.0451 | TRUE |

### 4.17.34 Effector gd T cells - % of live leukocytes (Panel

ERROR : contrasts can be applied only to factors with 2 or more levels

### 4.17.35 Effector gdT cells - % of gdT ce (Panel A)

ERROR : contrasts can be applied only to factors with 2 or more levels

### 4.17.36 Effector NK cells - % of live leukocytes (Panel A)

|  | test | Difference | p\_value | Significance |
| --- | --- | --- | --- | --- |
| c2 | CTRl vs KO | 0.0016 | 1.0000 | FALSE |
| c3 | Female vs Male | 0.0555 | 0.8936 | FALSE |
| c4 | Female CTRL vs Female KO | 0.0303 | 0.9924 | FALSE |
| c5 | Male CTRL vs Male KO | -0.0270 | 0.9952 | FALSE |
| c6 | Female KO vs Male KO | 0.0269 | 0.0309 | TRUE |

### 4.17.37 Effector NK cells - % of NK cells (Panel A)

|  | test | Difference | p\_value | Significance |
| --- | --- | --- | --- | --- |
| c2 | CTRl vs KO | 2.2153 | 0.8055 | FALSE |
| c3 | Female vs Male | 0.4945 | 0.9971 | FALSE |
| c4 | Female CTRL vs Female KO | 1.2658 | 0.9827 | FALSE |
| c5 | Male CTRL vs Male KO | 3.1648 | 0.8068 | FALSE |
| c6 | Female KO vs Male KO | 1.4440 | 0.0000 | TRUE |

### 4.17.38 Effector Treg cells - % of live leukocytes (Panel

|  | test | Difference | p\_value | Significance |
| --- | --- | --- | --- | --- |
| c2 | CTRl vs KO | -0.1799 | 0.3351 | FALSE |
| c3 | Female vs Male | -0.0339 | 0.9886 | FALSE |
| c4 | Female CTRL vs Female KO | -0.2415 | 0.3758 | FALSE |
| c5 | Male CTRL vs Male KO | -0.1184 | 0.8614 | FALSE |
| c6 | Female KO vs Male KO | 0.0277 | 0.0007 | TRUE |

### 4.17.39 Effector Treg cells - % of Treg cells (Panel A)

|  | test | Difference | p\_value | Significance |
| --- | --- | --- | --- | --- |
| c2 | CTRl vs KO | -20.4882 | 0.0000 | TRUE |
| c3 | Female vs Male | -3.3479 | 0.8496 | FALSE |
| c4 | Female CTRL vs Female KO | -23.3528 | 0.0005 | TRUE |
| c5 | Male CTRL vs Male KO | -17.6236 | 0.0192 | TRUE |
| c6 | Female KO vs Male KO | -0.4833 | 0.6048 | FALSE |

### 4.17.40 Eosinophils - % of live leukocytes (Panel B)

|  | test | Difference | p\_value | Significance |
| --- | --- | --- | --- | --- |
| c2 | CTRl vs KO | -0.1980 | 0.7096 | FALSE |
| c3 | Female vs Male | -0.1728 | 0.7863 | FALSE |
| c4 | Female CTRL vs Female KO | -0.3194 | 0.6021 | FALSE |
| c5 | Male CTRL vs Male KO | -0.0767 | 0.9916 | FALSE |
| c6 | Female KO vs Male KO | -0.0515 | 0.0833 | FALSE |

### 4.17.41 Follicular B cells - % of B cells (Panel B)

|  | test | Difference | p\_value | Significance |
| --- | --- | --- | --- | --- |
| c2 | CTRl vs KO | -6.7095 | 0.0305 | TRUE |
| c3 | Female vs Male | -3.7217 | 0.4053 | FALSE |
| c4 | Female CTRL vs Female KO | -8.2045 | 0.0731 | FALSE |
| c5 | Male CTRL vs Male KO | -5.2146 | 0.4258 | FALSE |
| c6 | Female KO vs Male KO | -2.2268 | 0.0000 | TRUE |

### 4.17.42 Follicular B cells - % of B cells LIVE (Panel B)

|  | test | Difference | p\_value | Significance |
| --- | --- | --- | --- | --- |
| c2 | CTRl vs KO | 0.5647 | 0.9958 | FALSE |
| c3 | Female vs Male | -4.3010 | 0.3111 | FALSE |
| c4 | Female CTRL vs Female KO | -1.1760 | 0.9864 | FALSE |
| c5 | Male CTRL vs Male KO | 2.3054 | 0.9148 | FALSE |
| c6 | Female KO vs Male KO | -2.5603 | 0.0000 | TRUE |

### 4.17.43 gammadelta T cells - % of live leukocytes (Panel A

ERROR : contrasts can be applied only to factors with 2 or more levels

### 4.17.44 Granulocytes - % of live leukocytes (Panel B)

|  | test | Difference | p\_value | Significance |
| --- | --- | --- | --- | --- |
| c2 | CTRl vs KO | -0.2492 | 0.9711 | FALSE |
| c3 | Female vs Male | -0.3022 | 0.9500 | FALSE |
| c4 | Female CTRL vs Female KO | -0.1091 | 0.9990 | FALSE |
| c5 | Male CTRL vs Male KO | -0.3892 | 0.9645 | FALSE |
| c6 | Female KO vs Male KO | -0.4422 | 0.0000 | TRUE |

### 4.17.45 Klrg1+ CD4- NKT c. - % of CD4- NKT c. (Panel A)

ERROR : contrasts can be applied only to factors with 2 or more levels

### 4.17.46 Klrg1+ CD4- NKT cells - % of live (Panel A)

ERROR : contrasts can be applied only to factors with 2 or more levels

### 4.17.47 Klrg1+ CD4+ NKT c. - % of CD4+ NKT c. (Panel A)

ERROR : contrasts can be applied only to factors with 2 or more levels

### 4.17.48 Klrg1+ CD4+ NKT cells - % of live (Panel A)

ERROR : contrasts can be applied only to factors with 2 or more levels

### 4.17.49 Klrg1+ CD4+ T help c. - % of CD4+ T help (Panel A

ERROR : contrasts can be applied only to factors with 2 or more levels

### 4.17.50 Klrg1+ CD4+ T helper cells - % of live (Panel A)

ERROR : contrasts can be applied only to factors with 2 or more levels

### 4.17.51 Klrg1+ CD8 T cells - % of CD8+ T cells (Panel A)

ERROR : contrasts can be applied only to factors with 2 or more levels

### 4.17.52 Klrg1+ CD8 T cells - % of live leukocytes (Panel A

ERROR : contrasts can be applied only to factors with 2 or more levels

### 4.17.53 Klrg1+ gdT cells - % of gd T cell (Panel A)

ERROR : contrasts can be applied only to factors with 2 or more levels

### 4.17.54 Klrg1+ NK cells - % of live leukocytes (Panel A)

ERROR : contrasts can be applied only to factors with 2 or more levels

### 4.17.55 Klrg1+ NK cells - % of NK cells (Panel A)

ERROR : contrasts can be applied only to factors with 2 or more levels

### 4.17.56 Klrg1+ Treg cells - % of live leukocytes (Panel A)

ERROR : contrasts can be applied only to factors with 2 or more levels

### 4.17.57 Klrg1+ Treg cells - % of Treg cells (Panel A)

ERROR : contrasts can be applied only to factors with 2 or more levels

### 4.17.58 Live leukocytes (Panel A) - % of total events

|  | test | Difference | p\_value | Significance |
| --- | --- | --- | --- | --- |
| c2 | CTRl vs KO | -9.7012 | 0.5342 | FALSE |
| c3 | Female vs Male | -6.8658 | 0.7734 | FALSE |
| c4 | Female CTRL vs Female KO | -16.7979 | 0.3455 | FALSE |
| c5 | Male CTRL vs Male KO | -2.6046 | 0.9942 | FALSE |
| c6 | Female KO vs Male KO | 0.2309 | 0.9831 | FALSE |

### 4.17.59 Live leukocytes (Panel B) - % of total events

|  | test | Difference | p\_value | Significance |
| --- | --- | --- | --- | --- |
| c2 | CTRl vs KO | -15.3779 | 0.1140 | FALSE |
| c3 | Female vs Male | -7.1483 | 0.7160 | FALSE |
| c4 | Female CTRL vs Female KO | -23.2130 | 0.0753 | FALSE |
| c5 | Male CTRL vs Male KO | -7.5429 | 0.8635 | FALSE |
| c6 | Female KO vs Male KO | 0.6868 | 0.6973 | FALSE |

### 4.17.60 Ly6C+ CD11b- NK cells - % of live (Panel B)

|  | test | Difference | p\_value | Significance |
| --- | --- | --- | --- | --- |
| c2 | CTRl vs KO | -0.2701 | 0.0021 | TRUE |
| c3 | Female vs Male | -0.0093 | 0.9993 | FALSE |
| c4 | Female CTRL vs Female KO | -0.2408 | 0.0952 | FALSE |
| c5 | Male CTRL vs Male KO | -0.2994 | 0.0269 | TRUE |
| c6 | Female KO vs Male KO | -0.0386 | 0.0000 | TRUE |

### 4.17.61 Ly6C+ CD11b- NK cells - % of NK cells (Panel B)

|  | test | Difference | p\_value | Significance |
| --- | --- | --- | --- | --- |
| c2 | CTRl vs KO | -5.9587 | 0.0064 | TRUE |
| c3 | Female vs Male | -0.4174 | 0.9954 | FALSE |
| c4 | Female CTRL vs Female KO | -5.9225 | 0.0908 | FALSE |
| c5 | Male CTRL vs Male KO | -5.9949 | 0.0918 | FALSE |
| c6 | Female KO vs Male KO | -0.4536 | 0.0101 | TRUE |

### 4.17.62 Ly6C+ CD11b+ NK cells - % of live (Panel B)

|  | test | Difference | p\_value | Significance |
| --- | --- | --- | --- | --- |
| c2 | CTRl vs KO | -0.1180 | 0.9255 | FALSE |
| c3 | Female vs Male | 0.1586 | 0.8376 | FALSE |
| c4 | Female CTRL vs Female KO | -0.0378 | 0.9990 | FALSE |
| c5 | Male CTRL vs Male KO | -0.1982 | 0.8858 | FALSE |
| c6 | Female KO vs Male KO | 0.0784 | 0.0010 | TRUE |

### 4.17.63 Ly6C+ CD11b+ NK cells - % of NK cells (Panel B)

|  | test | Difference | p\_value | Significance |
| --- | --- | --- | --- | --- |
| c2 | CTRl vs KO | 2.1973 | 0.8812 | FALSE |
| c3 | Female vs Male | 2.1289 | 0.8897 | FALSE |
| c4 | Female CTRL vs Female KO | 1.5684 | 0.9817 | FALSE |
| c5 | Male CTRL vs Male KO | 2.8262 | 0.9091 | FALSE |
| c6 | Female KO vs Male KO | 2.7577 | 0.0000 | TRUE |

### 4.17.64 Ly6C+ NKT cells - % of live leukocytes (Panel B)

|  | test | Difference | p\_value | Significance |
| --- | --- | --- | --- | --- |
| c2 | CTRl vs KO | 0.0873 | 0.8537 | FALSE |
| c3 | Female vs Male | 0.1185 | 0.6965 | FALSE |
| c4 | Female CTRL vs Female KO | 0.1339 | 0.8182 | FALSE |
| c5 | Male CTRL vs Male KO | 0.0408 | 0.9935 | FALSE |
| c6 | Female KO vs Male KO | 0.0720 | 0.0000 | TRUE |

### 4.17.65 Ly6C+ NKT cells - % of NKT cells (Panel B)

|  | test | Difference | p\_value | Significance |
| --- | --- | --- | --- | --- |
| c2 | CTRl vs KO | 15.4026 | 0.0862 | FALSE |
| c3 | Female vs Male | -2.9231 | 0.9683 | FALSE |
| c4 | Female CTRL vs Female KO | 14.4548 | 0.3835 | FALSE |
| c5 | Male CTRL vs Male KO | 16.3503 | 0.2867 | FALSE |
| c6 | Female KO vs Male KO | -1.9753 | 0.0006 | TRUE |

### 4.17.66 Macrophages- % of live leukocytes (Panel B)

ERROR : contrasts can be applied only to factors with 2 or more levels

### 4.17.67 Marginal zone B cells - % B cells LIVE (Panel B)

|  | test | Difference | p\_value | Significance |
| --- | --- | --- | --- | --- |
| c2 | CTRl vs KO | 5.0161 | 0.0000 | TRUE |
| c3 | Female vs Male | -0.0457 | 0.9999 | FALSE |
| c4 | Female CTRL vs Female KO | 4.5431 | 0.0004 | TRUE |
| c5 | Male CTRL vs Male KO | 5.4892 | 0.0000 | TRUE |
| c6 | Female KO vs Male KO | 0.4274 | 0.0000 | TRUE |

### 4.17.68 Marginal zone B cells - % of B cells (Panel B)

|  | test | Difference | p\_value | Significance |
| --- | --- | --- | --- | --- |
| c2 | CTRl vs KO | 8.5803 | 0.0000 | TRUE |
| c3 | Female vs Male | 0.9935 | 0.9239 | FALSE |
| c4 | Female CTRL vs Female KO | 8.2618 | 0.0020 | TRUE |
| c5 | Male CTRL vs Male KO | 8.8988 | 0.0010 | TRUE |
| c6 | Female KO vs Male KO | 1.3120 | 0.0000 | TRUE |

### 4.17.69 Monocytes - % of live leukocytes (Panel B)

|  | test | Difference | p\_value | Significance |
| --- | --- | --- | --- | --- |
| c2 | CTRl vs KO | -0.6276 | 0.1197 | FALSE |
| c3 | Female vs Male | -0.3352 | 0.6274 | FALSE |
| c4 | Female CTRL vs Female KO | -0.6445 | 0.3380 | FALSE |
| c5 | Male CTRL vs Male KO | -0.6107 | 0.4419 | FALSE |
| c6 | Female KO vs Male KO | -0.3183 | 0.0000 | TRUE |

### 4.17.70 Naive CD8+ T cells - % of CD8+ T cells (Panel A)

|  | test | Difference | p\_value | Significance |
| --- | --- | --- | --- | --- |
| c2 | CTRl vs KO | 5.8056 | 0.6014 | FALSE |
| c3 | Female vs Male | 5.8272 | 0.5965 | FALSE |
| c4 | Female CTRL vs Female KO | 6.3326 | 0.7645 | FALSE |
| c5 | Male CTRL vs Male KO | 5.2786 | 0.8590 | FALSE |
| c6 | Female KO vs Male KO | 5.3002 | 0.0000 | TRUE |

### 4.17.71 Naive CD8+ T cells - % of live leukocytes (Panel A

|  | test | Difference | p\_value | Significance |
| --- | --- | --- | --- | --- |
| c2 | CTRl vs KO | 0.9529 | 0.7468 | FALSE |
| c3 | Female vs Male | 0.8679 | 0.7953 | FALSE |
| c4 | Female CTRL vs Female KO | 1.0450 | 0.8578 | FALSE |
| c5 | Male CTRL vs Male KO | 0.8607 | 0.9207 | FALSE |
| c6 | Female KO vs Male KO | 0.7757 | 0.0000 | TRUE |

### 4.17.72 NK cells (Panel A) - % of live l(Panel A)

|  | test | Difference | p\_value | Significance |
| --- | --- | --- | --- | --- |
| c2 | CTRl vs KO | -0.4961 | 0.4799 | FALSE |
| c3 | Female vs Male | 0.1526 | 0.9707 | FALSE |
| c4 | Female CTRL vs Female KO | -0.1625 | 0.9863 | FALSE |
| c5 | Male CTRL vs Male KO | -0.8297 | 0.3476 | FALSE |
| c6 | Female KO vs Male KO | -0.1810 | 0.0003 | TRUE |

### 4.17.73 NK cells (Panel B) - % of live leukocytes (Panel B

|  | test | Difference | p\_value | Significance |
| --- | --- | --- | --- | --- |
| c2 | CTRl vs KO | -0.6185 | 0.3327 | FALSE |
| c3 | Female vs Male | 0.2241 | 0.9266 | FALSE |
| c4 | Female CTRL vs Female KO | -0.2317 | 0.9675 | FALSE |
| c5 | Male CTRL vs Male KO | -1.0053 | 0.2317 | FALSE |
| c6 | Female KO vs Male KO | -0.1627 | 0.0058 | TRUE |

### 4.17.74 NKT cells (panel A) - % of live (Panel A)

|  | test | Difference | p\_value | Significance |
| --- | --- | --- | --- | --- |
| c2 | CTRl vs KO | -0.1385 | 0.8238 | FALSE |
| c3 | Female vs Male | 0.1666 | 0.7261 | FALSE |
| c4 | Female CTRL vs Female KO | -0.1410 | 0.9228 | FALSE |
| c5 | Male CTRL vs Male KO | -0.1361 | 0.9333 | FALSE |
| c6 | Female KO vs Male KO | 0.1691 | 0.0000 | TRUE |

### 4.17.75 NKT cells (panel B) - % of live leukocytes (Panel

|  | test | Difference | p\_value | Significance |
| --- | --- | --- | --- | --- |
| c2 | CTRl vs KO | -0.1348 | 0.8846 | FALSE |
| c3 | Female vs Male | 0.2496 | 0.5304 | FALSE |
| c4 | Female CTRL vs Female KO | -0.0756 | 0.9912 | FALSE |
| c5 | Male CTRL vs Male KO | -0.1940 | 0.8815 | FALSE |
| c6 | Female KO vs Male KO | 0.1904 | 0.0000 | TRUE |

### 4.17.76 Plasmacytoid DC- % of live leukocytes (Panel B)

ERROR : contrasts can be applied only to factors with 2 or more levels

### 4.17.77 Resting CD4- NKT c. - % of CD4- NKT c. (Panel A)

|  | test | Difference | p\_value | Significance |
| --- | --- | --- | --- | --- |
| c2 | CTRl vs KO | 14.9179 | 0.1743 | FALSE |
| c3 | Female vs Male | 0.1351 | 1.0000 | FALSE |
| c4 | Female CTRL vs Female KO | 17.5203 | 0.3190 | FALSE |
| c5 | Male CTRL vs Male KO | 12.3155 | 0.6278 | FALSE |
| c6 | Female KO vs Male KO | -2.4672 | 0.0000 | TRUE |

### 4.17.78 Resting CD4- NKT cells - % of live (Panel A)

|  | test | Difference | p\_value | Significance |
| --- | --- | --- | --- | --- |
| c2 | CTRl vs KO | 0.0811 | 0.4002 | FALSE |
| c3 | Female vs Male | 0.0271 | 0.9523 | FALSE |
| c4 | Female CTRL vs Female KO | 0.0970 | 0.5439 | FALSE |
| c5 | Male CTRL vs Male KO | 0.0653 | 0.8079 | FALSE |
| c6 | Female KO vs Male KO | 0.0113 | 0.0001 | TRUE |

### 4.17.79 Resting CD4+ NKT c. - % of CD4+ NKT c. (Panel A)

|  | test | Difference | p\_value | Significance |
| --- | --- | --- | --- | --- |
| c2 | CTRl vs KO | -3.7613 | 0.7858 | FALSE |
| c3 | Female vs Male | -0.8619 | 0.9965 | FALSE |
| c4 | Female CTRL vs Female KO | -3.6136 | 0.9189 | FALSE |
| c5 | Male CTRL vs Male KO | -3.9089 | 0.9023 | FALSE |
| c6 | Female KO vs Male KO | -1.0095 | 0.0004 | TRUE |

### 4.17.80 Resting CD4+ NKT cells - % of live (Panel A)

|  | test | Difference | p\_value | Significance |
| --- | --- | --- | --- | --- |
| c2 | CTRl vs KO | -0.0264 | 0.4826 | FALSE |
| c3 | Female vs Male | 0.0045 | 0.9948 | FALSE |
| c4 | Female CTRL vs Female KO | -0.0264 | 0.7395 | FALSE |
| c5 | Male CTRL vs Male KO | -0.0265 | 0.7425 | FALSE |
| c6 | Female KO vs Male KO | 0.0045 | 0.0000 | TRUE |

### 4.17.81 Resting CD4+ T help c. - % of CD4+ T help (Panel

|  | test | Difference | p\_value | Significance |
| --- | --- | --- | --- | --- |
| c2 | CTRl vs KO | 3.8719 | 0.7477 | FALSE |
| c3 | Female vs Male | 2.1841 | 0.9414 | FALSE |
| c4 | Female CTRL vs Female KO | 5.2461 | 0.7581 | FALSE |
| c5 | Male CTRL vs Male KO | 2.4976 | 0.9698 | FALSE |
| c6 | Female KO vs Male KO | 0.8098 | 0.3712 | FALSE |

### 4.17.82 Resting CD4+ T helper cells - % of live (Panel A)

|  | test | Difference | p\_value | Significance |
| --- | --- | --- | --- | --- |
| c2 | CTRl vs KO | 1.8623 | 0.2776 | FALSE |
| c3 | Female vs Male | 1.1566 | 0.6752 | FALSE |
| c4 | Female CTRL vs Female KO | 1.9536 | 0.5184 | FALSE |
| c5 | Male CTRL vs Male KO | 1.7710 | 0.6356 | FALSE |
| c6 | Female KO vs Male KO | 1.0653 | 0.0000 | TRUE |

### 4.17.83 Resting CD8+ T cells - % of CD8+ T cells (Panel A)

|  | test | Difference | p\_value | Significance |
| --- | --- | --- | --- | --- |
| c2 | CTRl vs KO | -7.0176 | 0.3214 | FALSE |
| c3 | Female vs Male | -3.4392 | 0.8330 | FALSE |
| c4 | Female CTRL vs Female KO | -4.8868 | 0.8274 | FALSE |
| c5 | Male CTRL vs Male KO | -9.1485 | 0.4016 | FALSE |
| c6 | Female KO vs Male KO | -5.5700 | 0.0000 | TRUE |

### 4.17.84 Resting CD8+ T cells - %of live (Panel A)

|  | test | Difference | p\_value | Significance |
| --- | --- | --- | --- | --- |
| c2 | CTRl vs KO | -0.6925 | 0.6556 | FALSE |
| c3 | Female vs Male | -0.3345 | 0.9433 | FALSE |
| c4 | Female CTRL vs Female KO | -0.4508 | 0.9497 | FALSE |
| c5 | Male CTRL vs Male KO | -0.9341 | 0.6920 | FALSE |
| c6 | Female KO vs Male KO | -0.5762 | 0.0000 | TRUE |

### 4.17.85 Resting gd T cells - % of gd T cells (Panel A)

ERROR : contrasts can be applied only to factors with 2 or more levels

### 4.17.86 Resting gd T cells - % of live leukocytes (Panel A

ERROR : contrasts can be applied only to factors with 2 or more levels

### 4.17.87 Resting NK cells - % of live leukocytes (Panel A)

|  | test | Difference | p\_value | Significance |
| --- | --- | --- | --- | --- |
| c2 | CTRl vs KO | -0.4454 | 0.5852 | FALSE |
| c3 | Female vs Male | 0.0449 | 0.9992 | FALSE |
| c4 | Female CTRL vs Female KO | -0.1645 | 0.9866 | FALSE |
| c5 | Male CTRL vs Male KO | -0.7263 | 0.4792 | FALSE |
| c6 | Female KO vs Male KO | -0.2360 | 0.0000 | TRUE |

### 4.17.88 Resting NK cells - % of NK cells (Panel A)

|  | test | Difference | p\_value | Significance |
| --- | --- | --- | --- | --- |
| c2 | CTRl vs KO | -1.0555 | 0.9910 | FALSE |
| c3 | Female vs Male | -2.0525 | 0.9394 | FALSE |
| c4 | Female CTRL vs Female KO | -0.8113 | 0.9985 | FALSE |
| c5 | Male CTRL vs Male KO | -1.2996 | 0.9942 | FALSE |
| c6 | Female KO vs Male KO | -2.2966 | 0.0000 | TRUE |

### 4.17.89 Resting Treg cells - % of live (Panel A)

|  | test | Difference | p\_value | Significance |
| --- | --- | --- | --- | --- |
| c2 | CTRl vs KO | 0.3627 | 0.0006 | TRUE |
| c3 | Female vs Male | 0.0399 | 0.9709 | FALSE |
| c4 | Female CTRL vs Female KO | 0.3807 | 0.0177 | TRUE |
| c5 | Male CTRL vs Male KO | 0.3447 | 0.0440 | TRUE |
| c6 | Female KO vs Male KO | 0.0219 | 0.0320 | TRUE |

### 4.17.90 Resting Treg cells - % of Treg cells (Panel A)

|  | test | Difference | p\_value | Significance |
| --- | --- | --- | --- | --- |
| c2 | CTRl vs KO | 21.4835 | 0.0000 | TRUE |
| c3 | Female vs Male | 2.4160 | 0.9434 | FALSE |
| c4 | Female CTRL vs Female KO | 23.6259 | 0.0010 | TRUE |
| c5 | Male CTRL vs Male KO | 19.3411 | 0.0125 | TRUE |
| c6 | Female KO vs Male KO | 0.2736 | 0.9093 | FALSE |

### 4.17.91 T cells (Panel A) - % of live leukocytes (Panel A)

|  | test | Difference | p\_value | Significance |
| --- | --- | --- | --- | --- |
| c2 | CTRl vs KO | 2.3759 | 0.6907 | FALSE |
| c3 | Female vs Male | 1.3838 | 0.9179 | FALSE |
| c4 | Female CTRL vs Female KO | 1.9379 | 0.9165 | FALSE |
| c5 | Male CTRL vs Male KO | 2.8139 | 0.8013 | FALSE |
| c6 | Female KO vs Male KO | 1.8219 | 0.0000 | TRUE |

### 4.17.92 T cells (panel B) - % of live leukocytes (Panel B)

|  | test | Difference | p\_value | Significance |
| --- | --- | --- | --- | --- |
| c2 | CTRl vs KO | 3.0909 | 0.6081 | FALSE |
| c3 | Female vs Male | 2.5812 | 0.7310 | FALSE |
| c4 | Female CTRL vs Female KO | 3.6681 | 0.7194 | FALSE |
| c5 | Male CTRL vs Male KO | 2.5138 | 0.8970 | FALSE |
| c6 | Female KO vs Male KO | 2.0040 | 0.0000 | TRUE |

### 4.17.93 Treg cells - % of CD4 T cells (Panel A)

|  | test | Difference | p\_value | Significance |
| --- | --- | --- | --- | --- |
| c2 | CTRl vs KO | -0.1310 | 0.9996 | FALSE |
| c3 | Female vs Male | -0.3832 | 0.9906 | FALSE |
| c4 | Female CTRL vs Female KO | -0.1591 | 0.9998 | FALSE |
| c5 | Male CTRL vs Male KO | -0.1030 | 0.9999 | FALSE |
| c6 | Female KO vs Male KO | -0.3552 | 0.0008 | TRUE |

### 4.17.94 Treg cells - % of live leukocytes (Panel A)

|  | test | Difference | p\_value | Significance |
| --- | --- | --- | --- | --- |
| c2 | CTRl vs KO | 0.1823 | 0.7362 | FALSE |
| c3 | Female vs Male | 0.0178 | 0.9996 | FALSE |
| c4 | Female CTRL vs Female KO | 0.1465 | 0.9360 | FALSE |
| c5 | Male CTRL vs Male KO | 0.2181 | 0.8244 | FALSE |
| c6 | Female KO vs Male KO | 0.0536 | 0.0002 | TRUE |

### 4.17.95 VM T cells - % of CD8+ T cells (Panel B)

ERROR : contrasts can be applied only to factors with 2 or more levels

### 4.17.96 VM T cells - % of live leukocytes (Panel B)

ERROR : contrasts can be applied only to factors with 2 or more levels

## 4.18 Gross Pathology

**Number of animals**

| STRAIN | GENDER | count |
| --- | --- | --- |
| AMB-YLF-GGG | female | 8 |
| AMB-YLF-GGG | male | 7 |
| C57Bl/6NCrl | female | 918 |
| C57Bl/6NCrl | male | 874 |

### 4.18.1 Adrenal gland

|  | female AMB-YLF-GGG | female C57Bl/6NCrl | male AMB-YLF-GGG |
| --- | --- | --- | --- |
| female C57Bl/6NCrl | 1 | NA | NA |
| male AMB-YLF-GGG | 1 | 1.0000 | NA |
| male C57Bl/6NCrl | 1 | 0.3752 | 1 |

### 4.18.2 Bone

|  | female AMB-YLF-GGG | female C57Bl/6NCrl | male AMB-YLF-GGG |
| --- | --- | --- | --- |
| female C57Bl/6NCrl | 1 | NA | NA |
| male AMB-YLF-GGG | 1 | 1 | NA |
| male C57Bl/6NCrl | 1 | 1 | 1 |

### 4.18.3 Bone marrow

### 4.18.4 Brain

### 4.18.5 Epididymis

|  | female C57Bl/6NCrl | male AMB-YLF-GGG |
| --- | --- | --- |
| male AMB-YLF-GGG | 1 | NA |
| male C57Bl/6NCrl | 1 | 1 |

### 4.18.6 Esophagus

|  | female AMB-YLF-GGG | female C57Bl/6NCrl | male AMB-YLF-GGG |
| --- | --- | --- | --- |
| female C57Bl/6NCrl | 1 | NA | NA |
| male AMB-YLF-GGG | 1 | 1 | NA |
| male C57Bl/6NCrl | 1 | 1 | 1 |

### 4.18.7 Eye with optic nerve

|  | female AMB-YLF-GGG | female C57Bl/6NCrl | male AMB-YLF-GGG |
| --- | --- | --- | --- |
| female C57Bl/6NCrl | 1 | NA | NA |
| male AMB-YLF-GGG | 1 | 1.0000 | NA |
| male C57Bl/6NCrl | 1 | 0.0266 | 1 |

### 4.18.8 Femur

|  | female AMB-YLF-GGG | female C57Bl/6NCrl | male AMB-YLF-GGG |
| --- | --- | --- | --- |
| female C57Bl/6NCrl | 1 | NA | NA |
| male AMB-YLF-GGG | 1 | 1 | NA |
| male C57Bl/6NCrl | 1 | 1 | 1 |

### 4.18.9 Gall bladder

|  | female AMB-YLF-GGG | female C57Bl/6NCrl | male AMB-YLF-GGG |
| --- | --- | --- | --- |
| female C57Bl/6NCrl | 1 | NA | NA |
| male AMB-YLF-GGG | 1 | 1 | NA |
| male C57Bl/6NCrl | 1 | 1 | 1 |

### 4.18.10 Heart

|  | female AMB-YLF-GGG | female C57Bl/6NCrl | male AMB-YLF-GGG |
| --- | --- | --- | --- |
| female C57Bl/6NCrl | 1 | NA | NA |
| male AMB-YLF-GGG | 1 | 1 | NA |
| male C57Bl/6NCrl | 1 | 0 | 1 |

### 4.18.11 Kidney

|  | female AMB-YLF-GGG | female C57Bl/6NCrl | male AMB-YLF-GGG |
| --- | --- | --- | --- |
| female C57Bl/6NCrl | 1 | NA | NA |
| male AMB-YLF-GGG | 1 | 1 | NA |
| male C57Bl/6NCrl | 1 | 1 | 1 |

### 4.18.12 Large intestine

|  | female AMB-YLF-GGG | female C57Bl/6NCrl | male AMB-YLF-GGG |
| --- | --- | --- | --- |
| female C57Bl/6NCrl | 1 | NA | NA |
| male AMB-YLF-GGG | 1 | 1 | NA |
| male C57Bl/6NCrl | 1 | 1 | 1 |

### 4.18.13 Liver

|  | female AMB-YLF-GGG | female C57Bl/6NCrl | male AMB-YLF-GGG |
| --- | --- | --- | --- |
| female C57Bl/6NCrl | 1 | NA | NA |
| male AMB-YLF-GGG | 1 | 1 | NA |
| male C57Bl/6NCrl | 1 | 1 | 1 |

### 4.18.14 Lung

|  | female AMB-YLF-GGG | female C57Bl/6NCrl | male AMB-YLF-GGG |
| --- | --- | --- | --- |
| female C57Bl/6NCrl | 1 | NA | NA |
| male AMB-YLF-GGG | 1 | 1.0000 | NA |
| male C57Bl/6NCrl | 1 | 0.6949 | 1 |

### 4.18.15 Lymph node

|  | female AMB-YLF-GGG | female C57Bl/6NCrl | male AMB-YLF-GGG |
| --- | --- | --- | --- |
| female C57Bl/6NCrl | 1 | NA | NA |
| male AMB-YLF-GGG | 1 | 1.0000 | NA |
| male C57Bl/6NCrl | 1 | 0.3819 | 1 |

### 4.18.16 Mammary gland

|  | female AMB-YLF-GGG | female C57Bl/6NCrl | male AMB-YLF-GGG |
| --- | --- | --- | --- |
| female C57Bl/6NCrl | 1 | NA | NA |
| male AMB-YLF-GGG | 1 | 1 | NA |
| male C57Bl/6NCrl | 1 | 1 | 1 |

### 4.18.17 Ovary

x

1

### 4.18.18 Pancreas

|  | female AMB-YLF-GGG | female C57Bl/6NCrl | male AMB-YLF-GGG |
| --- | --- | --- | --- |
| female C57Bl/6NCrl | 1 | NA | NA |
| male AMB-YLF-GGG | 1 | 1 | NA |
| male C57Bl/6NCrl | 1 | 1 | 1 |

### 4.18.19 Prostate

### 4.18.20 Salivary gland

### 4.18.21 Seminal vesicle

|  | female C57Bl/6NCrl | male AMB-YLF-GGG |
| --- | --- | --- |
| male AMB-YLF-GGG | 1 | NA |
| male C57Bl/6NCrl | 1 | 1 |

### 4.18.22 Skeletal muscle

### 4.18.23 Skin

|  | female AMB-YLF-GGG | female C57Bl/6NCrl | male AMB-YLF-GGG |
| --- | --- | --- | --- |
| female C57Bl/6NCrl | 1 | NA | NA |
| male AMB-YLF-GGG | 1 | 1.0000 | NA |
| male C57Bl/6NCrl | 1 | 0.0774 | 1 |

### 4.18.24 Small intestine

### 4.18.25 Spinal cord

### 4.18.26 Spleen

|  | female AMB-YLF-GGG | female C57Bl/6NCrl | male AMB-YLF-GGG |
| --- | --- | --- | --- |
| female C57Bl/6NCrl | 1 | NA | NA |
| male AMB-YLF-GGG | 1 | 1.0000 | NA |
| male C57Bl/6NCrl | 1 | 0.8479 | 1 |

### 4.18.27 Stomach

### 4.18.28 Testes

|  | female C57Bl/6NCrl | male AMB-YLF-GGG |
| --- | --- | --- |
| male AMB-YLF-GGG | 1 | NA |
| male C57Bl/6NCrl | 1 | 1 |

### 4.18.29 Thymus

|  | female AMB-YLF-GGG | female C57Bl/6NCrl | male AMB-YLF-GGG |
| --- | --- | --- | --- |
| female C57Bl/6NCrl | 1 | NA | NA |
| male AMB-YLF-GGG | 1 | 1 | NA |
| male C57Bl/6NCrl | 1 | 0 | 1 |

### 4.18.30 Thyroid

|  | female AMB-YLF-GGG | female C57Bl/6NCrl | male AMB-YLF-GGG |
| --- | --- | --- | --- |
| female C57Bl/6NCrl | 1 | NA | NA |
| male AMB-YLF-GGG | 1 | 1.0000 | NA |
| male C57Bl/6NCrl | 1 | 0.3383 | 1 |

### 4.18.31 Tooth

|  | female AMB-YLF-GGG | female C57Bl/6NCrl | male AMB-YLF-GGG |
| --- | --- | --- | --- |
| female C57Bl/6NCrl | 0 | NA | NA |
| male AMB-YLF-GGG | 1 | 0.000 | NA |
| male C57Bl/6NCrl | 0 | 0.075 | 0 |

### 4.18.32 Trachea

### 4.18.33 Urinary bladder

|  | female AMB-YLF-GGG | female C57Bl/6NCrl | male AMB-YLF-GGG |
| --- | --- | --- | --- |
| female C57Bl/6NCrl | 1 | NA | NA |
| male AMB-YLF-GGG | 1 | 1.0000 | NA |
| male C57Bl/6NCrl | 1 | 0.3383 | 1 |

### 4.18.34 Uterus

x

1
